# From molecular interaction to acute promyelocytic leukemia: Calculating leukemogenesis and remission from endogenous molecular-cellular network

**DOI:** 10.1038/srep24307

**Published:** 2016-04-21

**Authors:** Ruoshi Yuan, Xiaomei Zhu, Jerald P. Radich, Ping Ao

**Affiliations:** 1 Key Laboratory of Systems Biomedicine, Ministry of Education, Shanghai Center for Systems Biomedicine, Shanghai Jiao Tong University, Shanghai, 200240, China; 2 School of Biomedical Engineering, Shanghai Jiao Tong University, Shanghai, 200240, China; 3 GeneMath, 5525 27th Ave. N.E., Seattle, WA 98105, USA; 4 Fred Hutchinson Cancer Research Center, 1100 Fairview Avenue N., Seattle, WA 98109, USA

## Abstract

Acute promyelocytic leukemia (APL) remains the best example of a malignancy that can be cured clinically by differentiation therapy. We demonstrate that APL may emerge from a dynamical endogenous molecular-cellular network obtained from normal, non-cancerous molecular interactions such as signal transduction and translational regulation under physiological conditions. This unifying framework, which reproduces APL, normal progenitor, and differentiated granulocytic phenotypes as different robust states from the network dynamics, has the advantage to study transition between these states, i.e. critical drivers for leukemogenesis and targets for differentiation. The simulation results quantitatively reproduce microarray profiles of NB4 and HL60 cell lines in response to treatment and normal neutrophil differentiation, and lead to new findings such as biomarkers for APL and additional molecular targets for arsenic trioxide therapy. The modeling shows APL and normal states mutually suppress each other, both in “wiring” and in dynamical cooperation. Leukemogenesis and recovery under treatment may be a consequence of spontaneous or induced transitions between robust states, through “passes” or “dragging” by drug effects. Our approach rationalizes leukemic complexity and constructs a platform towards extending differentiation therapy by performing “dry” molecular biology experiments.

Leukemia is a common malignancy that may affect about 1.5 percent of a total population during their lifetime^1^. Despite improved survival rate, leukemia still carries a high mortality rate. Acute promyelocytic leukemia (APL) is a subset of acute myeloid leukemia that, unlike other forms of leukemia, can be successfully treated by therapy that causes granulocytic differentiation of leukemic blasts. All-trans-retinoic acid (ATRA) based therapy of APL has made a previously highly fatal disease to a highly curable one^2^. Although different drug targets have been investigated for differentiation therapy in other AMLs^3^, ATRA based therapy for APL remains the only clinically successful one.

APL is most frequently characterized by the t(15; 17) translocation, which causes the PML/RAR*α* gene fusion and chimeric protein^4^. Retinoids activate two classes of nuclear receptor proteins, the retinoic acid receptors (RARs, *α*, *β* and *γ*), and the retinoid X receptors (RXRs). RAR*α* interacts with RXR, and the RAR*α*-RXR heterodimer recruits corepressor or coactivator to differentially regulate transcription of its target genes. Retinoic acid releases the nuclear corepressor from the RAR/RXR complex results in eventual transcriptional activation of RAR-target genes. PML/RAR*α* acts as a constitutive repressor that is insensitive to physiological concentrations of retinoic acid. APL patients are treated with pharmacological doses of ATRA to overwhelm the leukemogenic potential of PML/RAR*α*.

Such a frequently cited straightforward interpretation of APL development leaves open questions^4^. Mechanism of PML/RAR*α* induced oncogenic transformation has been studied using various APL mouse models^5^,^6^,^7^. PML/RAR*α* is the only driving genetic event capable of initiating a typical APL disease when expressed in transgenic mice. However, the emergence of full-blown APL after initiation by PML/RAR*α* requires 12–14 months. This long latency was hypothesized as an indication for additional genetic/epigenetic changes in the progression to the full transformation of APL phenotype, in sequential to PML/RAR*α*^4^. Besides, it is shown that deregulation of the retinoic acid pathway is insufficient to initiate APL^7^. PML/RAR*α* transcript was detected in APL patients under long-term remission^8^, suggesting a complex relationship between phenotype and genotype. A recent experiment has shown that the reprogramed B-ALL (B-cell acute lymphoblastic leukemia) cells with BCR-ABL1 translocation appeared to lose their carcinogenicity^9^. Thus, molecular mechanisms other than mutation are important as well.

Arsenic trioxide (ATO) has been found to be effective in APL both as additive to ATRA, as well as monotherapy^10^. A natural explanation for the clinically observed *in vivo* synergistic effects of ATRA and ATO should be the collaboration among their distinct targeting molecular pathways. ATO was shown to degrade PML/RAR*α* mediated by sumolation^11^, an effect likely to be similar to ATRA treatment. Large scale screening of ATO response showed that most of genes affected by ATO were also affected by ATRA. Screening was able to identify effects of ATO such as reorganization of the cell nucleus and cytoplasmic structures, but not its impact on the multilayered regulatory levels significantly different from ATRA^12^. The exact role for ATO in ATRA-based therapy remains unclear.

Most receptors and molecular pathways are developmentally regulated. RAR*α* signaling was found to enhance the growth of the granulocyte-macrophage colony-stimulating factor (GM-CSF) dependent colonies derived from normal human bone marrow^13^. RAR*α* simultaneously decreases production of colonies representing other hematopoietic lineages including erythroid^13^. Interestingly, RAR*α* dependent clonal selection is also cell/cell contact dependent. In liquid suspension, ATRA not only enhances the generation of committed myeloid progenitors but also increases the production of more primitive hematopoietic precursors^13^. Therefore, task as simple as to understand the role of RAR*α* itself requires methods to accommodate complexity.

Considering these questions to be clarified, we explored APL in this paper from systems biology viewpoint. We constructed endogenous molecular-cellular network^14^ (see Fig. 1 for the procedure) for APL comprised of consolidated molecules and molecular pathways critical for normal hemopoietic development and physiology. The endogenous network consists of various feedback loops without absolute upstream or downstream, in contrast to “input-output” type of signal transduction modeling. The dynamics of this network has unique properties not possessed by individual pathways and modules, such as autonomy, multistability, robustness, adaptivity, and cooperativeness. The dynamical structure of the APL network was found to support the hypothesis that APL is a robust state formed by molecular interactions^14^. The leukemogenesis and recovery by differentiation therapy are straightforwardly interpreted as transitions between APL and normal states. New biomarkers and drug targets were obtained from modeling results. A mechanistic and quantitative model of APL is achieved here.

## Results

### APL Network

Endogenous network for APL was constructed with 8 modules, 81 nodes, and 416 interaction based on 357 references listed in Fig. 2 and Supplementary Table I. Modules include cell cycle, apoptosis, growth factors, differentiation, immune response, stress response, extracellular matrix, and nuclear receptors. Nodes in each module consist of individual molecules and consolidated pathways (e.g., SHH denotes sonic hedgehog pathway). The APL network presented in this paper is of a minimized core network. It is able to be enlarged to accommodate more details.

### Attractors in the network: APL as a robust state determined by network

In a molecular interaction network, multiple feedbacks coordinate molecular activities. As a result, only limited states of the network (activity profiles of the molecules) determined by network structure are robust ones^14^,^15^. Mathematically, they correspond to attractors. By solving differential equations for the APL network dynamics from random initial conditions, we obtained 18 attractors, S1 to S18, tabulated in Fig. 3. Among them, three sets are of interest: the proliferating-like attractors with up-regulated proliferating factors such as E2F, Cyclin D/E, Myc; the differentiated-like ones with low E2F and up-regulated inflammatory cytokines; and the ones with apoptotic signatures.

We identified attractor S1 as APL-like phenotypic state for the following reasons. We obtained microarray data from Gene Expression Omnibus (GEO) for retinoic acid treated APL cell lines NB4^16^ and HL-60^17^. In these experiments, after retinoic acid treatment, expressions of molecules and pathways responsible for cell proliferation, such as Myc, CCNE1, 2 (Cyclin E1, E2), E2F1 were reduced. Simultaneously, NR2F2 was decreased; CEBPB increased; CSF3R (G-CSFR) increased. A consistent assignment is attractor S1 in Fig. 3 as APL-like phenotypic state.

Attractors S12 and S14 in Fig. 3 are differentiated state-like, containing features of mature neutrophil for its elevated IL-8, INF-*γ* and Stat1 activity^18^. Attractor S4 may be identified as a normal neutrophil progenitor characterized by up-regulated Runx1, Pu.1, G-CSFR and high proliferation factors^18^. At normal myeloblast stage, the cell is relatively undifferentiated and proliferative^18^. APL-like attractor S1 has reduced Pu.1, consistent with experimental data^4^. Key factors of S4 and S1 are identified in Fig. 4(a). The network also contains other attractors, such as S3 and S5, which seem to be hybrids between the APL and normal progenitor like-states, shown in Fig. 3. We found that these attractors became intermediate steps in calculated induced differentiation of APL or leukemogenesis from normal progenitor-like attractor to APL-like S1, shown in Fig. 5(a). These hybrids-like attractors also suggest that APL pathogenesis is multi-stage, multi-factorial, and may be caused by aberrations in multiple cellular pathways.

Attractor S7 is an IL-3 responsive, Notch active attractor. It is inducible from progenitor-like attractor S4 via TGF-*β* up-regulation (Fig. 5(b)). The attractor S7 differs from terminally differentiated-like attractor S12 as well as progenitor-like S4. Notch pathway is a multiple functional regulator in hematopoiesis. It was found to be essential during early T-lineage development, blocking the differentiation of lymphoid progenitor cells into the B cell lineage and inducing differentiation into the T cell lineage^19^. It is required for *in vitro* but not *in vivo* maintenance of human hematopoietic stem cells and to delay the appearance of multipotent progenitors^20^. In myeloid cells, Notch signaling is stage dependent^21^. In granulopoiesis, Notch signaling was found to promote entry into granulopoiesis, and inhibits postmitotic differentiation^22^, consistent with our calculation that a possible differentiation path involves S7 as an intermediate step (Fig. 5(a)). In microarray data, retinoic acids induce Notch signaling in both NB4^16^ and HL-60 cell lines^17^. S12–S15 are differentiated-like attractors that differ in dependence on growth factors and cytokines. Hematopoiesis is a multiple stage, multiple organ process from bone marrow to bloodstream to tissue. Presumably, multiple differentiated states depending on different growth factors and cytokines for survival might be necessary^23^.

### Sub-network for individual attractor maintenance: Identification of key factors in APL development

After identifying APL and normal progenitor-like attractors supported by the endogenous network, we studied the molecular mechanism for their formation. The mathematical model allows identifying active molecules and interactions responsible for each attractor as well as the relationship among different attractors. In Fig. 4(a), the active nodes and edges for APL-like and the normal progenitor-like attractors are overlaid. There are a number of overlapped ones such as those involved in proliferation and cell survival. The differential clusters of molecules and pathways in APL-like state are SHH, NR2F2, Stat5, Sox4 while in normal progenitor-like state are BMPs, G-CSFR, Pu.1, and Runx1. Since SHH and NR2F2 are important inducer of angiogenesis^24^,^25^, it is possible that angiogenesis also plays an important role in leukemia. This abnormal cluster of molecules and pathways in APL-like attractor is suppressed by hematopoietic lineage specific factor Runx1 and by BMPs in normal progenitor-like attractor S4. The requirement of uncompromised BMP signaling from the modeling results is consistent with the important role of osteoblast in hematopoietic stem cell niche formation^23^. The differentiated states mostly do not overlap with the proliferating ones, as shown in Fig. 3. Differentiated states have up-regulated retinoic acid activities, RARs, in the modeling results.

### Switching between attractors: implication in leukemogenesis and interpretation of therapeutical studies

We have shown association between attractors in the network with physiological and pathological phenotypes. These attractors are robust dynamical structure determined by the collective molecular interactions, not easily switched to one another by perturbations. Combination of changes in molecular activity or expression most likely to switch out of APL-like attractor might be relevant to therapeutical targets. In order to obtain detailed information on induced switching among relevant states, we systematically perturbed APL-like attractor by up-regulating or down-regulating different nodes. We found that APL-like attractor is induced to become attractor S4, the normal progenitor-like state, with down-regulated SHH, up-regulated Runx1 (or Runx2) and BMP simultaneously, shown in Fig. 4(b). Therapeutically, BMP induction may be possible by vitamin D derivatives^26^. Runx1/2 are both BMP downstream targets (see Fig. 2). Vitamin D is also able to inhibit SHH pathway^27^. It is known that APL cell line is able to differentiate by vitamin D induction^28^, consistent with our calculation. We also found that APL-like attractor is inducible to normal progenitor-like state indirectly via attractor S5 by down-regulated VEGF and up-regulated RARs (Fig. 5(b)). Since ATO both inhibits SHH signaling^29^,^30^,^31^ as well as inhibits VEGF^32^, it might help to induce APL-like attractor to normal progenitor-like one along multiple paths. We also calculated an induced switch from normal progenitor-like attractor S4 to differentiated neutrophil-like attractor S12 via an intermediate attractor S7. We found that TGF-*β* and RARs are inducers for normal neutrophil differentiation from normal progenitor-like attractor S4 (Fig. 5(b)).

As a structure of dynamical network, attractors are linked via saddle and other unstable fixed points, and are listed in Supplementary Information. When perturbed, saddle and other unstable fixed points flow to different attractors. The saddle and other unstable fixed points might have dynamical interpretation as the easiest passes connecting attractors under perturbation. The attractors are connected though multiple paths as shown in Fig. 5(a). Such a dynamical topology demonstrates that APL-like state is inducible from multiple attractor associated phenotypes, suggesting heterogeneous leukemogenesis. A more detailed exposition on cancer heterogeneity within the current framework was in ref. 33. Of particular interest is a switching from normal progenitor-like attractor to APL-like one, S4 to S1. Since S4 and S1 are not directly connected by saddles, we looked for S4 to S1 via S3 or S5. The saddles along these paths contain changes in lost BMP signaling and gained NR2F2, SHH signaling, consistent with the analysis for attractors.

### Comparison with microarray profile

This network is constructed from molecular interactions among each pair of nodes chosen for their involvement in ATRA treatment, normal neutrophil development and APL pathology. If indeed it plays the role of the core endogenous network, it should reproduce major experimental findings for APL as well as neutrophil differentiation, such as molecular profile for major phenotypes. In Fig. 6, we compared three sets of microarray data GSE19203^16^, GSE5007^17^, and GSE42519^34^ with the calculated results from our network in the upper panel of Fig. 6(a) except for nodes representing phosphorylation levels, which are unavailable from microarray data. We included BMP, TGF-*β*, NR2F2, SHH pathways and their target genes in the lower panel. Retinoic acid direct target genes and Pu.1 target genes are also included. Since NR2F2 itself is a retinoic acid target, its pathway is excluded from retinoic acid direct target genes. The references for their annotation are given in Supplementary File 1. Both microarray data and calculation show that the gross feature of normal neutrophil differentiation is similar to APL differentiation under ATRA treatment. The ATRA treated APL cell lines also demonstrated increased apoptosis markers Caspase 3/8, showing resistance to apoptosis without treatment. Overall, microarray data is consistent with the interpretation that APL cells are trapped in a state described by attractor S1. The microarray profile of BMP, TGF-*β*, NR2F2, SHH pathways and their switching behavior indicate that they are involved in neutrophil differentiation and ATRA treatment, in agreement with network modeling. The two cell lines, HL-60 and NB4, show some varied gene expression in retinoic acid target genes, but not in BMP, TGF-*β*, NR2F2, and SHH pathways, indicating retinoic acid pathway might already be partially active in HL-60 cell lines. Nevertheless, a full induction to differentiation requires the switching of BMP, TGF-*β*, NR2F2, and SHH pathways, as both calculation and analysis of microarray profile suggested.

Figure 6(b) shows genes differentially expressed in neutrophil development and ATRA treatment of APL cells compared with calculation results, as a test of whether or not our model can explain gene profiles considered important in previous independent works. The list of genes is obtained from Figs 3 and 5 in ref. 18. The nodes of the network and their targets directly represent most of the molecules in the cell cycle module. Details used to determine targets are given in Supplementary File 1. In the differentiation module (receptors and ligands), the network only gave predictions to neutrophil specific genes. The predictions are consistent with the observation for these genes. The differentiated neutrophil cells express receptors and ligands both specific to neutrophil as well as common to other leukocytes such as IL18R and IL4R. The receptors and ligands downstream of Stat4/6 pathways are not included in our network. These pathways are considered critical for the development of Th1/Th2 cells from naive CD4^+^ T cells^35^,^36^. They may be included when expanding the network in the future especially for the purpose of modeling lymphocytic leukemia.

## Discussion

In the process of carcinogenesis, hundreds of molecules and molecular pathways are affected^37^. The transition from normality to malignancy can be triggered by both genetic changes that occur within the cell (mutations) as well as external influences such as infection. Although genetically APL is associated with a single translocation, we found that the molecular modules necessary for the interaction network to reproduce the most important phenotypical profiles of APL and remission under treatment are fairly extensive. In particular, molecular pathways considered specific for embryonic organogenesis and mesenchymal development are found to play critical roles in the network. In addition, the model suggests a basic structure of cancer: the normal states and cancer states mutually suppress each other, both in “wiring” and in dynamical cooperation.

Another interesting question is the origin of this APL-like attractor in the molecular interaction network, or equivalently, the mechanism preventing evolution to get rid of this attractor altogether. We found that the APL-like attractor formation may be linked to angiogenesis. Hematopoiesis is a conserved biological process in vertebrates. Given the importance of SHH and NR2F2 in angiogenesis^24^,^25^, APL might be interpreted as a result of hematopoietic to mesenchymal transition. Developmentally, endothelial and mesenchymal cell lineages are both likely to be the origin of hematopoietic stem cells^38^. Thus both endothelial and mesenchymal to hematopoietic transitions are possibly required developmentally. It is convincible that a state similar to APL might have function early in embryonic development. In other words, APL might be interpreted as a faulty reversion of hematopoietic phenotype to an endothelial/mesenchymal one.

Biologically, the most important open question on APL mechanism concerns successful APL treatment in contrast with AMLs, which do not respond to the same differentiation therapy. In microarray data^16^,^17^, ATRA induces down-regulation of NR2F2 and up-regulation of VEGFR1/Flt1, a negative regulator of angiogenesis shown to be regulated by NR2F2^25^. ATO is a potent inhibitor of SHH pathway^29^,^30^,^31^, shown to modulate SHH by inhibiting SHH signaling at the Gli protein level^29^,^30^,^31^. It is possible that in addition to targeting PML/RAR*α*^39^, ATO also helps to control SHH signaling. Therefore, ATRA-based therapy targets two pathways of angiogenesis. Such an intuitive picture might be underlying the difference between APL and other AMLs, for NR2F2 is often silenced in leukemias^25^. Notch pathway, active in mesenchymal stem cell, is up-regulated in many cancers including certain leukemias^40^, possibly defining another mesenchymal phenotype of leukemias. Finally, this model contains essential modules for myeloid lineage development and pathology to be carried over to other AMLs with modification and extension.

## Methods

**Figure 1:**
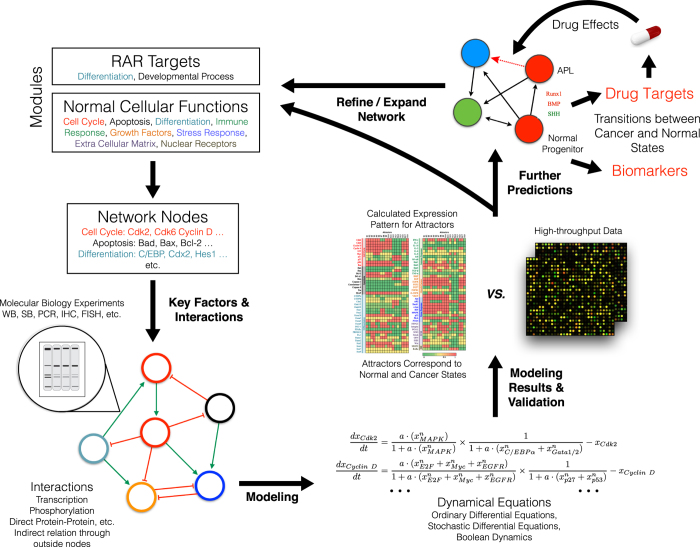
Outline of endogenous molecular-cellular network construction and modeling of APL. *Network construction*: we start with summarizing retinoic acid targets as well as normal cellular functions at modular level. We choose key nodes in each module with reference to previous cancer models^42^,^43^ to construct a minimal core network presenting basic cellular functions. Endogenous agents (genes, molecules, or pathways) specific for myeloid development, such as transcription factors Pu.1, C/EBP*β*, Runx1, and signaling pathways Stat1/5 are included in the network. The interactions between agents are collected from a large amount of literature supported by solid molecular biology experiments. Feedbacks related to embryonic development, especially those regulated by retinoic acid are included. *Analysis of the network*: we use dynamical equations to calculate attractors formed by the network structure. We run random parameter tests to demonstrate the results are robust. Comparison with high-throughput data further validates our modeling. *Mechanism*: In this context, APL as well as normal phenotypes correspond to attractors of the network dynamics. We find that SHH, BMP, and VEGF pathways are indispensable for the network to form the APL-like attractor. Leukemogenesis may be understood as a transition from normal states to APL state due to an accumulation of internal fluctuations or external perturbations. Induced switching from APL-like attractor thus represents differentiation therapy. *Further predictions*: we make a series of “dry experiments” on the endogenous network. We predict drug targets based on systematic perturbations of APL-like state to a normal state. Biomarkers can be recognized as differentially expressed agents or their downstream genes from the calculated molecular profiles of the attractors. Drug effects may also be tested through the overall influence of a drug on targeted agents.

**Figure 2:**
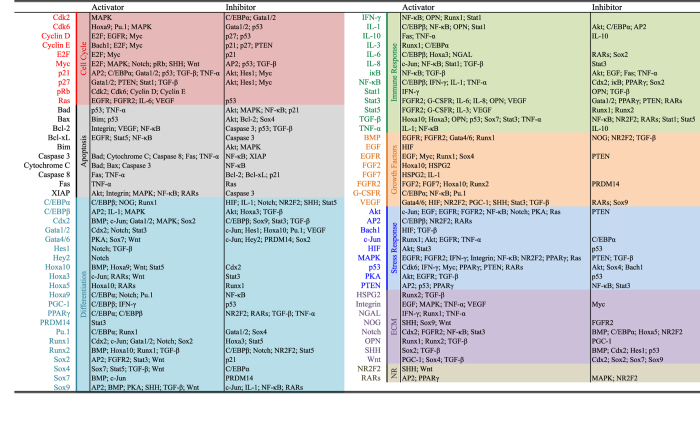
Molecular interactions in the APL network. Molecules and pathways are grouped according to their roles in cell cycle, apoptosis, growth factors, differentiation, immune response and stress response. NR: nuclear receptors. ECM: molecules and pathways interact with extracellular matrix. Detailed description and references are shown in Supplementary Information.

Network construction and dynamical modeling was performed from the viewpoint of endogenous molecular-cellular network hypothesis on cancer^14^. The hypothesis suggests the existence of a minimal set of core molecular-cellular agents interacting with each other through signaling transduction and transcriptional regulation to form an endogenous molecular-cellular network. The dynamical modeling of the endogenous network recapitulates the hallmarks of cancer^41^ quantitatively. Endogenous network models have been constructed and verified by studies on various cancers including hepatic^42^, prostate^43^, and gastric^33^. In addition, the framework is able to predict gain or loss of functions for genetic mutations, such as in the hepatocellular carcinoma^44^. The general method was summarized in Fig. 1.

### Network construction

The construction of network followed an incremental procedure aimed at to capture the essential features of APL leukemogenesis as well as basic cell functions while keeping the network minimal. Retinoic acids are important hormone-like molecules which regulate a wide range of embryonic development processes such as hematopoiesis, central nervous system and early pancreatic bud formation. Under physiological condition, the molecular feedbacks from transcriptional regulations outside hematopoietic lineages need not to be considered. However, it is unclear to what extend these feedbacks participate in leukemogenesis. The network construction started with standard and generic cellular function modules such as cell cycle, apoptosis, stress response, and then myeloid differentiation was added. Next, feedbacks related to embryonic development in endothelial and mesenchymal cell lineages^38^ regulated by retinoic acid were added. We found that SHH, BMP, VEGF pathways were indispensable for the network as well.

Note that interactions included in the network are from studies under both physiological and pathological conditions. We assume that most of the molecular regulations are basically intact for the core functions of cells such as cell cycle, Notch pathway etc. In this APL network, the interactions are mostly of direct biochemical type, such as phosphorylation, that may be invariant under both conditions. Nevertheless, there are may be differences in these two conditions. Such differences may be reflected by perturbations on the dynamical equations used. As demonstrated in the supplementary information, the stable states obtained from modeling are robust under perturbations. The emergence and robustness of both cancer and normal states from one endogenous network, therefore, is a direct support of our assumption.

The nodes presenting the generic features of cellular functions were mostly taken from previous works^42^,^43^, which represent simplified core network constructed with the pathways and modules essential to physiological functions: cell cycle, apoptosis, growth factors, differentiation, immune response, and stress response. Several genes involved in myeloid differentiation including transcription factor Pu.1^45^,^46^ and CCAAT/enhancer-binding proteins (C/EBPs) such as C/EBP*β*^47^ were added in the network. Since STAT5b-RAR*α* fusion protein is also identified in APL, we included Stat5 and Stat3 pathways as well as RARs^48^. For the same reason, Runx1 was included because of RUNX1-MDS1-EVI1 fusion protein^49^.

Genes and regulatory molecules affected by ATRA through RARs were selected. Besides Hox family, which are direct retinoic acids targets and participate in vertebrate embryonic development^50^, other molecules and molecular pathways added are mostly indirectly regulated by retinoic acids. Finally, to keep the network within a manageable size, for parallel or redundant processes, only representative molecules were included.

### Network modeling

A challenge of analyzing endogenous networks is a lack of detailed biological knowledge for modeling the complex interactions between agents, e.g., the absent of *in vivo* parameters. A semi-quantitative description was thus employed for the core network consisting of a set of nonlinear and coupled differential equations depicting “macroscopic” activation or inhibition between agents, as a refinement of more primitive but useful descriptions such as the Boolean dynamics (Boolean network)^51^. More dynamical information than Boolean network was obtained by our method such as saddle points as critical points of transitions between connected attractors. This type of modeling can further be improved when more biological knowledge is available. For validation: (a) we used two independent methods to obtain attractors from differential equations under different parameter settings (Supplementary Table II and III); (b) we made a series of random parameter tests (see Supplementary Figs 1 and 2) and the results are almost invariant; (c) we used alternative forms of equations and the major functional attractors discussed are reproduced (Supplementary Table IV); (d) we calculated Boolean dynamics and found the result supports all attractors obtained from differential equations (see Supplementary Table V). The comparison of modeling generated molecular profiles with microarray data is reported in Results.

The dynamical equation for the concentration (activity) of an agent *x*_*i*_ under the influence of other agents in the network may be written as the form of chemical rate equation including generation rate *f* and degradation rate *g*:


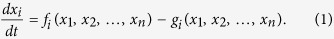


Since generally the expression of *f* and *g* are unknown, we consider a coarse-grained model to catch the “macroscopic” feature: (a) we consider all agents are exponentially self-degrading with *g*_*i*_ = *x*_*i*_; and (b) we use the following defined *f* with an upper bound value 1 for normalizing all concentrations (that are assumed to have a finite maximum value) to vary between [0, 1]; (c) we employ the commonly used Hill type function^52^,^53^ for signaling transduction and gene regulation to implement *f*. If an agent *x*_2_ activates agent *x*_1_, function *f*_1_(*x*_2_) is assigned to the form


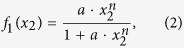


where *n* is the Hill coefficient. If there is an inhibition on *x*_1_ from *x*_2_


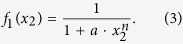


If there are multiple activation *x*_2_, *x*_3_ and inhibition *x*_4_, *x*_5_ to *x*_1_, we have


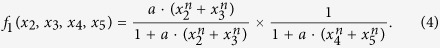


A full list of 81 equations can be viewed in Supplementary Information. Note that under a recent framework for stochastic differential equations^54^,^55^, the description including stochasticity by adding noise term to Eq. (1) generates consistent results with the deterministic counterpart. A potential landscape corresponds to steady state distribution can be obtained. Stochastic transitions and stability of attractors are able to be quantitatively discussed.

**Figure 3:**
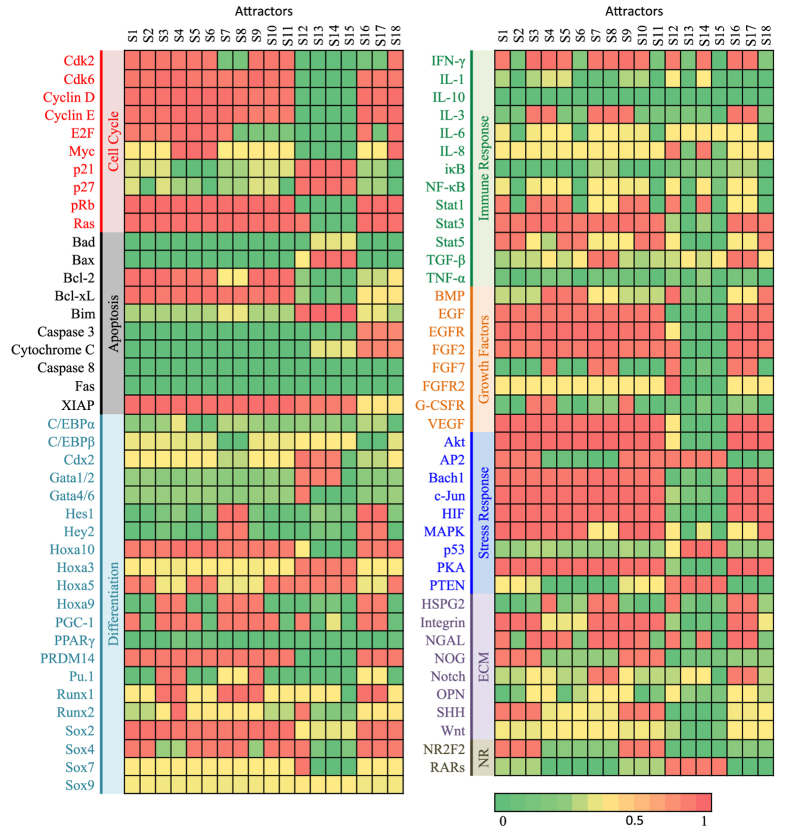
Molecular profiles of the attractors in the dynamical model of the network given in Fig. 2. The corresponding equations are listed in Supplementary Information. S1–S7 represent proliferating phenotypes. S1 is APL-like. S4 is normal progenitor-like. S2 is similar to S1. S3 is an intermediate phenotype between S2 and S4. S5 is an intermediate phenotype between S1 and S4. S7 is an intermediate phenotype between S4 and a differentiated state S12. S8–S11 are non-proliferating, which are otherwise similar to S1, S2, S3 and S7. S12–S15 are differentiated phenotypes. S16–S18 are apoptotic.

**Figure 4:**
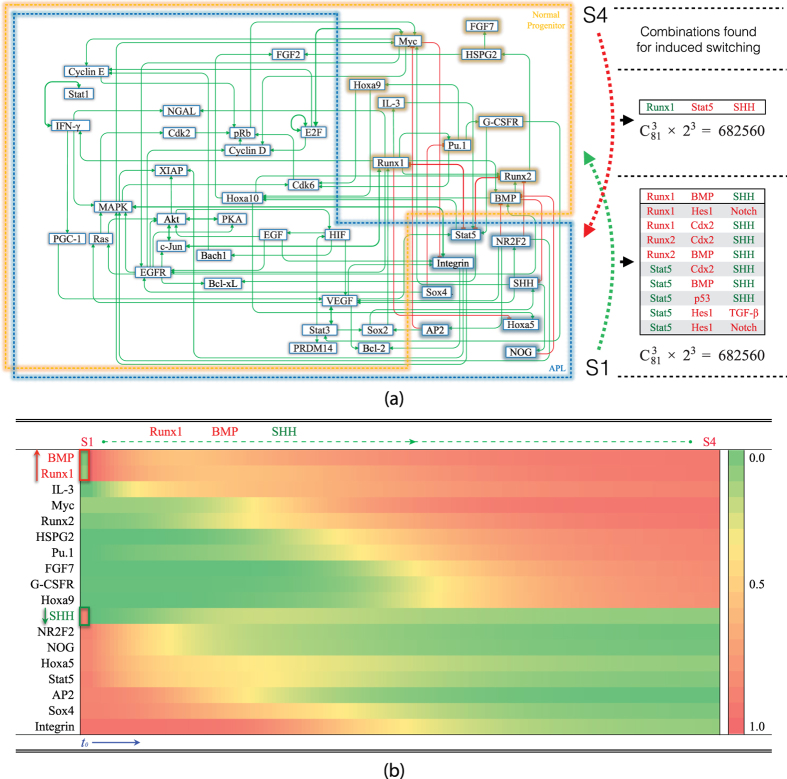
Effective network of APL-like and normal progenitor-like attractors and induced switching between them. (**a**) Effective sub-networks for APL-like and normal progenitor-like attractors, S1 and S4 in Fig. 3. The effective sub-networks are obtained by selecting active nodes (>0.5) in S1 and S4 and their molecular interactions. Within blue/orange dotted lines are the nodes active in S1/S4. Solid green/red lines represent activation/inhibition. Double arrowed green/red lines denote mutual activation/inhibition. These two attractors have both common active nodes and distinct ones. Inhibitory interactions dominate the differential ones in these two attractors such as those among BMP, Runx1, Stat5 and SHH. (**b**) Induced switching from APL-like attractor S1 to normal progenitor-like attractor. Starting from APL-like attractor S1, at time *t* = *t*_0_, values of three nodes are changed to: SHH = 0, Runx1 = 1, BMP = 1. Trajectory of the time course shows that after such an induction, the state of the network will switch from S1 to S4. The trajectory of differentially expressed nodes during the switching process is shown in this graph.

**Figure 5:**
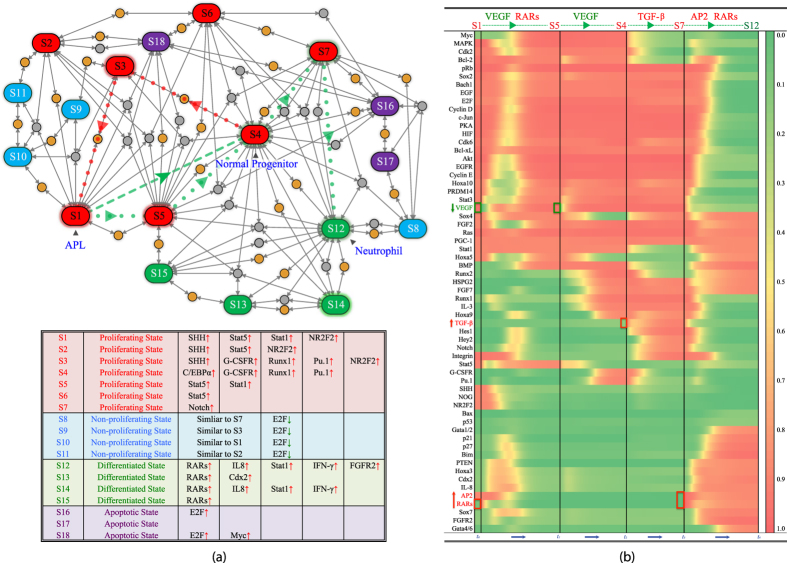
Attractors connected by saddle/unstable fixed points on the potential landscape and switches between attractors. (**a**) Attractors linked by saddle/unstable fixed points. In addition to attractors, the dynamical model also contains saddles and other unstable fixed points. S1–S18 are attractors, as shown in Fig. 3 and briefly summarized in a table below. The saddle points are yellow dots and other unstable fixed points are grey dots. When perturbed, saddle/unstable fixed points flow to attractors. The flows from saddle/unstable fixed points to attractors are represented by grey arrowed lines. The red dotted lines demonstrate a possible route (leukemogenesis) to APL-like attractor S1 from normal progenitor-like attractor S4 through saddle points and S3. An induced switch from S1 to S4 as discussed in Fig. 4(b) is drawn with a green dashed line. The green dotted lines demonstrate an alternative route to induce APL-like attractor S1 to normal progenitor-like attractor S4, and then continue to induce to differentiated-like attractors. Switching from S1 to S4 via S5 is induced by suppressing VEGF and activating RARs. Differentiation from S4 is triggered by TGF-*β* and RARs. The details are given in the right panel (**b**). (**b**) Induced switching from APL-like attractor S1 to differentiated state-like attractor S12. In the differentiation course, suppressing VEGF and activating RARs are among the key actions. Starting from APL-like attractor S1, at time *t* = *t*_0_, values of two nodes are changed to: RARs = 1, VEGF = 0. Afterwards, the state of the network switches from S1 to S5. The trajectory during the switching process is shown in the figure, from *t* = *t*_0_ to *t* = *t*_1_. VEGF returns to 1. From S5, at time *t* = *t*_1_, value of VEGF is again set to zero. After that, the network switches from S5 to S4. The trajectory during the switching process is shown in the figure, from *t* = *t*_1_ to *t* = *t*_2_. At time *t* = *t*_2_, from S4, after TGF-*β* is activated, TGF-*β* = 1, the the state of the network switches from S4 to S7. The trajectory of this process is shown from *t* = *t*_2_ to *t* = *t*_3_. At time *t* = *t*_3_, from S7, two nodes are activated: RARs = 1, AP2 = 1. The switch from S4 to S12 is shown from *t* = *t*_3_ to *t* = *t*_4_.

**Figure 6:**
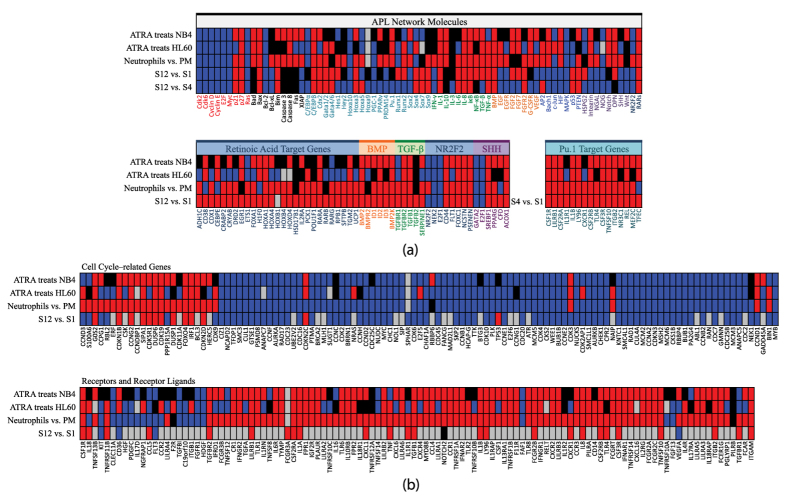
Comparison of modeling results with microarray profiles. (GSE19203^16^, GSE5007^17^, GSE42519^34^) of normal neutrophil differentiation process (PM is for promyelocyte^34^) and differentiation of APL cell lines treated with ATRA (all-trans-retinoic acid). (**a**) Microarray profiles of gene expression corresponding to the nodes in the APL network are shown in the upper panel and compared with calculation. BMP, TGF-*β*, NR2F2, SHH pathways and their target genes, retinoic acid direct target genes and Pu.1 target genes are shown in the lower panel. The references and annotations are given in Supplementary File 1. The gross feature of normal neutrophil differentiation is similar to APL differentiation under ATRA treatment. (**b**) Profiles of genes differentially expressed in neutrophil development and ATRA treatment of APL cell lines compare with calculation results. The list of genes is obtained from Figs 3 and 5 in ref. 18. Most of molecules in the cell cycle related gene list are directly represented by the nodes of the network and their effectors. For expression of receptors and ligands, the network gave predictions to neutrophil specific genes (others are left grey). The differentiated neutrophils and cell lines express receptors and ligands both specific to neutrophil as well as common to other leukocytes. Colored grids in the figure: Red/Blue denotes the value of the former minus the later/the later minus the former is higher than a threshold. Black means the difference is not significant (within the threshold). Grey is for gene symbols not found in a certain dataset.

In this APL model, we obtained 18 attractors, 32 saddle points, as well as 25 other unstable fixed points shown in Fig. 3, Supplementary Table II, VI and VII separately. Based on a large number of random searches using two independent methods, our results may cover the major attractors that are biologically stable (with a relatively large basin of attraction) in the state space. In real cases, the Hill coefficient *n* may vary from interaction to interaction, we did random parameter tests that allow each interaction (totally 416) having independent *n*_*i*_ and *a*_*i*_, *i* = 1, …, 416 (*n*_*i*_ and *a*_*i*_ can be dependent or independent). The statistics on the recurrence rate of each attractor (obtained using the above equations with fixed *n* and *a*) demonstrates the robustness of our approach (in Supplementary Fig. 2): almost in all cases, the 18 attractors are reproduced. Therefore, our modeling may indeed capture the “macroscopic” features of the core endogenous network constructed that are invariant regardless of the biological details of interactions between agents.

## Additional Information

**How to cite this article**: Yuan, R. *et al.* From molecular interaction to acute promyelocytic leukemia: Calculating leukemogenesis and remission from endogenous molecular-cellular network. *Sci. Rep.* **6**, 24307; doi: 10.1038/srep24307 (2016).

## Supplementary Material

Supplementary Information
