# Supplementary material for: From molecular interaction to acute promyelocytic leukemia: Calculating leukemogenesis and remission from endogenous molecular-cellular network
Source: Sci Rep. 2016 Apr 21;6:24307. doi: 10.1038/srep24307 (PMC4838884; doi:10.1038/srep24307)
Supplement: Supplementary Information [file srep24307-s1.pdf]

Supplementary Materials for "From molecular interaction to acute promyelocytic leukemia: Calculating leukemogenesis and remission from endogenous molecular-cellular network"

Ruoshi Yuan,[1,2] Xiaomei Zhu,[3] Jerald P. Radich,[4] and Ping Ao[1]

[1]*Key Laboratory of Systems Biomedicine, Ministry of Education,*
Shanghai Center for Systems Biomedicine,
Shanghai Jiao Tong University, Shanghai, 200240, China

[2]*School of Biomedical Engineering, Shanghai Jiao Tong University, Shanghai, 200240, China*

[3]*GeneMath, 5525 27th Ave. N.E., Seattle, WA 98105, USA*

[4]*Fred Hutchinson Cancer Research Center,*
1100 Fairview Avenue N., Seattle, WA 98109, USA

(Dated: February 13, 2016)

CONTENTS

I. Network Details and References 3

References 6

II. Modeling Network Dynamics 26

A. Equations 26

B. A Complete List of Equations Used in Simulating Network Dynamics 26

C. Attractor, Saddle Points, and Other Unstable Points Obtained from the Endogenous Network Dynamics 30

D. Random Parameter Tests 30

E. Stability under Different Equation Forms 31

1. Alternative equation (a) 31

2. Alternative equation (b) 32

F. Comparison with Boolean Dynamics 32

G. Induced Transitions 33

References 33

III. Supplementary Tables and Figures 34

I. NETWORK DETAILS AND REFERENCES

Supplementary Table I: Molecular interactions with references.

	Activated By	Inhibited By	Notes
	Cell Cycle		
Cdk2	MAPK [1]	C/EBPα [2], Gata1/2 [3]	
Cdk6	Hoxa9 [4], MAPK [5], Pu.1 [6]	Gata1/2 [3], p53 [7]	
Cyclin D	E2F [8], EGFR [9], Myc [8]	p27 [8], p53 [8]	
Cyclin E	Bach1 [10], E2F [8], Myc [11]	PTEN [8], p21 [8, 12], p27 [8, 12]	
E2F	E2F [8], Myc [8]	p21 [12]	
Myc	E2F [13], MAPK [13, 14], Notch [15], SHH [16], Wnt [17], pRb [13]	AP2 [18], TGF-β [19, 20], p53 [13]	
p21	AP2 [21], C/EBPα [22], Gata1/2 [23], TGF-β [20], TNF-α [24], p53 [13, 24]	Akt [12], Hes1 [25], Myc [24, 26]	
p27	Gata1/2 [27], PTEN [28], Stat1 [29], TGF-β [30]	Akt [28], Hes1 [31], Myc [13]	
pRb	Cdk2 [8, 32], Cdk6 [8, 32], Cyclin D [8, 32], Cyclin E [8, 32]		Retinoblastoma protein phosphorylation
Ras	EGFR [33], FGFR2 [34], IL-6 [35, 36], VEGF [37, 38]	p53 [39]	
	Apoptosis		
Bad	TNF-α [40], p53 [41]	Akt [42], MAPK [42], NF-κB [43], p21 [44]	
Bax	Bim [45], p53 [46, 47]	Akt [48], Bcl-2 [49], Sox4 [50]	
Bcl-2	Integrin [51], NF-κB [52], VEGF [53]	Caspase 3 [53], TGF-β [54], p53 [55]	
Bcl-xL	EGFR [56], NF-κB [57], Stat5 [58]	Caspase 3 [59]	
Bim		Akt [42], MAPK [42]	
Caspase 3	Bad [60, 61], Caspase 8 [47, 53], Cytochrome C [47, 53, 62], Fas [63], TNF-α [64]	NF-κB [65], XIAP [47, 53, 66, 67]	
Caspase 8	Fas [47, 53, 62], TNF-α [47, 53, 62]	NF-κB [68]	
Cytochrome C	Bad [53], Bax [53], Caspase 3 [47, 53, 66, 69]	Bcl-2 [70, 71], Bcl-xL [70, 72], p21 [73, 74]	Cytochrome C, Apaf-1, and Caspase 9 complex
Fas	TNF-α [75]	Ras [76]	
XIAP	Akt [67], Integrin [77], MAPK [78], NF-κB [69, 79], RARs [80]	Caspase 3 [47, 53, 55, 66, 67, 69]	X-linked inhibitor of apoptosis
	Differentiation		
C/EBPα	C/EBPβ [81], NOG [82], Runx1 [83]	HIF [84], IL-1 [85], NR2F2 [86], Notch [87], SHH [88], Stat5 [89]	

Continued on next page

Supplementary Table I: Continued from previous page

	Activated By	Inhibited By	Notes
C/EBPβ	AP2 [90], IL-1 [85], MAPK [91]	Akt [92], Hoxa3 [93], TGF-β [94]	
Cdx2	BMP [95], Gata1/2 [96, 97], MAPK [98], Sox2 [99], c-Jun [100]	*C/EBPβ [100, 101], Sox9 [102], Stat3 [103], TGF-β [104]	*C/EBPβ downregulate Cdx2 by c-Fos upregulation
Gata1/2	Cdx2 [105], Notch [106], Stat3 [107]	Hes1 [108], Hoxa10 [109], Pu.1 [110, 111], VEGF [112], c-Jun [113]	
Gata4/6	PKA [114], Sox7 [115], Wnt [116]	Hey2 [117], PRDM14 [118], Sox2 [99, 115, 119], c-Jun [120]	
Hes1	Notch [108], TGF-β [121]		
Hey2	Notch [122]		
Hoxa10	BMP [123], Hoxa9 [124], Stat5 [125, 126], Wnt [127]	Cdx2 [128]	
Hoxa3	RARs [129, 130], Wnt [131], c-Jun [132]	Stat3 [133]	
Hoxa5	Hoxa10 [109], RARs [130]	Runx1 [134]	
Hoxa9	C/EBPα [4, 135], Notch [136], Pu.1 [4]	NF-κB [137]	
PGC-1	C/EBPβ [138], IFN-γ [139]	p53 [140]	
PPARγ	C/EBPα [141], C/EBPβ [142]	NR2F2 [86], RARs [143], TGF-β [143], TNF-α [144]	
PRDM14	Stat3 [145]		
Pu.1	C/EBPα [146], Runx1 [147, 148]	Gata1/2 [110, 111], Sox4 [149]	
Runx1	Cdx2 [105], Gata1/2 [150], Notch [151], Sox2 [152], c-Jun [153]	Hoxa3 [93, 154], Stat5 [155]	
Runx2	BMP [20], Hoxa10 [123], Runx1 [156], TGF-β [157, 158]	C/EBPβ [159], NR2F2 [160], Notch [161], Stat5 [155]	
Sox2	AP2 [162], FGFR2 [163], Stat3 [99], Wnt [164]	p21 [165]	
Sox4	Sox7 [166], Stat5 [167], TGF-β [168, 169], Wnt [166]	C/EBPα [170]	
Sox7	BMP [171], c-Jun [172]	PRDM14 [173]	
Sox9	AP2 [174], BMP [158], PKA [175], SHH [176], TGF-β [177], Wnt [102, 178]	IL-1 [179], NF-κB [179, 180], RARs [180, 181], c-Jun [182]	
		Immune Response	
IFN-γ	NF-κB [183], OPN [184], Runx1 [185], Stat1 [186]		
IL-1	C/EBPβ [187], NF-κB [188], OPN [189], Stat1 [190]	AP2 [191], Akt [192], C/EBPα [193]	Interleukin 1
IL-10	Fas [188], TNF-α [188]	IL-10 [188]	Interleukin 10
IL-3	C/EBPα [194], Runx1 [195–197]		Interleukin 3
IL-6	C/EBPβ [198], Hoxa3 [199], NGAL [200]	RARs [201], Sox2 [202]	Interleukin 6
IL-8	NF-κB [203, 204], Stat1 [205], TGF-β [206], c-Jun [204, 207, 208]	Stat3 [208]	Interleukin 8

Continued on next page

Supplementary Table I: Continued from previous page

	Activated By	Inhibited By	Notes
iκB	NF-κB [79, 209, 210], TGF-β [211]	Akt [212], EGF [213], Fas [214], TNF-α [79, 209, 210]	
NF-κB	C/EBPβ [215], IFN-γ [216], IL-1 [79, 209, 210, 217], TNF-α [79, 209, 210]	Cdx2 [218], PPARγ [219], Sox2 [220], iκB [79, 209, 210]	
Stat1	IFN-γ [221, 222]	OPN [223], TGF-β [222]	
Stat3	FGFR2 [224], G-CSFR [225], IL-6 [226], IL-8 [227], OPN [228], VEGF [229]	Gata1/2 [230], PPARγ [231], PTEN [232, 233], RARs [234]	
Stat5	FGFR2 [235], G-CSFR [225], IL-3 [58], VEGF [236]	Runx1 [155], Runx2 [155]	
TGF-β	Hoxa10 [237], Hoxa3 [93], OPN [238], Sox7 [239], Stat3 [240], TNF-α [20, 241], p53 [242, 243]	NF-κB [20], NR2F2 [244], RARs [245], Stat1 [20, 246], Stat5 [247]	Transforming growth factor beta
TNF-α	IL-1 [79, 209, 210], NF-κB [79, 209, 210]	IL-10 [79, 209, 210]	
	**Growth Factors**		
BMP	EGFR [248], FGFR2 [249], Gata4/6 [250], Runx1 [251]	NOG [252], NR2F2 [253], TGF-β [254]	Bone morphogenetic proteins
EGF	HIF [255]		
EGFR	EGF [33], Myc [256], Runx1 [257], Sox4 [169]	PTEN [258]	
FGF2	HSPG2 [259], Hoxa10 [237]		Fibroblast growth factor 2
FGF7	HSPG2 [259], IL-1 [260]		Fibroblast growth factor 7
FGFR2	FGF2 [224], FGF7 [224], Hoxa10 [237], Runx2 [261]	PRDM14 [262]	Fibroblast growth factor receptor 2
G-CSFR	C/EBPα [263], NF-κB [264], Pu.1 [265]		
VEGF	Gata4/6 [266], HIF [267], NR2F2 [268], PGC-1 [269], SHH [270], Stat3 [271–273], TGF-β [274, 275]	RARs [276], Sox9 [277]	Vascular endothelial growth factor
	**Stress Response**		
Akt	EGF [278], EGFR [278], FGFR2 [224, 279], NF-κB [42, 210], Notch [280], PKA [281], Ras [282], c-Jun [283]	PTEN [284]	
AP2	C/EBPβ [285], NR2F2 [286], RARs [287]		Activating Protein 2 (AP-2)
Bach1	HIF [288], TGF-β [289]		BTB and CNC homology 1
c-Jun	Akt [290], EGFR [291], Runx1 [153], TNF-α [292]	C/EBPα [293]	
HIF	Akt [42, 294], Stat3 [273]	p53 [294]	Hypoxia-inducible factors

Continued on next page

Supplementary Table I: Continued from previous page

	Activated By	Inhibited By	Notes
MAPK	EGFR [295], FGFR2 [296], IFN-γ [297], Integrin [298], NF-κB [14], NR2F2 [299], PPARγ [300], Ras [37]	PTEN [284], TGF-β [301]	Mitogen-activated protein kinase signaling
p53	Cdk6 [302], IFN-γ [303], Myc [37], PPARγ [304], PTEN [305], RARs [304, 306]	Akt [37], Bach1 [307], Sox4 [308]	
PKA	Akt [309], EGFR [310], TGF-β [311, 312]	p53 [313]	
PTEN	AP2 [314], PPARγ [315], p53 [316]	NF-κB [317], Stat3 [232]	
	ECM (Extracellular Matrix)		
HSPG2	Runx2 [318], TGF-β [319]		
Integrin	EGF [51, 298], MAPK [320], TNF-α [298, 321], VEGF [51, 298]	Myc [322]	
NGAL	IFN-γ [323], Runx1 [324], TNF-α [323]		
NOG	SHH [325], Sox9 [326], Wnt [325]	FGFR2 [327]	Noggin
Notch	Cdx2 [328, 329], FGFR2 [330], NF-κB [331], Stat3 [332]	BMP [333], C/EBPα [334], Hoxa5 [335], NR2F2 [336]	Notch pathway
OPN	Runx1 [324], Runx2 [324], TGF-β [337]	*PGC-1 [338, 339]	Osteopontin, *indirect
SHH	Sox2 [152], TGF-β [340]	BMP [341, 342], Cdx2 [343], Hes1 [344], p53 [345]	Sonic hedgehog pathway
Wnt	PGC-1 [346], Sox4 [169], TGF-β [347]	Cdx2 [348], Sox2 [202, 349], Sox7 [350, 351], Sox9 [352]	Wnt pathway
	Nuclear Receptor (NR)		
NR2F2	SHH [353], Wnt [86]		COUP transcription factor 2
RARs	AP2 [354], PPARγ [355]	MAPK [356], NR2F2 [357]	Retinoic acid receptor signaling

[1] W. Zhang, J. C. Lee, S. Kumar, and M. Gowen, J. Bone Miner. Res. **14**, 528 (1999).

[2] G.-L. Wang, X. Shi, E. Salisbury, Y. Sun, J. H. Albrecht, R. G. Smith, and N. A. Timchenko, Mol. Cell. Biol. **26**, 2570 (2006).

[3] M. Rylski, J. J. Welch, Y.-Y. Chen, D. L. Letting, J. A. Diehl, L. A. Chodosh, G. A. Blobel, and M. J. Weiss, Mol. Cell. Biol. **23**, 5031 (2003).

[4] Y. Huang, K. Sitwala, J. Bronstein, D. Sanders, M. Dandekar, C. Collins, G. Robertson, J. MacDonald, T. Cezard, M. Bilenky, *et al.*, Blood **119**, 388 (2012).

[5] T. Moro, T. Ogasawara, H. Chikuda, T. Ikeda, N. Ogata, Z. Maruyama, T. Komori,

K. Hoshi, U.-I. Chung, K. Nakamura, *et al.*, J. Cell Physiol. **204**, 927 (2005).

[6] K. S. Choe, O. Ujhelly, S. N. Wontakal, and A. I. Skoultchi, J. Biol. Chem. **285**, 3044 (2010).

[7] L. Böhlig, M. Friedrich, and K. Engeland, Nucleic Acids Res. **39**, 440 (2011).

[8] A. W. Murray and T. Hunt, *The Cell Cycle: An Introduction* (Oxford University Press, Oxford, 1993).

[9] Y. Tao, X. Song, X. Deng, D. Xie, L. M. Lee, Y. Liu, W. Li, L. Li, L. Deng, Q. Wu, *et al.*, Exp. Cell Res. **303**, 240 (2005).

[10] H.-J. Warnatz, D. Schmidt, T. Manke, I. Piccini, M. Sultan, T. Borodina, D. Balzereit, W. Wruck, A. Soldatov, M. Vingron, *et al.*, J. Biol. Chem. **286**, 23521 (2011).

[11] A. J. Obaya, M. K. Mateyak, and J. M. Sedivy, Oncogene **18**, 2934 (1999).

[12] C. J. Sherr and J. M. Roberts, Genes Dev. **13**, 1501 (1999).

[13] C. V. Dang, Mol. Cell. Biol. **19**, 1 (1999).

[14] G. Pearson, F. Robinson, T. B. Gibson, B.-e. Xu, M. Karandikar, K. Berman, and M. H. Cobb, Endocr. Rev. **22**, 153 (2001).

[15] T. Palomero, W. K. Lim, D. T. Odom, M. L. Sulis, P. J. Real, A. Margolin, K. C. Barnes, J. O'Neil, D. Neuberg, A. P. Weng, *et al.*, Proc. Natl Acad. Sci. USA **103**, 18261 (2006).

[16] B. A. Hatton, P. S. Knoepfler, A. M. Kenney, D. H. Rowitch, I. M. de Alborán, J. M. Olson, and R. N. Eisenman, Cancer Res. **66**, 8655 (2006).

[17] S. Zhang, Y. Li, Y. Wu, K. Shi, L. Bing, and J. Hao, Anat. Rec. **295**, 2104 (2012).

[18] L. Yu, M. J. Hitchler, W. Sun, E. H. Sarsour, P. C. Goswami, A. J. Klingelhutz, and F. E. Domann, J. Oncol. **2009** (2009).

[19] J. Romero-Gallo, E. G. Sozmen, A. Chytil, W. E. Russell, R. Whitehead, W. T. Parks, M. S. Holdren, M. F. Her, S. Gautam, M. Magnuson, *et al.*, Oncogene **24**, 3028 (2005).

[20] J. Massagué, J. Seoane, and D. Wotton, Genes Dev. **19**, 2783 (2005).

[21] Y.-X. Zeng, K. Somasundaram, and W. S. El-Deiry, Nat. Genet. **15**, 78 (1997).

[22] N. A. Timchenko, T. E. Harris, M. Wilde, T. A. Bilyeu, B. L. Burgess-Beusse, M. J. Finegold, and G. J. Darlington, Mol. Cell. Biol. **17**, 7353 (1997).

[23] M. Papetti, S. N. Wontakal, T. Stopka, and A. I. Skoultchi, Cell Cycle **9**, 1972 (2010).

[24] A. L. Gartel and S. K. Radhakrishnan, Cancer Res. **65**, 3980 (2005).

[25] P. Monahan, S. Rybak, and L. T. Raetzman, Endocrinology **150**, 4386 (2009).

[26] J. Seoane, H.-V. Le, and J. Massagué, Nature **419**, 729 (2002).

[27] S. Ezoe, I. Mtsumura, M. Mizuki, A. Kawasaki, T. Machii, M. Yamamoto, T. Enver, and Y. Kanakura, Blood **98**, 759A (2001).

[28] A. R. Gottschalk, D. Basila, M. Wong, N. M. Dean, C. H. Brandts, D. Stokoe, and D. A. Haas-Kogan, Cancer Res. **61**, 2105 (2001).

[29] S. Wang, J. F. Raven, and A. E. Koromilas, Mol. Cancer Res. **8**, 798 (2010).

[30] J. Lecanda, V. Ganapathy, C. D'Aquino-Ardalan, B. Evans, C. Cadacio, A. Ayala, and L. I. Gold, Cell Cycle **8**, 742 (2009).

[31] K. Murata, M. Hattori, N. Hirai, Y. Shinozuka, H. Hirata, R. Kageyama, T. Sakai, and N. Minato, Mol. Cell. Biol. **25**, 4262 (2005).

[32] R. A. Weinberg, Cell **81**, 323 (1995).

[33] J. Mendelsohn and J. Baselga, Oncogene **19**, 6550 (2000).

[34] M. R. Ramsey, C. Wilson, B. Ory, S. M. Rothenberg, W. Faquin, A. A. Mills, L. W. Ellisen, *et al.*, J. Clin. Invest. **123**, 3525 (2013).

[35] J. R. Jackson, M. Seed, C. Kircher, D. Willoughby, and J. Winkler, FASEB J. **11**, 457 (1997).

[36] T. Cohen, D. Nahari, L. W. Cerem, G. Neufeld, and B.-Z. Levi, J. Biol. Chem. **271**, 736 (1996).

[37] A. B. Vojtek and C. J. Der, J. Biol. Chem. **273**, 19925 (1998).

[38] A. J. Dannenberg, S. M. Lippman, J. R. Mann, K. Subbaramaiah, and R. N. DuBois, J. Clin. Oncol. **23**, 254 (2005).

[39] P. S. Nelson, N. Clegg, H. Arnold, C. Ferguson, M. Bonham, J. White, L. Hood, and B. Lin, Proc. Natl Acad. Sci. USA **99**, 11890 (2002).

[40] S. Álvarez, A. Blanco, M. Fresno, and M. Á. Muñoz-Fernández, PLoS One **6**, e16100 (2011).

[41] L.-H. Chen, C.-Y. Hsu, C.-F. Weng, *et al.*, World J. Gastroenterol. **12**, 5175 (2006).

[42] J. E. Thompson and C. B. Thompson, J. Clin. Oncol. **22**, 4217 (2004).

[43] R. Cianfrocca, M. Muscolini, V. Marzano, A. Annibaldi, B. Marinari, M. Levrero, A. Costanzo, and L. Tuosto, Cell Death Differ. **15**, 354 (2008).

[44] M. Castedo, J. Perfettini, T. Roumier, and G. Kroemer, Cell Death Differ. **9**, 1287 (2002).

[45] T. Kuwana, M. R. Mackey, G. Perkins, M. H. Ellisman, M. Latterich, R. Schneiter, D. R.

Green, and D. D. Newmeyer, Cell **111**, 331 (2002).

[46] E. L. Soucie, M. G. Annis, J. Sedivy, J. Filmus, B. Leber, D. W. Andrews, and L. Z. Penn, Mol. Cell. Biol. **21**, 4725 (2001).

[47] H. Y. Chang and X. Yang, Microbiol. Mol. Biol. Rev. **64**, 821 (2000).

[48] S. J. Gardai, D. A. Hildeman, S. K. Frankel, B. B. Whitlock, S. C. Frasch, N. Borregaard, P. Marrack, D. L. Bratton, and P. M. Henson, J. Biol. Chem. **279**, 21085 (2004).

[49] A. Basu and S. Haldar, Mol. Hum. Reprod. **4**, 1099 (1998).

[50] W. Hur, H. Rhim, C. K. Jung, J. D. Kim, S. H. Bae, J. W. Jang, J. M. Yang, S.-T. Oh, D. G. Kim, H. J. Wang, *et al.*, Carcinogenesis **31**, 1298 (2010).

[51] D. G. Stupack and D. A. Cheresh, J. Cell Sci. **115**, 3729 (2002).

[52] C.-Y. Wang, D. C. Guttridge, M. W. Mayo, and A. S. Baldwin, Mol. Cell. Biol. **19**, 5923 (1999).

[53] I. Budihardjo, H. Oliver, M. Lutter, X. Luo, and X. Wang, Annu. Rev. Cell Dev. Biol. **15**, 269 (1999).

[54] M. M. Ahmed, R. A. Alcock, D. Chendil, S. Dey, A. Das, K. Venkatasubbarao, M. Mohiuddin, L. Sun, W. E. Strodel, and J. W. Freeman, J. Biol. Chem. **277**, 2234 (2002).

[55] S. B. Bratton, G. Walker, S. M. Srinivasula, X.-M. Sun, M. Butterworth, E. S. Alnemri, and G. M. Cohen, EMBO J. **20**, 998 (2001).

[56] J. Rueddel, V. E. Wennekes, W. Meissner, J. A. Werner, and R. Mandic, Anticancer Res. **30**, 4579 (2010).

[57] C. Chen, L. C. Edelstein, and C. Gélinas, Mol. Cell. Biol. **20**, 2687 (2000).

[58] S. Dumon, S. C. R. Santos, F. Debierre-Grockiego, V. Gouilleux-Gruart, L. Cocault, C. Boucheron, P. Mollat, S. Gisselbrecht, and F. Gouilleux, Oncogene **18**, 4191 (1999).

[59] J. M. Adams and S. Cory, Science **281**, 1322 (1998).

[60] J. Won, D. Y. Kim, M. La, D. Kim, G. G. Meadows, and C. O. Joe, J. Biol. Chem. **278**, 19347 (2003).

[61] J. E. Springer, R. D. Azbill, S. A. Nottingham, and S. E. Kennedy, J. Neurosci. **20**, 7246 (2000).

[62] C. Adrain and S. J. Martin, Trends Biochem. Sci. **26**, 390 (2001).

[63] T. S. Zheng, S. F. Schlosser, T. Dao, R. Hingorani, I. N. Crispe, J. L. Boyer, and R. A. Flavell, Proc. Natl Acad. Sci. USA **95**, 13618 (1998).

[64] X. Zhao, B. Bausano, B. R. Pike, J. K. Newcomb-Fernandez, K. K. Wang, E. Shohami, N. Ringger, S. DeFord, D. K. Anderson, and R. L. Hayes, J. Neurosci. Res. **64**, 121 (2001).

[65] L. He, B. Y. Kim, K. A. Kim, O. Kwon, S. O. Kim, E. Y. Bae, M. S. Lee, M. S. Kim, M. Jung, A. Moon, *et al.*, Cell. Signal. **19**, 1713 (2007).

[66] H. Zou, R. Yang, J. Hao, J. Wang, C. Sun, S. W. Fesik, J. C. Wu, K. J. Tomaselli, and R. C. Armstrong, J. Biol. Chem. **278**, 8091 (2003).

[67] Z. Liu, H. Li, M. Derouet, J. Filmus, E. C. LaCasse, R. G. Korneluk, R. S. Kerbel, and K. V. Rosen, J. Biol. Chem. **280**, 37383 (2005).

[68] M. Karin and A. Lin, Nat. Immunol. **3**, 221 (2002).

[69] Q. L. Deveraux and J. C. Reed, Genes Dev. **13**, 239 (1999).

[70] D. T. Chao and S. J. Korsmeyer, Annu. Rev. Immunol. **16**, 395 (1998).

[71] Z. Zhang, S. M. Lapolla, M. G. Annis, M. Truscott, G. J. Roberts, Y. Miao, Y. Shao, C. Tan, J. Peng, A. E. Johnson, *et al.*, J. Biol. Chem. **279**, 43920 (2004).

[72] J. E. Chipuk, M. Bhat, A. Y. Hsing, J. Ma, and D. Danielpour, J. Biol. Chem. **276**, 26614 (2001).

[73] T. Glaser, B. Wagenknecht, and M. Weller, Oncogene **20**, 4757 (2001).

[74] O. Coqueret, Trends Cell Biol. **13**, 65 (2003).

[75] K. Kimura and E. P. Gelmann, J. Biol. Chem. **275**, 8610 (2000).

[76] J. Peli, M. Schröter, C. Rudaz, M. Hahne, C. Meyer, E. Reichmann, and J. Tschopp, EMBO J. **18**, 1824 (1999).

[77] S. Dong and D. J. Tweardy, Blood **99**, 2637 (2002).

[78] M. Abkhezr, A. R. Keramati, S. N. Ostad, J. Davoodi, and M. H. Ghahremani, Mol. Biol. Rep. **37**, 2037 (2010).

[79] H. L. Pahl, Oncogene **18**, 6853 (1999).

[80] J. Wang, Y. Peng, Y. W. Sun, H. He, S. Zhu, X. An, M. Li, M. Lin, B. Zou, H. H.-X. Xia, *et al.*, Gastroenterology **130**, 747 (2006).

[81] Z. Wu, Y. Xie, N. Bucher, and S. R. Farmer, Genes Dev. **9**, 2350 (1995).

[82] A. Sawant, D. Chanda, T. Isayeva, G. Tsuladze, W. T. Garvey, and S. Ponnazhagan, J. Biol. Chem. **287**, 12241 (2012).

[83] H. Guo, O. Ma, N. A. Speck, and A. D. Friedman, Blood **119**, 4408 (2012).

[84] E. R. Anderson, M. Taylor, X. Xue, A. Martin, D. S. Moons, M. B. Omary, and Y. M.

Shah, Mol. Cell. Biol. **32**, 4068 (2012).

[85] M. R. An, C. C. Hsieh, P. D. Reisner, J. P. Rabek, S. G. Scott, D. T. Kuninger, and J. Papaconstantinou, Mol. Cell. Biol. **16**, 2295 (1996).

[86] M. Okamura, H. Kudo, K.-i. Wakabayashi, T. Tanaka, A. Nonaka, A. Uchida, S. Tsutsumi, I. Sakakibara, M. Naito, T. F. Osborne, *et al.*, Proc. Natl Acad. Sci. USA **106**, 5819 (2009).

[87] H. Yamasaki, A. Sada, T. Iwata, T. Niwa, M. Tomizawa, K. G. Xanthopoulos, T. Koike, and N. Shiojiri, Development **133**, 4233 (2006).

[88] S. Spinella-Jaegle, G. Rawadi, S. Kawai, S. Gallea, C. Faucheu, P. Mollat, B. Courtois, B. Bergaud, V. Ramez, A. M. Blanchet, *et al.*, J. Cell Sci. **114**, 2085 (2001).

[89] A. T. Wierenga, H. Schepers, M. A. Moore, E. Vellenga, and J. J. Schuringa, Blood **107**, 4326 (2006).

[90] X. Wang, H. A. Pasolli, T. Williams, and E. Fuchs, J. Cell Biol. **183**, 37 (2008).

[91] M. N. Bradley, L. Zhou, and S. T. Smale, Mol. Cell. Biol. **23**, 4841 (2003).

[92] B.-H. Park, L. Qiang, and S. R. Farmer, Mol. Cell. Biol. **24**, 8671 (2004).

[93] M. Iacovino, D. Chong, I. Szatmari, L. Hartweck, D. Rux, A. Caprioli, O. Cleaver, and M. Kyba, Nat. Cell Biol. **13**, 72 (2010).

[94] L. Choy and R. Derynck, J. Biol. Chem. **278**, 9609 (2003).

[95] R. Barros, B. Pereira, I. Duluc, M. Azevedo, N. Mendes, V. Camilo, R. Jacobs, P. Paulo, F. Santos-Silva, I. van Seuningen, *et al.*, J. Pathol. **215**, 411 (2008).

[96] P. Home, S. Ray, D. Dutta, I. Bronshteyn, M. Larson, and S. Paul, J. Biol. Chem. **284**, 28729 (2009).

[97] H. Bai, T. Sakurai, Y. Someya, T. Konno, A. Ideta, Y. Aoyagi, and K. Imakawa, J Reprod. Dev. **57**, 518 (2011).

[98] M. Murohashi, T. Nakamura, S. Tanaka, T. Ichise, N. Yoshida, T. Yamamoto, M. Shibuya, J. Schlessinger, and N. Gotoh, Stem Cells **28**, 113 (2010).

[99] K. Adachi, H. Suemori, S.-y. Yasuda, N. Nakatsuji, and E. Kawase, Genes Cells **15**, 455 (2010).

[100] O. Lorentz, A. Cadoret, I. Duluc, J. Capeau, C. Gespach, G. Cherqui, and J.-N. Freund, Oncogene **18**, 87 (1999).

[101] J. Liao, G. Piwien-Pilipuk, S. E. Ross, C. L. Hodge, L. Sealy, O. A. MacDougald, and J. Schwartz, J. Biol. Chem. **274**, 31597 (1999).

[102] P. Blache, M. Van De Wetering, I. Duluc, C. Domon, P. Berta, J.-N. Freund, H. Clevers, and P. Jay, J. Cell Biol. **166**, 37 (2004).

[103] H. Ura, M. Usuda, K. Kinoshita, C. Sun, K. Mori, T. Akagi, T. Matsuda, H. Koide, and T. Yokota, J. Biol. Chem. **283**, 9713 (2008).

[104] K. Selesniemi, M. Reedy, A. Gultice, L. J. Guilbert, and T. L. Brown, Stem Cells Dev. **14**, 697 (2005).

[105] V. P. Rawat, R. K. Humphries, and C. Buske, Blood **120**, 519 (2012).

[106] T. Borggrefe and F. Oswald, Cell. Mol. Life Sci. **66**, 1631 (2009).

[107] K. Kang, H. Jung, S. Nam, and J.-S. Lim, Immune Netw. **11**, 348 (2011).

[108] E. Ishiko, I. Matsumura, S. Ezoe, K. Gale, J. Ishiko, Y. Satoh, H. Tanaka, H. Shibayama, M. Mizuki, T. Era, *et al.*, J. Biol. Chem. **280**, 4929 (2005).

[109] M. Magnusson, A. C. Brun, N. Miyake, J. Larsson, M. Ehinger, J. M. Bjornsson, A. Wutz, M. Sigvardsson, and S. Karlsson, Blood **109**, 3687 (2007).

[110] S. N. Wontakal, X. Guo, C. Smith, T. MacCarthy, E. H. Bresnick, A. Bergman, M. P. Snyder, S. M. Weissman, D. Zheng, and A. I. Skoultchi, Proc. Natl Acad. Sci. USA **109**, 3832 (2012).

[111] N. Rekhtman, F. Radparvar, T. Evans, and A. I. Skoultchi, Genes Dev. **13**, 1398 (1999).

[112] B. Drogat, J. Kalucka, L. Gutiérrez, H. Hammad, S. Goossens, M. F. Ghahremani, S. Bartunkova, K. Haigh, K. Deswarte, O. Nyabi, *et al.*, Blood **116**, 2141 (2010).

[113] K. E. Elagib, M. Xiao, I. M. Hussaini, L. L. Delehanty, L. A. Palmer, F. K. Racke, M. J. Birrer, G. Shanmugasundaram, M. A. McDevitt, and A. N. Goldfarb, Mol. Cell. Biol. **24**, 7779 (2004).

[114] J. J. Tremblay, F. Hamel, and R. S. Viger, Endocrinology **143**, 3935 (2002).

[115] A. Murakami, H. Shen, S. Ishida, and C. Dickson, J. Biol. Chem. **279**, 28564 (2004).

[116] Y. Tian, L. Yuan, A. M. Goss, T. Wang, J. Yang, J. J. Lepore, D. Zhou, R. J. Schwartz, V. Patel, E. D. Cohen, *et al.*, Dev. Cell **18**, 275 (2010).

[117] A. Fischer, J. Klattig, B. Kneitz, H. Diez, M. Maier, B. Holtmann, C. Englert, and M. Gessler, Mol. Cell. Biol. **25**, 8960 (2005).

[118] Z. Ma, T. Swigut, A. Valouev, A. Rada-Iglesias, and J. Wysocka, Nat. Struct. Mol. Biol. **18**, 120 (2011).

[119] T. Shirai, S. Miyagi, D. Horiuchi, T. Okuda-Katayanagi, M. Nishimoto, M. Muramatsu,

Y. Sakamoto, M. Nagata, K. Hagiwara, and A. Okuda, J. Biol. Chem. **280**, 7244 (2005).

[120] H. Ushijima and M. Maeda, Biochem. Biophys. Res. Commun. **423**, 679 (2012).

[121] X. Guo and X.-F. Wang, Cell Res. **19**, 71 (2008).

[122] M. Katoh and M. Katoh, Int. J. Oncol. **31**, 461 (2007).

[123] M. Q. Hassan, R. Tare, S. H. Lee, M. Mandeville, B. Weiner, M. Montecino, A. J. Van Wijnen, J. L. Stein, G. S. Stein, and J. B. Lian, Mol. Cell. Biol. **27**, 3337 (2007).

[124] B. Xu, D. Geerts, K. Qian, H. Zhang, and G. Zhu, Hum. Reprod. **23**, 1394 (2008).

[125] X. Liu and C.-K. Qu, J. Signal Transduct. **2011** (2011).

[126] N. Chughtai, S. Schimchowitsch, J.-J. Lebrun, and S. Ali, J. Biol. Chem. **277**, 31107 (2002).

[127] L. Bei, C. Shah, H. Wang, W. Huang, R. Roy, and E. A. Eklund, J. Biol. Chem. **287**, 39589 (2012).

[128] C. Scholl, D. Bansal, K. Döhner, K. Eiwen, B. J. Huntly, B. H. Lee, F. G. Rücker, R. F. Schlenk, L. Bullinger, H. Döhner, *et al.*, J. Clin. Invest. **117**, 1037 (2007).

[129] N. Y.-G. Diman, S. Remacle, N. Bertrand, J. J. Picard, S. Zaffran, and R. Rezsohazy, PLoS One **6**, e27624 (2011).

[130] D. Su and L. J. Gudas, Biochem. Pharmacol. **75**, 1129 (2008).

[131] P. M. In der Rieden, F. L. Vilaspasa, and A. J. Durston, Dev. Dyn. **239**, 126 (2010).

[132] F. Mechta-Grigoriou, F. Giudicelli, C. Pujades, P. Charnay, and M. Yaniv, Dev. Biol. **258**, 419 (2003).

[133] B. L. Kidder, J. Yang, and S. Palmer, PLoS One **3**, e3932 (2008).

[134] K. Ross, A. K. Sedello, G. P. Todd, M. Paszkowski-Rogacz, A. W. Bird, L. Ding, T. Grinenko, K. Behrens, N. Hubner, M. Mann, *et al.*, Blood **119**, 4152 (2012).

[135] C. Collins, J. Wang, H. Miao, J. Bronstein, H. Nawer, T. Xu, M. Figueroa, A. G. Muntean, and J. L. Hess, Proc. Natl Acad. Sci. USA **111**, 9899 (2014).

[136] F. Weerkamp, T. Luis, B. Naber, E. Koster, L. Jeannotte, J. van Dongen, and F. Staal, Leukemia **20**, 1967 (2006).

[137] C. M. Trivedi, R. C. Patel, and C. V. Patel, Gene **408**, 187 (2008).

[138] H. Wang, T. H. Peiris, A. Mowery, J. Le Lay, Y. Gao, and L. E. Greenbaum, Mol. Endocrinol. **22**, 1596 (2008).

[139] J. Sonoda, J. Laganière, I. R. Mehl, G. D. Barish, L.-W. Chong, X. Li, I. E. Scheffler,

D. C. Mock, A. R. Bataille, F. Robert, *et al.*, Genes Dev. **21**, 1909 (2007).

[140] C. Villeneuve, C. Guilbeau-Frugier, P. Sicard, O. Lairez, C. Ordener, T. Duparc, D. De Paulis, B. Couderc, O. Spreux-Varoquaux, F. Tortosa, *et al.*, Antioxid. Redox sign. **18**, 5 (2013).

[141] Z. Wu, E. D. Rosen, R. Brun, S. Hauser, G. Adelmant, A. E. Troy, C. McKeon, G. J. Darlington, and B. M. Spiegelman, Mol. Cell **3**, 151 (1999).

[142] F. Oger, J. Dubois-Chevalier, C. Gheeraert, S. Avner, E. Durand, P. Froguel, G. Salbert, B. Staels, P. Lefebvre, and J. Eeckhoute, J. Biol. Chem. **289**, 708 (2014).

[143] F. Marchildon, C. St-Louis, R. Akter, V. Roodman, and N. L. Wiper-Bergeron, J. Biol. Chem. **285**, 13274 (2010).

[144] H. Xing, J. P. Northrop, J. R. Grove, K. E. Kilpatrick, J.-L. Su, and G. M. Ringold, Endocrinology **138**, 2776 (1997).

[145] A. Gillich, S. Bao, N. Grabole, K. Hayashi, M. W. Trotter, V. Pasque, E. Magnúsdóttir, and M. A. Surani, Cell Stem Cell **10**, 425 (2012).

[146] C. Yeamans, D. Wang, I. Paz-Priel, B. E. Torbett, D. G. Tenen, and A. D. Friedman, Blood **110**, 3136 (2007).

[147] Z. Hu, X. Gu, K. Baraoidan, V. Ibanez, A. Sharma, S. Kadkol, R. Munker, S. Ackerman, G. Nucifora, and Y. Saunthararajah, Blood **117**, 6498 (2011).

[148] G. Huang, P. Zhang, H. Hirai, S. Elf, X. Yan, Z. Chen, S. Koschmieder, Y. Okuno, T. Dayaram, J. D. Growney, *et al.*, Nat. Genet. **40**, 51 (2007).

[149] G. Aue, Y. Du, S. M. Cleveland, S. B. Smith, U. P. Davé, D. Liu, M. A. Weniger, J. Y. Metais, N. A. Jenkins, N. G. Copeland, *et al.*, Blood **118**, 4674 (2011).

[150] K. E. Elagib and A. N. Goldfarb, Crit. Rev. Eukaryot. Gene Expr. **17**, 271 (2007).

[151] C. E. Burns, D. Traver, E. Mayhall, J. L. Shepard, and L. I. Zon, Genes Dev. **19**, 2331 (2005).

[152] X. Fang, J.-G. Yoon, L. Li, W. Yu, J. Shao, D. Hua, S. Zheng, L. Hood, D. R. Goodlett, G. Foltz, *et al.*, BMC Genomics **12**, 11 (2011).

[153] N. Pencovich, R. Jaschek, A. Tanay, and Y. Groner, Blood **117**, e1 (2011).

[154] M. Iacovino, D. Chong, I. Szatmari, L. Hartweck, D. Rux, A. Caprioli, O. Cleaver, and M. Kyba, Nat. Cell Biol. **13**, 72 (2011).

[155] S. Ogawa, M. Satake, and K. Ikuta, J. Biochem. (Tokyo) **143**, 695 (2008).

[156] Y. Wang, R. M. Belflower, Y.-F. Dong, E. M. Schwarz, R. J. O'Keefe, and H. Drissi, J. Bone Miner. Res. **20**, 1624 (2005).

[157] J. Massagué and D. Wotton, EMBO J. **19**, 1745 (2000).

[158] M.-H. Lee, Y.-J. Kim, H.-J. Kim, H.-D. Park, A.-R. Kang, H.-M. Kyung, J.-H. Sung, J. M. Wozney, and H.-M. Ryoo, J. Biol. Chem. **278**, 34387 (2003).

[159] N. Wiper-Bergeron, C. St-Louis, and J. M. Lee, Mol. Endocrinol. **21**, 2124 (2007).

[160] K.-N. Lee, W.-G. Jang, E.-J. Kim, S.-H. Oh, H.-J. Son, S.-H. Kim, R. Franceschi, X.-K. Zhang, S.-E. Lee, and J.-T. Koh, J. Biol. Chem. **287**, 18888 (2012).

[161] F. Engin, T. Bertin, O. Ma, M. M. Jiang, L. Wang, R. E. Sutton, L. A. Donehower, and B. Lee, Hum. Mol. Genet. **18**, 1464 (2009).

[162] J. Lengler, T. Bittner, D. Münster, A.-D. Gawad, and J. Graw, Ophthalmic Res. **37**, 301 (2005).

[163] K. Liu, M. Jiang, Y. Lu, H. Chen, J. Sun, S. Wu, W.-Y. Ku, H. Nakagawa, Y. Kita, S. Natsugoe, *et al.*, Cell Stem Cell **12**, 304 (2013).

[164] T. Okubo, L. H. Pevny, and B. L. Hogan, Genes Dev. **20**, 2654 (2006).

[165] M. Marqués-Torrejón, E. Porlan, A. Banito, E. Gómez-Ibarlucea, A. J. Lopez-Contreras, Ó. Fernández-Capetillo, A. Vidal, J. Gil, J. Torres, and I. Fariñas, Cell Stem Cell **12**, 88 (2013).

[166] M. Saegusa, M. Hashimura, and T. Kuwata, Lab. Invest. **92**, 511 (2012).

[167] N. Geijsen, I. J. Uings, C. Pals, J. Armstrong, M. McKinnon, J. A. Raaijmakers, J.-W. J. Lammers, L. Koenderman, and P. J. Coffer, Science **293**, 1136 (2001).

[168] S. J. Vervoort, A. R. Lourenço, R. van Boxtel, and P. J. Coffer, PLoS One **8**, e53238 (2013).

[169] C. D. Scharer, C. D. McCabe, M. Ali-Seyed, M. F. Berger, M. L. Bulyk, and C. S. Moreno, Cancer Res. **69**, 709 (2009).

[170] H. Zhang, M. Alberich-Jorda, G. Amabile, H. Yang, P. B. Staber, A. DiRuscio, R. S. Welner, A. Ebralidze, J. Zhang, E. Levantini, *et al.*, Cancer Cell **24**, 575 (2013).

[171] K. Harada, A. Ogai, T. Takahashi, M. Kitakaze, H. Matsubara, and H. Oh, J. Biol. Chem. **283**, 26705 (2008).

[172] X. Zhou, S.-Y. Huang, J.-X. Feng, Y.-Y. Gao, L. Zhao, J. Lu, B.-Q. Huang, and Y. Zhang, World J. Gastroenterol. **17**, 4922 (2011).

[173] F. Nakaki and M. Saitou, Trends Biochem. Sci. **39**, 289 (2014).

[174] Z. Huang, H. Xu, and L. Sandell, J. Bone Miner. Res. **19**, 245 (2004).

[175] W. Huang, X. Zhou, V. Lefebvre, and B. de Crombrugghe, Mol. Cell. Biol. **20**, 4149 (2000).

[176] J. Park, J. J. Zhang, A. Moro, M. Kushida, M. Wegner, and P. C. Kim, Dev. Dyn. **239**, 514 (2010).

[177] T. Furumatsu, M. Tsuda, N. Taniguchi, Y. Tajima, and H. Asahara, J. Biol. Chem. **280**, 8343 (2005).

[178] Y. Kim, A. Kobayashi, R. Sekido, L. DiNapoli, J. Brennan, M.-C. Chaboissier, F. Poulat, R. R. Behringer, R. Lovell-Badge, and B. Capel, PLoS Biol. **4**, e187 (2006).

[179] S. Murakami, V. Lefebvre, and B. de Crombrugghe, J. Biol. Chem. **275**, 3687 (2000).

[180] J. S. Rockel, J. C. Kudirka, A. J. Guzi, and S. M. Bernier, Arthritis Res. Ther. **10**, R3 (2008).

[181] I. Sekiya, P. Koopman, K. Tsuji, S. Mertin, V. Harley, Y. Yamada, K. Shinomiya, A. Niguji, and M. Noda, J. Cell. Biochem. **81**, 71 (2001).

[182] S.-G. Hwang, S.-S. Yu, H. Poo, and J.-S. Chun, J. Biol. Chem. **280**, 29780 (2005).

[183] R. M. Raices, Y. Kannan, V. Bellamkonda-Athmaram, S. Seshadri, H. Wang, D. C. Guttridge, and M. D. Wewers, PLoS One **4**, e6776 (2009).

[184] A. W. O'Regan, J. M. Hayden, and J. S. Berman, J. Leukoc. Biol. **68**, 495 (2000).

[185] M. Ono, H. Yaguchi, N. Ohkura, I. Kitabayashi, Y. Nagamura, T. Nomura, Y. Miyachi, T. Tsukada, and S. Sakaguchi, Nature **446**, 685 (2007).

[186] M. Afkarian, J. R. Sedy, J. Yang, N. G. Jacobson, N. Cereb, S. Y. Yang, T. L. Murphy, and K. M. Murphy, Nat. Immunol. **3**, 549 (2002).

[187] J. Fields, A. Ghorpade, *et al.*, J. Neuroinflammation **9**, 1 (2012).

[188] W. E. Paul, ed., *Fundamental Immunology*, 7th ed. (Lippincot Williams and Wilkins, Philadelphia, 2012).

[189] J. Clin. Invest. **107**, 1055 (2001).

[190] V. D. Joshi, D. V. Kalvakolanu, W. Chen, L. Zhang, T. J. Kang, K. E. Thomas, S. N. Vogel, and A. S. Cross, Journal of interferon & cytokine research **26**, 739 (2006).

[191] K. Kirtikara, R. Raghow, S. J. Laulederkind, S. Goorha, T. Kanekura, and L. R. Ballou, Mol. Cell. Biochem. **203**, 41 (2000).

[192] Y. Kitagishi, M. Kobayashi, K. Kikuta, and S. Matsuda, Depress. Res. Treat. **2012** (2012).

[193] A. B. Roos and M. Nord, J. Endocrinol. **212**, 291 (2012).

[194] T. B. van Dijk, B. Baltus, J. A. Raaijmakers, J.-W. J. Lammers, L. Koenderman, and R. P. de Groot, J. Immunol. **163**, 2674 (1999).

[195] X. Zhao, V. Jankovic, A. Gural, G. Huang, A. Pardanani, S. Menendez, J. Zhang, R. Dunne, A. Xiao, H. Erdjument-Bromage, *et al.*, Genes Dev. **22**, 640 (2008).

[196] S. Nimer, J. Zhang, H. Avraham, and Y. Miyazaki, Blood **88**, 66 (1996).

[197] C. Robin, K. Ottersbach, C. Durand, M. Peeters, L. Vanes, V. Tybulewicz, and E. Dzierzak, Dev. Cell **11**, 171 (2006).

[198] G. Xu, Y. Zhang, L. Zhang, A. I. Roberts, and Y. Shi, Stem Cells **27**, 942 (2009).

[199] E. Mahdipour, J. C. Charnock, and K. A. Mace, Blood **117**, 815 (2011).

[200] S. J. Lee, E. Borsting, A.-E. Declèves, P. Singh, and R. Cunard, Nephron Exp. Nephrol. **121**, e86 (2012).

[201] S. Sawatsri, N. Desai, J. A. Rock, and N. Sidell, Fertil. Steril. **73**, 1012 (2000).

[202] S. Chen, Y. Xu, Y. Chen, X. Li, W. Mou, L. Wang, Y. Liu, R. A. Reisfeld, R. Xiang, D. Lv, *et al.*, PLoS One **7**, e36326 (2012).

[203] C. Kunsch, R. K. Lang, C. A. Rosen, and M. F. Shannon, J. Immunol. **153**, 153 (1994).

[204] E. Hoffmann, O. Dittrich-Breiholz, H. Holtmann, and M. Kracht, J. Leukoc. Biol. **72**, 847 (2002).

[205] Q. Wang, N. Huber, G. Noel, L. Haar, Y. Shan, T. A. Pritts, and C. K. Ogle, Inflamm. Res. **61**, 977 (2012).

[206] S. Lu and Z. Dong, Prostate **66**, 996 (2006).

[207] N. M. Gharavi, J. A. Alva, K. P. Mouillesseaux, C. Lai, M. Yeh, W. Yeung, J. Johnson, W. L. Szeto, L. Hong, M. Fishbein, *et al.*, J. Biol. Chem. **282**, 31460 (2007).

[208] J. Wanninger, M. Neumeier, J. Weigert, S. Bauer, T. S. Weiss, A. Schäffler, C. Krempl, C. Bleyl, C. Aslanidis, J. Schölmerich, *et al.*, Am. J. Physiol. **297**, G611 (2009).

[209] M. J. May and S. Ghosh, Immunol. Today **19**, 80 (1998).

[210] F. Meng and S. R. D'Mello, Biochim. Biophys. Acta **1630**, 35 (2003).

[211] X. Yan, Z. Liu, and Y. Chen, Acta Biochim. Biophys. Sinica **41**, 263 (2009).

[212] D. Bai, L. Ueno, and P. K. Vogt, Int. J. Cancer **125**, 2863 (2009).

[213] M. Ohtsubo, A. Takayanagi, S. Gamou, and N. Shimizu, J. Cell Physiol. **184**, 131 (2000).

[214] E. Dudley, F. Hornung, L. Zheng, D. Scherer, D. Ballard, and M. Lenardo, Eur. J Immunol. **29**, 878 (1999).

[215] C. Cappello, A. Zwergal, S. Kanclerski, S. C. Haas, J. D. Kandemir, R. Huber, S. Page, and K. Brand, Cell Signal. **21**, 1918 (2009).

[216] J. L. Cheshire and A. Baldwin, Mol. Cell. Biol. **17**, 6746 (1997).

[217] L. A. Solt, L. A. Madge, J. S. Orange, and M. J. May, J. Biol. Chem. **282**, 8724 (2007).

[218] H. Mutoh, H. Hayakawa, H. Sakamoto, and K. Sugano, J. Gastroenterol. **42**, 719 (2007).

[219] A. Remels, R. Langen, H. R. Gosker, A. Russell, F. Spaapen, J. Voncken, P. Schrauwen, and A. M. Schols, Am. J. Physiol. Endocrinol. Metab. **297**, E174 (2009).

[220] J. Torres and F. M. Watt, Nat. Cell Biol. **10**, 194 (2008).

[221] I. Najjar, F. Baran-Marszak, C. Le Clorennec, C. Laguillier, O. Schischmanoff, I. Youlyouz-Marfak, M. Schlee, G. W. Bornkamm, M. Raphaël, J. Feuillard, *et al.*, J. Virol. **79**, 4936 (2005).

[222] C. V. Ramana, M. Chatterjee-Kishore, H. Nguyen, and G. R. Stark, Oncogene **19**, 2619 (2000).

[223] C. Gao, H. Guo, Z. Mi, M. J. Grusby, and P. C. Kuo, J. Immunol. **178**, 1870 (2007).

[224] B. Kwabi-Addo, M. Ozen, and M. Ittmann, Endocr. Relat. Cancer **11**, 709 (2004).

[225] S.-S. Tian, P. Tapley, C. Sincich, R. B. Stein, J. Rosen, and P. Lamb, Blood **88**, 4435 (1996).

[226] M. Berishaj, S. P. Gao, S. Ahmed, K. Leslie, H. Al-Ahmadie, W. L. Gerald, W. Bornmann, J. F. Bromberg, *et al.*, Breast Cancer Res. **9**, R32 (2007).

[227] M. Burger, T. Hartmann, J. A. Burger, and I. Schraufstatter, Oncogene **24**, 2067 (2005).

[228] R. Behera, V. Kumar, K. Lohite, S. Karnik, and G. C. Kundu, Carcinogenesis **31**, 192 (2010).

[229] M. Bartoli, D. PLATT, T. Lemtalsi, X. Gu, S. E. Brooks, M. B. Marrero, and R. B. Caldwell, FASEB J. **17**, 1562 (2003).

[230] S. Ezoe, I. Matsumura, K. Gale, Y. Satoh, J. Ishikawa, M. Mizuki, S. Takahashi, N. Minegishi, K. Nakajima, M. Yamamoto, *et al.*, J. Biol. Chem. **280**, 13163 (2005).

[231] L. H. Wang, X. Y. Yang, X. Zhang, J. Huang, J. Hou, J. Li, H. Xiong, K. Mihalic, H. Zhu, W. Xiao, *et al.*, Immunity **20**, 205 (2004).

[232] J. Zhou, J. Wulfkuhle, H. Zhang, P. Gu, Y. Yang, J. Deng, J. B. Margolick, L. A. Liotta, E. Petricoin, and Y. Zhang, Proc. Natl Acad. Sci. USA **104**, 16158 (2007).

[233] S. Sun and B. M. Steinberg, J. Gen. Virol. **83**, 1651 (2002).

[234] Z. Syed, S. B. Cheepala, J. N. Gill, J. Stein, C. A. Nathan, J. DiGiovanni, V. Batra, P. Adegboyega, H. E. Kleiner, and J. L. Clifford, Cancer Prev. Res. **2**, 903 (2009).

[235] J. P. Cerliani, T. Guillardoy, S. Giulianelli, J. P. Vaque, J. S. Gutkind, S. I. Vanzulli, R. Martins, E. Zeitlin, C. A. Lamb, and C. Lanari, Cancer Res. **71**, 3720 (2011).

[236] A. x. Dudley, D. Thomas, J. Best, and A. Jenkins, Biochem. J. **390**, 427 (2005).

[237] C. A. Shah, L. Bei, H. Wang, L. C. Platanias, and E. A. Eklund, J. Biol. Chem. **287**, 18230 (2012).

[238] S. A. Vetrone, E. Montecino-Rodriguez, E. Kudryashova, I. Kramerova, E. P. Hoffman, S. D. Liu, M. C. Miceli, M. J. Spencer, *et al.*, J. Clin. Invest. **119**, 1583 (2009).

[239] C. Zhang, T. Basta, and M. W. Klymkowsky, Dev. Dyn. **234**, 878 (2005).

[240] R.-Y. Liu, Y. Zeng, Z. Lei, L. Wang, H. Yang, Z. Liu, J. Zhao, and H.-T. Zhang, Int. J. Oncol. **44**, 1643 (2014).

[241] J. P. Annes, J. S. Munger, and D. B. Rifkin, J. Cell Sci. **116**, 217 (2003).

[242] M. Cordenonsi, S. Dupont, S. Maretto, A. Insinga, C. Imbriano, and S. Piccolo, Cell **113**, 301 (2003).

[243] R. Elston and G. J. Inman, J. Signal Transduct. **2012** (2012).

[244] J. Qin, S.-P. Wu, C. J. Creighton, F. Dai, X. Xie, C.-M. Cheng, A. Frolov, G. Ayala, X. Lin, X.-H. Feng, *et al.*, Nature **493**, 236 (2013).

[245] G. Salbert, A. Fanjul, F. J. Piedrafita, X. P. Lu, S.-J. Kim, P. Tran, and M. Pfahl, Mol. Endocrinol. **7**, 1347 (1993).

[246] L. Ulloa, J. Doody, and J. Massagué, Nature **397**, 710 (1999).

[247] A. Hosui, A. Kimura, D. Yamaji, B.-m. Zhu, R. Na, and L. Hennighausen, J. Exp. Med. **206**, 819 (2009).

[248] E.-L. Alarmo and A. Kallioniemi, Endocr. Relat. Cancer **17**, R123 (2010).

[249] J. Huang, L. K. Dattilo, R. Rajagopal, Y. Liu, V. Kaartinen, Y. Mishina, C.-X. Deng, L. Umans, A. Zwijsen, A. B. Roberts, *et al.*, Development **136**, 1741 (2009).

[250] L. Rong, J. Liu, Y. Qi, A. M. Graham, M. S. Parmacek, and S. Li, Mol. Biol. Cell **23**, 3754 (2012).

[251] M. Keita, M. Bachvarova, C. Morin, M. Plante, J. Gregoire, M.-C. Renaud, A. Sebastianelli, X. B. Trinh, and D. Bachvarov, Cell Cycle **12**, 972 (2013).

[252] D. A. Lim, A. D. Tramontin, J. M. Trevejo, D. G. Herrera, J. M. García-Verdugo, and A. Alvarez-Buylla, Neuron **28**, 713 (2000).

[253] J. Wang, J. W. Park, H. Drissi, X. Wang, and R.-H. Xu, J. Biol. Chem. **289**, 2384 (2014).

[254] E. Grönroos, I. J. Kingston, A. Ramachandran, R. A. Randall, P. Vizán, and C. S. Hill, Mol. Cell. Biol. **32**, 2904 (2012).

[255] L. Gunaratnam, M. Morley, A. Franovic, N. de Paulsen, K. Mekhail, D. A. Parolin, E. Nakamura, I. A. Lorimer, and S. Lee, J. Biol. Chem. **278**, 44966 (2003).

[256] D. F. Stern, A. Roberts, N. Roche, M. Sporn, and R. Weinberg, Mol. Cell. Biol. **6**, 870 (1986).

[257] E.-S. Park, S. Choi, K. N. Muse, T. E. Curry Jr, and M. Jo, Endocrinology **149**, 3025 (2008).

[258] T. R. Fenton, D. Nathanson, C. P. de Albuquerque, D. Kuga, A. Iwanami, J. Dang, H. Yang, K. Tanaka, S. M. Oba-Shinjo, M. Uno, *et al.*, Proc. Natl Acad. Sci. USA **109**, 14164 (2012).

[259] N. Quarto and F. Amalric, J. Cell Sci. **107**, 3201 (1994).

[260] C. Palmieri, D. Roberts-Clark, A. Assadi-Sabet, R. Coope, M. O'Hare, A. Sunters, A. Hanby, M. Slade, J. Gomm, E. Lam, *et al.*, J. Endocrinol. **177**, 65 (2003).

[261] Y. Lu, Y. Li, A. C. Cavender, S. Wang, A. Mansukhani, and R. N. D'Souza, Dev. Dyn. **241**, 1708 (2012).

[262] M. Yamaji, J. Ueda, K. Hayashi, H. Ohta, Y. Yabuta, K. Kurimoto, R. Nakato, Y. Yamada, K. Shirahige, and M. Saitou, Cell Stem Cell **12**, 368 (2013).

[263] D.-E. Zhang, P. Zhang, N.-d. Wang, C. J. Hetherington, G. J. Darlington, and D. G. Tenen, Proc. Natl Acad. Sci. USA **94**, 569 (1997).

[264] S. M. Dunn, L. S. Coles, R. K. Lang, S. Gerondakis, M. A. Vadas, and M. F. Shannon, Blood **83**, 2469 (1994).

[265] L. T. Smith, S. Hohaus, D. Gonzalez, S. Dziennis, and D. Tenen, Blood **88**, 1234 (1996).

[266] J. Heineke, M. Auger-Messier, J. Xu, T. Oka, M. A. Sargent, A. York, R. Klevitsky, S. Vaikunth, S. A. Duncan, B. J. Aronow, *et al.*, J. Clin. Invest. **117**, 3198 (2007).

[267] E. B. Rankin, J. Rha, T. L. Unger, C. H. Wu, H. P. Shutt, R. S. Johnson, M. C. Simon,

B. Keith, and V. H. Haase, Oncogene **27**, 5354 (2008).

[268] F. A. Pereira, Y. Qiu, G. Zhou, M.-J. Tsai, and S. Y. Tsai, Genes Dev. **13**, 1037 (1999).

[269] Z. Arany, S.-Y. Foo, Y. Ma, J. L. Ruas, A. Bommi-Reddy, G. Girnun, M. Cooper, D. Laznik, J. Chinsomboon, S. M. Rangwala, *et al.*, Nature **451**, 1008 (2008).

[270] N. D. Lawson, A. M. Vogel, and B. M. Weinstein, Dev. Cell **3**, 127 (2002).

[271] J. E. Jung, H. G. Lee, I. H. Cho, D. H. Chung, S.-H. Yoon, Y. M. Yang, J. W. Lee, S. Choi, J.-W. Park, S.-K. Ye, *et al.*, FASEB J. **19**, 1296 (2005).

[272] G. Niu, K. L. Wright, M. Huang, L. Song, E. Haura, J. Turkson, S. Zhang, T. Wang, D. Sinibaldi, D. Coppola, *et al.*, Oncogene **21**, 2000 (2002).

[273] Q. Xu, J. Briggs, S. Park, G. Niu, M. Kortylewski, S. Zhang, T. Gritsko, J. Turkson, H. Kay, G. L. Semenza, *et al.*, Oncogene **24**, 5552 (2005).

[274] C. N. Nagineni, W. Samuel, S. Nagineni, K. Pardhasaradhi, B. Wiggert, B. Detrick, and J. J. Hooks, J. Cell Physiol. **197**, 453 (2003).

[275] G. Ferrari, B. D. Cook, V. Terushkin, G. Pintucci, and P. Mignatti, J. Cell Physiol. **219**, 449 (2009).

[276] N. Sidell, Y. Feng, L. Hao, J. Wu, J. Yu, M. A. Kane, J. L. Napoli, and R. N. Taylor, Mol. Endocrinol. **24**, 148 (2010).

[277] T. Hattori, C. Müller, S. Gebhard, E. Bauer, F. Pausch, B. Schlund, M. R. Bösl, A. Hess, C. Surmann-Schmitt, H. von der Mark, *et al.*, Development **137**, 901 (2010).

[278] T. Galbaugh, M. G. Cerrito, C. C. Jose, and M. L. Cutler, BMC Cell Biol. **7**, 34 (2006).

[279] F. Lanner and J. Rossant, Development **137**, 3351 (2010).

[280] T. Palomero, M. L. Sulis, M. Cortina, P. J. Real, K. Barnes, M. Ciofani, E. Caparros, J. Buteau, K. Brown, S. L. Perkins, *et al.*, Nat. Med. **13**, 1203 (2007).

[281] J. T. Stuenæs, A. Bolling, A. Ingvaldsen, C. Rommundstad, E. Sudar, F.-C. Lin, Y.-C. Lai, and J. Jensen, Br. J. Pharmacol. **160**, 116 (2010).

[282] E. Castellano and J. Downward, Genes Cancer **2**, 261 (2011).

[283] Z. Shao, K. Bhattacharya, E. Hsich, L. Park, B. Walters, U. Germann, Y.-M. Wang, J. Kyriakis, R. Mohanlal, K. Kuida, *et al.*, Circ. Res. **98**, 111 (2006).

[284] K. A. Waite and C. Eng, Am. J. Hum. Genet. **70**, 829 (2002).

[285] E. V. Maytin, J. C. Lin, R. Krishnamurthy, N. Batchvarova, D. Ron, P. J. Mitchell, and J. F. Habener, Dev. Biol. **216**, 164 (1999).

[286] M. A. Hubert, S. L. Sherritt, C. J. Bachurski, and S. Handwerger, PLoS One **5**, e9417 (2010).

[287] B. Lüscher, P. J. Mitchell, T. Williams, and R. Tjian, Genes Dev. **3**, 1507 (1989).

[288] T. Kitamuro, K. Takahashi, K. Ogawa, R. Udono-Fujimori, K. Takeda, K. Furuyama, M. Nakayama, J. Sun, H. Fujita, W. Hida, *et al.*, J. Biol. Chem. **278**, 9125 (2003).

[289] Y. Okita, A. Kamoshida, H. Suzuki, K. Itoh, H. Motohashi, K. Igarashi, M. Yamamoto, T. Ogami, D. Koinuma, and M. Kato, J. Biol. Chem. **288**, 20658 (2013).

[290] Y.-M. Go, Y. C. Boo, H. Park, M. C. Maland, R. Patel, K. A. Pritchard, Y. Fujio, K. Walsh, V. Darley-Usmar, and H. Jo, J. Appl. Physiol. **91**, 1574 (2001).

[291] R. Kajanne, P. Miettinen, A. Mehlem, S.-K. Leivonen, M. Birrer, M. Foschi, V.-M. Kähäri, and S. Leppä, J. Cell Physiol. **212**, 489 (2007).

[292] A. Mukhopadhyay, S. Shishodia, X.-Y. Fu, and B. B. Aggarwal, J. Cell. Biochem. **84**, 803 (2002).

[293] J. Rangatia, R. K. Vangala, N. Treiber, P. Zhang, H. Radomska, D. G. Tenen, W. Hiddemann, and G. Behre, Mol. Cell. Biol. **22**, 8681 (2002).

[294] G. L. Semenza, Cancer Metastasis. Rev. **19**, 59 (2000).

[295] T. K. Guren, H. Abrahamsen, G. H. Thoresen, E. Babaie, T. Berg, and T. Christoffersen, Biochem. Biophys. Res. Commun. **258**, 565 (1999).

[296] A. Lonic, E. F. Barry, C. Quach, B. Kobe, N. Saunders, and M. A. Guthridge, Mol. Cell. Biol. **28**, 3372 (2008).

[297] N. Kim, S. Kukkonen, M. D. P. Martinez-Viedma, S. Gupta, and A. Aldovini, Blood **121**, 4090 (2013).

[298] B. P. Eliceiri, Circ. Res. **89**, 1104 (2001).

[299] E. More, T. Fellner, H. Doppelmayr, C. Hauser-Kronberger, N. Dandachi, P. Obrist, F. Sandhofer, and B. Paulweber, J. Endocrinol. **176**, 83 (2003).

[300] O. S. Gardner, B. J. Dewar, and L. M. Graves, Mol. Pharmacol. **68**, 933 (2005).

[301] M. K. Lee, C. Pardoux, M. C. Hall, P. S. Lee, D. Warburton, J. Qing, S. M. Smith, and R. Derynck, EMBO J. **26**, 3957 (2007).

[302] K. Ito, Z. Maruyama, A. Sakai, S. Izumi, T. Moriishi, C. Yoshida, T. Miyazaki, H. Komori, K. Takada, H. Kawaguchi, *et al.*, Oncogene **33**, 1862 (2014).

[303] A. Takaoka, S. Hayakawa, H. Yanai, D. Stoiber, H. Negishi, H. Kikuchi, S. Sasaki, K. Imai,

T. Shibue, K. Honda, *et al.*, Nature **424**, 516 (2003).

[304] D. Bonofiglio, E. Cione, H. Qi, A. Pingitore, M. Perri, S. Catalano, D. Vizza, M. L. Panno, G. Genchi, S. A. Fuqua, *et al.*, Am. J. Pathol. **175**, 1270 (2009).

[305] V. Stambolic, D. MacPherson, D. Sas, Y. Lin, B. Snow, Y. Jang, S. Benchimol, and T. Mak, Mol. Cell **8**, 317 (2001).

[306] J. C. Curtin, K. H. Dragnev, D. Sekula, A. J. Christie, E. Dmitrovsky, and M. J. Spinella, Oncogene **20**, 2559 (2001).

[307] Y. Dohi, T. Ikura, Y. Hoshikawa, Y. Katoh, K. Ota, A. Nakanome, A. Muto, S. Omura, T. Ohta, A. Ito, *et al.*, Nat. Struct. Mol. Biol. **15**, 1246 (2008).

[308] X. Pan, J. Zhao, W.-N. Zhang, H.-Y. Li, R. Mu, T. Zhou, H.-Y. Zhang, W.-L. Gong, M. Yu, J.-H. Man, *et al.*, Proc. Natl Acad. Sci. USA **106**, 3788 (2009).

[309] A. Bellis, D. Castaldo, V. Trimarco, M. G. Monti, P. Chivasso, J. Sadoshima, B. Trimarco, and C. Morisco, Arterioscler. Thromb. Vasc. Biol. **29**, 1207 (2009).

[310] G. Tortora, V. Damiano, C. Bianco, G. Baldassarre, A. R. Bianco, L. Lanfrancone, P. G. Pelicci, and F. Ciardiello, Oncogene **14**, 923 (1997).

[311] H. Yang, G. Li, J.-J. Wu, L. Wang, M. Uhler, and D. M. Simeone, J. Biol. Chem. **288**, 8737 (2013).

[312] L. Zhang, C. J. Duan, C. Binkley, G. Li, M. D. Uhler, C. D. Logsdon, and D. M. Simeone, Mol. Cell. Biol. **24**, 2169 (2004).

[313] O. V. Leontieva, A. V. Gudkov, and M. V. Blagosklonny, Cell Cycle **9**, 4323 (2010).

[314] H.-J. Choi, T.-W. Chung, S.-J. Kim, S.-Y. Cho, Y.-S. Lee, Y.-C. Lee, J.-H. Ko, and C.-H. Kim, Glycobiology **18**, 395 (2008).

[315] S. Y. Lee, G. Y. Hur, K. H. Jung, H. C. Jung, S. Y. Lee, J. H. Kim, C. Shin, J. J. Shim, K. H. In, K. H. Kang, *et al.*, Lung Cancer **51**, 297 (2006).

[316] Z. Chen, L. C. Trotman, D. Shaffer, H.-K. Lin, Z. A. Dotan, M. Niki, J. A. Koutcher, H. I. Scher, T. Ludwig, W. Gerald, *et al.*, Nature **436**, 725 (2005).

[317] K. M. Vasudevan, S. Gurumurthy, and V. M. Rangnekar, Mol. Cell. Biol. **24**, 1007 (2004).

[318] N. M. Teplyuk, L. M. Haupt, L. Ling, C. Dombrowski, F. K. Mun, S. S. Nathan, J. B. Lian, J. L. Stein, G. S. Stein, S. M. Cool, *et al.*, J. Cell. Biochem. **107**, 144 (2009).

[319] V. Kolm, U. Sauer, B. Olgemooller, and E. Schleicher, Am. J. Physiol. Renal. Physiol. **270**, F812 (1996).

[320] D. Woods, H. Cherwinski, E. Venetsanakos, A. Bhat, S. Gysin, M. Humbert, P. F. Bray, V. L. Saylor, and M. McMahon, Mol. Cell. Biol. **21**, 3192 (2001).

[321] S. Nakao, T. Kuwano, T. Ishibashi, M. Kuwano, and M. Ono, J. Immunol. **170**, 5704 (2003).

[322] A. Wilson, M. J. Murphy, T. Oskarsson, K. Kaloulis, M. D. Bettess, G. M. Oser, A.-C. Pasche, C. Knabenhans, H. R. MacDonald, and A. Trumpp, Genes Dev. **18**, 2747 (2004).

[323] P. Zhao and J. M. Stephens, Mol. Metab. **2**, 161 (2013).

[324] X. Wang, C. Blagden, J. Fan, S. J. Nowak, I. Taniuchi, D. R. Littman, and S. J. Burden, Genes Dev. **19**, 1715 (2005).

[325] E. Hirsinger, D. Duprez, C. Jouve, P. Malapert, J. Cooke, and O. Pourquié, Development **124**, 4605 (1997).

[326] B. K. Zehentner, A. Haussmann, and H. Burtscher, Dev. Growth. Differ. **44**, 1 (2002).

[327] M. I. Reinhold, M. Abe, R. M. Kapadia, Z. Liao, and M. C. Naski, J. Biol. Chem. **279**, 38209 (2004).

[328] S. Grainger, J. Lam, J. G. Savory, A. J. Mears, F. M. Rijli, and D. Lohnes, Dev. Biol. **361**, 1 (2012).

[329] T. Uesaka, N. Kageyama, and H. Watanabe, J. Mol. Biol. **337**, 647 (2004).

[330] B. G. Rash, H. D. Lim, J. J. Breunig, and F. M. Vaccarino, J. Neurosci. **31**, 15604 (2011).

[331] L. Espinosa, S. Cathelin, T. D'Altri, T. Trimarchi, A. Statnikov, J. Guiu, V. Rodilla, J. Inglés-Esteve, J. Nomdedeu, B. Bellosillo, *et al.*, Cancer Cell **18**, 268 (2010).

[332] T. Yoshimatsu, D. Kawaguchi, K. Oishi, K. Takeda, S. Akira, N. Masuyama, and Y. Gotoh, Development **133**, 2553 (2006).

[333] T. A. Mitsiadis, D. Graf, H. Luder, T. Gridley, and G. Bluteau, Development **137**, 3025 (2010).

[334] Y.-C. Shi, H. Zhao, C. Yin, X. Zeng, Q. Zhang, W.-P. Xu, J. Wei, F. Chen, and W.-F. Xie, Digestive and Liver Disease **45**, 844 (2013).

[335] O. Boucherat, J. Chakir, and L. Jeannotte, Biol. Open , BIO20121701 (2012).

[336] X. Chen, J. Qin, C.-M. Cheng, M.-J. Tsai, and S. Y. Tsai, Mol. Endocrinol. **26**, 1268 (2012).

[337] C. Neumann, F. Garreis, F. Paulsen, C. M. Hammer, M. T. Birke, and M. Scholz, PLoS One **9**, e92762 (2014).

[338] T. Nakamachi, T. Nomiyama, F. Gizard, E. B. Heywood, K. L. Jones, Y. Zhao, L. Fuentes, K. Takebayashi, Y. Aso, B. Staels, *et al.*, Diabetes **56**, 1662 (2007).

[339] A. Mazzucotelli, N. Viguerie, C. Tiraby, J.-S. Annicotte, A. Mairal, E. Klimcakova, E. Lepin, P. Delmar, S. Dejean, G. Tavernier, *et al.*, Diabetes **56**, 2467 (2007).

[340] S. Ali, A. Ahmad, S. Gadgeel, F. H. Sarkar, *et al.*, PLoS One **6**, e16068 (2011).

[341] I. Rios, R. Alvarez-Rodríguez, E. Martí, and S. Pons, Development **131**, 3159 (2004).

[342] M. F. Bastida, R. Sheth, and M. A. Ros, Development **136**, 3779 (2009).

[343] H. Mutoh, H. Hayakawa, M. Sashikawa, H. Sakamoto, and K. Sugano, Biochem. J **427**, 423 (2010).

[344] K. C. Schreck, P. Taylor, L. Marchionni, V. Gopalakrishnan, E. E. Bar, N. Gaiano, and C. G. Eberhart, Clin. Cancer Res. **16**, 6060 (2010).

[345] R. Malek, J. Matta, N. Taylor, M. E. Perry, and S. M. Mendrysa, PLoS One **6**, e17884 (2011).

[346] K. L. Auld, S. P. Berasi, Y. Liu, M. Cain, Y. Zhang, C. Huard, S. Fukayama, J. Zhang, S. Choe, W. Zhong, *et al.*, J. Mol. Endocrinol. **48**, 177 (2012).

[347] A. Akhmetshina, K. Palumbo, C. Dees, C. Bergmann, P. Venalis, P. Zerr, A. Horn, T. Kireva, C. Beyer, J. Zwerina, *et al.*, Nat. Commun. **3**, 735 (2012).

[348] X. Liu, X. Zhang, Q. Zhan, M. V. Brock, J. G. Herman, and M. Guo, Cancer Biol. Ther. **13**, 1152 (2012).

[349] A. Mansukhani, D. Ambrosetti, G. Holmes, L. Cornivelli, and C. Basilico, J. Cell Biol. **168**, 1065 (2005).

[350] L. Guo, D. Zhong, S. Lau, X. Liu, X.-Y. Dong, X. Sun, V. W. Yang, P. M. Vertino, C. S. Moreno, V. Varma, *et al.*, Mol. Cancer Res. **6**, 1421 (2008).

[351] D. W. Chan, C. S. Mak, T. H. Leung, K. K. Chan, and H. Y. Ngan, Oncotarget **3**, 1546 (2012).

[352] L. Topol, W. Chen, H. Song, T. F. Day, and Y. Yang, J. Biol. Chem. **284**, 3323 (2009).

[353] Y. Li, Y. Xia, Y. Wang, L. Mao, Y. Gao, Q. He, M. Huang, S. Chen, and B. Hu, Mol. Neurobiol. **47**, 967 (2013).

[354] L. A. McPherson, G. W. Woodfield, and R. J. Weigel, J. Surg. Res. **138**, 71 (2007).

[355] W. J. Housley, C. A. O'Conor, F. Nichols, L. Puddington, E. G. Lingenheld, L. Zhu, and R. B. Clark, J. Leukoc. Biol. **86**, 293 (2009).

[356] M. A. Antonyak, C. J. McNeill, J. J. Wakshlag, J. E. Boehm, and R. A. Cerione, J. Biol. Chem. **278**, 15859 (2003).

[357] S. Malpel, C. Mendelsohn, and W. V. Cardoso, Development **127**, 3057 (2000).

II. MODELING NETWORK DYNAMICS

A. Equations

The dynamical equation for the concentration (activity) of an agent x_i under the influence of other agents in the network is given by:

$$\frac{dx_i}{dt} = \frac{a \cdot (\sum_{u \in act} x_u^n)}{1 + a \cdot (\sum_{u \in act} x_u^n)} \cdot \frac{1}{1 + a \cdot (\sum_{v \in inh} x_v^n)} - x_i \tag{1}$$

where $i = 1, \ldots, 81$, act denotes the set of all nodes that activate node i and inh the set of nodes that inhibit node i. The values for each agent is normalized from zero to one. One may notice that these equations have a conservation property that the values of all agents will not go beyond the interval from zero to one while evolving with time. We next provide all equations used to generate the main result.

B. A Complete List of Equations Used in Simulating Network Dynamics

We list below all dynamical equations for modeling the network in Supplementary Table I:

$$\frac{dx_{Cdk2}}{dt} = \frac{a \cdot (x_{MAPK}^n)}{1 + a \cdot (x_{MAPK}^n)} \times \frac{1}{1 + a \cdot (x_{C/EBP\alpha}^n + x_{Gata1/2}^n)} - x_{Cdk2}$$

$$\frac{dx_{Cdk6}}{dt} = \frac{a \cdot (x_{Hoxa9}^n + x_{Pu.1}^n + x_{MAPK}^n)}{1 + a \cdot (x_{Hoxa9}^n + x_{Pu.1}^n + x_{MAPK}^n)} \times \frac{1}{1 + a \cdot (x_{Gata1/2}^n + x_{p53}^n)} - x_{Cdk6}$$

$$\frac{dx_{Cyclin\ D}}{dt} = \frac{a \cdot (x_{E2F}^n + x_{Myc}^n + x_{EGFR}^n)}{1 + a \cdot (x_{E2F}^n + x_{Myc}^n + x_{EGFR}^n)} \times \frac{1}{1 + a \cdot (x_{p27}^n + x_{p53}^n)} - x_{Cyclin\ D}$$

$$\frac{dx_{Cyclin\ E}}{dt} = \frac{a \cdot (x_{E2F}^n + x_{Myc}^n + x_{Bach1}^n)}{1 + a \cdot (x_{E2F}^n + x_{Myc}^n + x_{Bach1}^n)} \times \frac{1}{1 + a \cdot (x_{p21}^n + x_{p27}^n + x_{PTEN}^n)} - x_{Cyclin\ E}$$

$$\frac{dx_{E2F}}{dt} = \frac{a \cdot (x_{E2F}^n + x_{Myc}^n)}{1 + a \cdot (x_{E2F}^n + x_{Myc}^n)} \times \frac{1}{1 + a \cdot (x_{p21}^n)} - x_{E2F}$$

$$\frac{dx_{Myc}}{dt} = \frac{a \cdot (x_{E2F}^n + x_{pRb}^n + x_{MAPK}^n + x_{Notch}^n + x_{SHH}^n + x_{Wnt}^n)}{1 + a \cdot (x_{E2F}^n + x_{pRb}^n + x_{MAPK}^n + x_{Notch}^n + x_{SHH}^n + x_{Wnt}^n)} \times \frac{1}{1 + a \cdot (x_{TGF-\beta}^n + x_{AP2}^n + x_{p53}^n)} - x_{Myc}$$

$$\frac{dx_{p21}}{dt} = \frac{a \cdot (x_{C/EBP\alpha}^n + x_{Gata1/2}^n + x_{TGF-\beta}^n + x_{TNF-\alpha}^n + x_{AP2}^n + x_{p53}^n)}{1 + a \cdot (x_{C/EBP\alpha}^n + x_{Gata1/2}^n + x_{TGF-\beta}^n + x_{TNF-\alpha}^n + x_{AP2}^n + x_{p53}^n)} \times \frac{1}{1 + a \cdot (x_{Myc}^n + x_{Hes1}^n + x_{Akt}^n)} - x_{p21}$$

$$\frac{dx_{p27}}{dt} = \frac{a\cdot(x^n_{Gata1/2}+x^n_{Stat1}+x^n_{TGF-\beta}+x^n_{PTEN})}{1+a\cdot(x^n_{Gata1/2}+x^n_{Stat1}+x^n_{TGF-\beta}+x^n_{PTEN})}\times\frac{1}{1+a\cdot(x^n_{Myc}+x^n_{Hes1}+x^n_{Akt})}-x_{p27}$$

$$\frac{dx_{pRb}}{dt} = \frac{a\cdot(x^n_{Cdk2}+x^n_{Cdk6}+x^n_{Cyclin\ D}+x^n_{Cyclin\ E})}{1+a\cdot(x^n_{Cdk2}+x^n_{Cdk6}+x^n_{Cyclin\ D}+x^n_{Cyclin\ E})}-x_{pRb}$$

$$\frac{dx_{Ras}}{dt} = \frac{a\cdot(x^n_{IL-6}+x^n_{EGFR}+x^n_{FGFR2}+x^n_{VEGF})}{1+a\cdot(x^n_{IL-6}+x^n_{EGFR}+x^n_{FGFR2}+x^n_{VEGF})}\times\frac{1}{1+a\cdot(x^n_{p53})}-x_{Ras}$$

$$\frac{dx_{Bad}}{dt} = \frac{a\cdot(x^n_{TNF-\alpha}+x^n_{p53})}{1+a\cdot(x^n_{TNF-\alpha}+x^n_{p53})}\times\frac{1}{1+a\cdot(x^n_{p21}+x^n_{NF-\kappa B}+x^n_{Akt}+x^n_{MAPK})}-x_{Bad}$$

$$\frac{dx_{Bax}}{dt} = \frac{a\cdot(x^n_{Bim}+x^n_{p53})}{1+a\cdot(x^n_{Bim}+x^n_{p53})}\times\frac{1}{1+a\cdot(x^n_{Bcl-2}+x^n_{Sox4}+x^n_{Akt})}-x_{Bax}$$

$$\frac{dx_{Bcl-2}}{dt} = \frac{a\cdot(x^n_{NF-\kappa B}+x^n_{VEGF}+x^n_{Integrin})}{1+a\cdot(x^n_{NF-\kappa B}+x^n_{VEGF}+x^n_{Integrin})}\times\frac{1}{1+a\cdot(x^n_{Caspase\ 3}+x^n_{TGF-\beta}+x^n_{p53})}-x_{Bcl-2}$$

$$\frac{dx_{Bcl-xL}}{dt} = \frac{a\cdot(x^n_{NF-\kappa B}+x^n_{Stat5}+x^n_{EGFR})}{1+a\cdot(x^n_{NF-\kappa B}+x^n_{Stat5}+x^n_{EGFR})}\times\frac{1}{1+a\cdot(x^n_{Caspase\ 3})}-x_{Bcl-xL}$$

$$\frac{dx_{Bim}}{dt} = \frac{1}{1+a\times(x^n_{Akt}+x^n_{MAPK})}-x_{Bim}$$

$$\frac{dx_{Caspase\ 3}}{dt} = \frac{a\cdot(x^n_{Bad}+x^n_{Caspase\ 8}+x^n_{Cytochrome\ C}+x^n_{Fas}+x^n_{TNF-\alpha})}{1+a\cdot(x^n_{Bad}+x^n_{Caspase\ 8}+x^n_{Cytochrome\ C}+x^n_{Fas}+x^n_{TNF-\alpha})}\times\frac{1}{1+a\cdot(x^n_{XIAP}+x^n_{NF-\kappa B})}-x_{Caspase\ 3}$$

$$\frac{dx_{Caspase\ 8}}{dt} = \frac{a\cdot(x^n_{Fas}+x^n_{TNF-\alpha})}{1+a\cdot(x^n_{Fas}+x^n_{TNF-\alpha})}\times\frac{1}{1+a\cdot(x^n_{NF-\kappa B})}-x_{Caspase\ 8}$$

$$\frac{dx_{Cytochrome\ C}}{dt} = \frac{a\cdot(x^n_{Bad}+x^n_{Bax}+x^n_{Caspase\ 3})}{1+a\cdot(x^n_{Bad}+x^n_{Bax}+x^n_{Caspase\ 3})}\times\frac{1}{1+a\cdot(x^n_{p21}+x^n_{Bcl-2}+x^n_{Bcl-xL})}-x_{Cytochrome\ C}$$

$$\frac{dx_{Fas}}{dt} = \frac{a\cdot(x^n_{TNF-\alpha})}{1+a\cdot(x^n_{TNF-\alpha})}\times\frac{1}{1+a\cdot(x^n_{Ras})}-x_{Fas}$$

$$\frac{dx_{XIAP}}{dt} = \frac{a\cdot(x^n_{NF-\kappa B}+x^n_{Akt}+x^n_{MAPK}+x^n_{Integrin}+x^n_{RARs})}{1+a\cdot(x^n_{NF-\kappa B}+x^n_{Akt}+x^n_{MAPK}+x^n_{Integrin}+x^n_{RARs})}\times\frac{1}{1+a\cdot(x^n_{Caspase\ 3})}-x_{XIAP}$$

$$\frac{dx_{C/EBP\alpha}}{dt} = \frac{a\cdot(x^n_{C/EBP\beta}+x^n_{Runx1}+x^n_{NOG})}{1+a\cdot(x^n_{C/EBP\beta}+x^n_{Runx1}+x^n_{NOG})}\times\frac{1}{1+a\cdot(x^n_{IL-1}+x^n_{Stat5}+x^n_{HIF}+x^n_{Notch}+x^n_{SHH}+x^n_{NR2F2})}-x_{C/EBP\alpha}$$

$$\frac{dx_{C/EBP\beta}}{dt} = \frac{a\cdot(x^n_{IL-1}+x^n_{AP2}+x^n_{MAPK})}{1+a\cdot(x^n_{IL-1}+x^n_{AP2}+x^n_{MAPK})}\times\frac{1}{1+a\cdot(x^n_{Hoxa3}+x^n_{TGF-\beta}+x^n_{Akt})}-x_{C/EBP\beta}$$

$$\frac{dx_{Cdx2}}{dt} = \frac{a\cdot(x^n_{Gata1/2}+x^n_{Sox2}+x^n_{BMP}+x^n_{c-Jun}+x^n_{MAPK})}{1+a\cdot(x^n_{Gata1/2}+x^n_{Sox2}+x^n_{BMP}+x^n_{c-Jun}+x^n_{MAPK})}\times\frac{1}{1+a\cdot(x^n_{C/EBP\beta}+x^n_{Sox9}+x^n_{Stat3}+x^n_{TGF-\beta})}-x_{Cdx2}$$

$$\frac{dx_{Gata1/2}}{dt} = \frac{a\cdot(x^n_{Cdx2}+x^n_{Stat3}+x^n_{Notch})}{1+a\cdot(x^n_{Cdx2}+x^n_{Stat3}+x^n_{Notch})}\times\frac{1}{1+a\cdot(x^n_{Hes1}+x^n_{Hoxa10}+x^n_{Pu.1}+x^n_{VEGF}+x^n_{c-Jun})}-x_{Gata1/2}$$

$$\frac{dx_{Gata4/6}}{dt} = \frac{a\cdot(x^n_{Sox7}+x^n_{PKA}+x^n_{Wnt})}{1+a\cdot(x^n_{Sox7}+x^n_{PKA}+x^n_{Wnt})}\times\frac{1}{1+a\cdot(x^n_{Hey2}+x^n_{PRDM14}+x^n_{Sox2}+x^n_{c-Jun})}-x_{Gata4/6}$$

$$\frac{dx_{Hes1}}{dt} = \frac{a\cdot(x^n_{TGF-\beta}+x^n_{Notch})}{1+a\cdot(x^n_{TGF-\beta}+x^n_{Notch})}-x_{Hes1}$$

$$\frac{dx_{Hey2}}{dt} = \frac{a\cdot(x^n_{Notch})}{1+a\cdot(x^n_{Notch})}-x_{Hey2}$$

$$\frac{dx_{Hoxa10}}{dt} = \frac{a\cdot(x^n_{Hoxa9}+x^n_{Stat5}+x^n_{BMP}+x^n_{Wnt})}{1+a\cdot(x^n_{Hoxa9}+x^n_{Stat5}+x^n_{BMP}+x^n_{Wnt})}\times\frac{1}{1+a\cdot(x^n_{Cdx2})}-x_{Hoxa10}$$

$$\frac{dx_{Hoxa3}}{dt} = \frac{a\cdot(x^n_{c-Jun}+x^n_{Wnt}+x^n_{RARs})}{1+a\cdot(x^n_{c-Jun}+x^n_{Wnt}+x^n_{RARs})}\times\frac{1}{1+a\cdot(x^n_{Stat3})}-x_{Hoxa3}$$

$$\frac{dx_{Hoxa5}}{dt} = \frac{a\cdot(x^n_{Hoxa10}+x^n_{RARs})}{1+a\cdot(x^n_{Hoxa10}+x^n_{RARs})}\times\frac{1}{1+a\cdot(x^n_{Runx1})}-x_{Hoxa5}$$

$$\frac{dx_{Hoxa9}}{dt} = \frac{a\cdot(x^n_{C/EBP\alpha}+x^n_{Pu.1}+x^n_{Notch})}{1+a\cdot(x^n_{C/EBP\alpha}+x^n_{Pu.1}+x^n_{Notch})}\times\frac{1}{1+a\cdot(x^n_{NF-\kappa B})}-x_{Hoxa9}$$

$$\frac{dx_{PGC-1}}{dt} = \frac{a\cdot(x^n_{C/EBP\beta}+x^n_{IFN-\gamma})}{1+a\cdot(x^n_{C/EBP\beta}+x^n_{IFN-\gamma})}\times\frac{1}{1+a\cdot(x^n_{p53})}-x_{PGC-1}$$

$$\frac{dx_{PPAR\gamma}}{dt} = \frac{a\cdot(x^n_{C/EBP\alpha}+x^n_{C/EBP\beta})}{1+a\cdot(x^n_{C/EBP\alpha}+x^n_{C/EBP\beta})}\times\frac{1}{1+a\cdot(x^n_{TGF-\beta}+x^n_{TNF-\alpha}+x^n_{NR2F2}+x^n_{RARs})}-x_{PPAR\gamma}$$

$$\frac{dx_{PRDM14}}{dt} = \frac{a\cdot(x^n_{Stat3})}{1+a\cdot(x^n_{Stat3})}-x_{PRDM14}$$

$$\frac{dx_{Pu.1}}{dt} = \frac{a\cdot(x^n_{C/EBP\alpha}+x^n_{Runx1})}{1+a\cdot(x^n_{C/EBP\alpha}+x^n_{Runx1})}\times\frac{1}{1+a\cdot(x^n_{Gata1/2}+x^n_{Sox4})}-x_{Pu.1}$$

$$\frac{dx_{Runx1}}{dt} = \frac{a\cdot(x^n_{Cdx2}+x^n_{Gata1/2}+x^n_{Sox2}+x^n_{c-Jun}+x^n_{Notch})}{1+a\cdot(x^n_{Cdx2}+x^n_{Gata1/2}+x^n_{Sox2}+x^n_{c-Jun}+x^n_{Notch})}\times\frac{1}{1+a\cdot(x^n_{Hoxa3}+x^n_{Stat5})}-x_{Runx1}$$

$$\frac{dx_{Runx2}}{dt} = \frac{a\cdot(x^n_{Hoxa10}+x^n_{Runx1}+x^n_{TGF-\beta}+x^n_{BMP})}{1+a\cdot(x^n_{Hoxa10}+x^n_{Runx1}+x^n_{TGF-\beta}+x^n_{BMP})}\times\frac{1}{1+a\cdot(x^n_{C/EBP\beta}+x^n_{Stat5}+x^n_{Notch}+x^n_{NR2F2})}-x_{Runx2}$$

$$\frac{dx_{Sox2}}{dt} = \frac{a\cdot(x^n_{Stat3}+x^n_{FGFR2}+x^n_{AP2}+x^n_{Wnt})}{1+a\cdot(x^n_{Stat3}+x^n_{FGFR2}+x^n_{AP2}+x^n_{Wnt})}\times\frac{1}{1+a\cdot(x^n_{p21})}-x_{Sox2}$$

$$\frac{dx_{Sox4}}{dt} = \frac{a\cdot(x^n_{Sox7}+x^n_{Stat5}+x^n_{TGF-\beta}+x^n_{Wnt})}{1+a\cdot(x^n_{Sox7}+x^n_{Stat5}+x^n_{TGF-\beta}+x^n_{Wnt})}\times\frac{1}{1+a\cdot(x^n_{C/EBP\alpha})}-x_{Sox4}$$

$$\frac{dx_{Sox7}}{dt} = \frac{a\cdot(x^n_{BMP}+x^n_{c-Jun})}{1+a\cdot(x^n_{BMP}+x^n_{c-Jun})}\times\frac{1}{1+a\cdot(x^n_{PRDM14})}-x_{Sox7}$$

$$\frac{dx_{Sox9}}{dt} = \frac{a\cdot(x^n_{TGF-\beta}+x^n_{BMP}+x^n_{AP2}+x^n_{PKA}+x^n_{SHH}+x^n_{Wnt})}{1+a\cdot(x^n_{TGF-\beta}+x^n_{BMP}+x^n_{AP2}+x^n_{PKA}+x^n_{SHH}+x^n_{Wnt})}\times\frac{1}{1+a\cdot(x^n_{IL-1}+x^n_{NF-\kappa B}+x^n_{c-Jun}+x^n_{RARs})}-x_{Sox9}$$

$$\frac{dx_{IFN-\gamma}}{dt} = \frac{a\cdot(x^n_{Runx1}+x^n_{NF-\kappa B}+x^n_{Stat1}+x^n_{OPN})}{1+a\cdot(x^n_{Runx1}+x^n_{NF-\kappa B}+x^n_{Stat1}+x^n_{OPN})}-x_{IFN-\gamma}$$

$$\frac{dx_{IL-1}}{dt} = \frac{a\cdot(x^n_{C/EBP\beta}+x^n_{NF-\kappa B}+x^n_{Stat1}+x^n_{OPN})}{1+a\cdot(x^n_{C/EBP\beta}+x^n_{NF-\kappa B}+x^n_{Stat1}+x^n_{OPN})}\times\frac{1}{1+a\cdot(x^n_{C/EBP\alpha}+x^n_{Akt}+x^n_{AP2})}-x_{IL-1}$$

$$\frac{dx_{IL-10}}{dt} = \frac{a\cdot(x^n_{Fas}+x^n_{TNF-\alpha})}{1+a\cdot(x^n_{Fas}+x^n_{TNF-\alpha})}\times\frac{1}{1+a\cdot(x^n_{IL-10})}-x_{IL-10}$$

$$\frac{dx_{IL-3}}{dt} = \frac{a\cdot(x^n_{C/EBP\alpha}+x^n_{Runx1})}{1+a\cdot(x^n_{C/EBP\alpha}+x^n_{Runx1})}-x_{IL-3}$$

$$\frac{dx_{IL-6}}{dt} = \frac{a\cdot(x^n_{C/EBP\beta}+x^n_{Hoxa3}+x^n_{NGAL})}{1+a\cdot(x^n_{C/EBP\beta}+x^n_{Hoxa3}+x^n_{NGAL})}\times\frac{1}{1+a\cdot(x^n_{Sox2}+x^n_{RARs})}-x_{IL-6}$$

$$\frac{dx_{IL-8}}{dt} = \frac{a\cdot(x^n_{NF-\kappa B}+x^n_{Stat1}+x^n_{TGF-\beta}+x^n_{c-Jun})}{1+a\cdot(x^n_{NF-\kappa B}+x^n_{Stat1}+x^n_{TGF-\beta}+x^n_{c-Jun})}\times\frac{1}{1+a\cdot(x^n_{Stat3})}-x_{IL-8}$$

$$\frac{dx_{i\kappa B}}{dt} = \frac{a\cdot(x^n_{NF-\kappa B}+x^n_{TGF-\beta})}{1+a\cdot(x^n_{NF-\kappa B}+x^n_{TGF-\beta})}\times\frac{1}{1+a\cdot(x^n_{Fas}+x^n_{TNF-\alpha}+x^n_{EGF}+x^n_{Akt})}-x_{i\kappa B}$$

$$\frac{dx_{NF-\kappa B}}{dt} = \frac{a\cdot(x^n_{C/EBP\beta}+x^n_{IFN-\gamma}+x^n_{IL-1}+x^n_{TNF-\alpha})}{1+a\cdot(x^n_{C/EBP\beta}+x^n_{IFN-\gamma}+x^n_{IL-1}+x^n_{TNF-\alpha})}\times\frac{1}{1+a\cdot(x^n_{Cdx2}+x^n_{PPAR\gamma}+x^n_{Sox2}+x^n_{i\kappa B})}-x_{NF-\kappa B}$$

$$\frac{dx_{Stat1}}{dt} = \frac{a\cdot(x^n_{IFN-\gamma})}{1+a\cdot(x^n_{IFN-\gamma})}\times\frac{1}{1+a\cdot(x^n_{TGF-\beta}+x^n_{OPN})}-x_{Stat1}$$

$$\frac{dx_{Stat3}}{dt} = \frac{a\cdot(x^n_{IL-6}+x^n_{IL-8}+x^n_{FGFR2}+x^n_{G-CSFR}+x^n_{VEGF}+x^n_{OPN})}{1+a\cdot(x^n_{IL-6}+x^n_{IL-8}+x^n_{FGFR2}+x^n_{G-CSFR}+x^n_{VEGF}+x^n_{OPN})}$$
$$\times\frac{1}{1+a\cdot(x^n_{Gata1/2}+x^n_{PPAR\gamma}+x^n_{PTEN}+x^n_{RARs})}-x_{Stat3}$$

$$\frac{dx_{Stat5}}{dt} = \frac{a\cdot(x^n_{IL-3}+x^n_{FGFR2}+x^n_{G-CSFR}+x^n_{VEGF})}{1+a\cdot(x^n_{IL-3}+x^n_{FGFR2}+x^n_{G-CSFR}+x^n_{VEGF})}\times\frac{1}{1+a\cdot(x^n_{Runx1}+x^n_{Runx2})}-x_{Stat5}$$

$$\frac{dx_{TGF-\beta}}{dt} = \frac{a\cdot(x^n_{Hoxa10}+x^n_{Hoxa3}+x^n_{Sox7}+x^n_{Stat3}+x^n_{TNF-\alpha}+x^n_{p53}+x^n_{OPN})}{1+a\cdot(x^n_{Hoxa10}+x^n_{Hoxa3}+x^n_{Sox7}+x^n_{Stat3}+x^n_{TNF-\alpha}+x^n_{p53}+x^n_{OPN})}$$
$$\times\frac{1}{1+a\cdot(x^n_{NF-\kappa B}+x^n_{Stat1}+x^n_{Stat5}+x^n_{NR2F2}+x^n_{RARs})}-x_{TGF-\beta}$$

$$\frac{dx_{TNF-\alpha}}{dt} = \frac{a\cdot(x^n_{IL-1}+x^n_{NF-\kappa B})}{1+a\cdot(x^n_{IL-1}+x^n_{NF-\kappa B})}\times\frac{1}{1+a\cdot(x^n_{IL-10})}-x_{TNF-\alpha}$$

$$\frac{dx_{BMP}}{dt} = \frac{a\cdot(x^n_{Gata4/6}+x^n_{Runx1}+x^n_{EGFR}+x^n_{FGFR2})}{1+a\cdot(x^n_{Gata4/6}+x^n_{Runx1}+x^n_{EGFR}+x^n_{FGFR2})}\times\frac{1}{1+a\cdot(x^n_{TGF-\beta}+x^n_{NOG}+x^n_{NR2F2})}-x_{BMP}$$

$$\frac{dx_{EGF}}{dt} = \frac{a\cdot(x^n_{HIF})}{1+a\cdot(x^n_{HIF})}-x_{EGF}$$

$$\frac{dx_{EGFR}}{dt} = \frac{a\cdot(x^n_{Myc}+x^n_{Runx1}+x^n_{Sox4}+x^n_{EGF})}{1+a\cdot(x^n_{Myc}+x^n_{Runx1}+x^n_{Sox4}+x^n_{EGF})}\times\frac{1}{1+a\cdot(x^n_{PTEN})}-x_{EGFR}$$

$$\frac{dx_{FGF2}}{dt} = \frac{a\cdot(x^n_{Hoxa10}+x^n_{HSPG2})}{1+a\cdot(x^n_{Hoxa10}+x^n_{HSPG2})}-x_{FGF2}$$

$$\frac{dx_{FGF7}}{dt} = \frac{a\cdot(x^n_{IL-1}+x^n_{HSPG2})}{1+a\cdot(x^n_{IL-1}+x^n_{HSPG2})}-x_{FGF7}$$

$$\frac{dx_{FGFR2}}{dt} = \frac{a\cdot(x^n_{Hoxa10}+x^n_{Runx2}+x^n_{FGF2}+x^n_{FGF7})}{1+a\cdot(x^n_{Hoxa10}+x^n_{Runx2}+x^n_{FGF2}+x^n_{FGF7})}\times\frac{1}{1+a\cdot(x^n_{PRDM14})}-x_{FGFR2}$$

$$\frac{dx_{G-CSFR}}{dt} = \frac{a\cdot(x^n_{C/EBP\alpha}+x^n_{Pu.1}+x^n_{NF-\kappa B})}{1+a\cdot(x^n_{C/EBP\alpha}+x^n_{Pu.1}+x^n_{NF-\kappa B})}-x_{G-CSFR}$$

$$\frac{dx_{VEGF}}{dt} = \frac{a\cdot(x^n_{Gata4/6}+x^n_{PGC-1}+x^n_{Stat3}+x^n_{TGF-\beta}+x^n_{HIF}+x^n_{SHH}+x^n_{NR2F2})}{1+a\cdot(x^n_{Gata4/6}+x^n_{PGC-1}+x^n_{Stat3}+x^n_{TGF-\beta}+x^n_{HIF}+x^n_{SHH}+x^n_{NR2F2})}\times\frac{1}{1+a\cdot(x^n_{Sox9}+x^n_{RARs})}-x_{VEGF}$$

$$\frac{dx_{Akt}}{dt} = \frac{a\cdot(x^n_{Ras}+x^n_{NF-\kappa B}+x^n_{EGF}+x^n_{EGFR}+x^n_{FGFR2}+x^n_{c-Jun}+x^n_{PKA}+x^n_{Notch})}{1+a\cdot(x^n_{Ras}+x^n_{NF-\kappa B}+x^n_{EGF}+x^n_{EGFR}+x^n_{FGFR2}+x^n_{c-Jun}+x^n_{PKA}+x^n_{Notch})}\times\frac{1}{1+a\cdot(x^n_{PTEN})}-x_{Akt}$$

$$\frac{dx_{AP2}}{dt} = \frac{a\cdot(x^n_{C/EBP\beta}+x^n_{NR2F2}+x^n_{RARs})}{1+a\cdot(x^n_{C/EBP\beta}+x^n_{NR2F2}+x^n_{RARs})}-x_{AP2}$$

$$\frac{dx_{Bach1}}{dt} = \frac{a\cdot(x^n_{TGF-\beta}+x^n_{HIF})}{1+a\cdot(x^n_{TGF-\beta}+x^n_{HIF})}-x_{Bach1}$$

$$\frac{dx_{c-Jun}}{dt} = \frac{a\cdot(x^n_{Runx1}+x^n_{TNF-\alpha}+x^n_{EGFR}+x^n_{Akt})}{1+a\cdot(x^n_{Runx1}+x^n_{TNF-\alpha}+x^n_{EGFR}+x^n_{Akt})}\times\frac{1}{1+a\cdot(x^n_{C/EBP\alpha})}-x_{c-Jun}$$

$$\frac{dx_{HIF}}{dt} = \frac{a\cdot(x^n_{Stat3}+x^n_{Akt})}{1+a\cdot(x^n_{Stat3}+x^n_{Akt})}\times\frac{1}{1+a\cdot(x^n_{p53})}-x_{HIF}$$

$$\frac{dx_{MAPK}}{dt} = \frac{a\cdot(x^n_{Ras}+x^n_{PPAR\gamma}+x^n_{IFN-\gamma}+x^n_{NF-\kappa B}+x^n_{EGFR}+x^n_{FGFR2}+x^n_{Integrin}+x^n_{NR2F2})}{1+a\cdot(x^n_{Ras}+x^n_{PPAR\gamma}+x^n_{IFN-\gamma}+x^n_{NF-\kappa B}+x^n_{EGFR}+x^n_{FGFR2}+x^n_{Integrin}+x^n_{NR2F2})}$$
$$\times\frac{1}{1+a\cdot(x^n_{TGF-\beta}+x^n_{PTEN})}-x_{MAPK}$$

$$\frac{dx_{p53}}{dt} = \frac{a\cdot(x^n_{Cdk6}+x^n_{Myc}+x^n_{PPAR\gamma}+x^n_{IFN-\gamma}+x^n_{PTEN}+x^n_{RARs})}{1+a\cdot(x^n_{Cdk6}+x^n_{Myc}+x^n_{PPAR\gamma}+x^n_{IFN-\gamma}+x^n_{PTEN}+x^n_{RARs})}\times\frac{1}{1+a\cdot(x^n_{Sox4}+x^n_{Akt}+x^n_{Bach1})}-x_{p53}$$

$$\frac{dx_{PKA}}{dt} = \frac{a\cdot(x^n_{TGF-\beta}+x^n_{EGFR}+x^n_{Akt})}{1+a\cdot(x^n_{TGF-\beta}+x^n_{EGFR}+x^n_{Akt})}\times\frac{1}{1+a\cdot(x^n_{p53})}-x_{PKA}$$

$$\frac{dx_{PTEN}}{dt} = \frac{a\cdot(x^n_{PPAR\gamma}+x^n_{AP2}+x^n_{p53})}{1+a\cdot(x^n_{PPAR\gamma}+x^n_{AP2}+x^n_{p53})}\times\frac{1}{1+a\cdot(x^n_{NF-\kappa B}+x^n_{Stat3})}-x_{PTEN}$$

$$\frac{dx_{HSPG2}}{dt} = \frac{a\cdot(x^n_{Runx2}+x^n_{TGF-\beta})}{1+a\cdot(x^n_{Runx2}+x^n_{TGF-\beta})}-x_{HSPG2}$$

$$\frac{dx_{Integrin}}{dt} = \frac{a\cdot(x^n_{TNF-\alpha}+x^n_{EGF}+x^n_{VEGF}+x^n_{MAPK})}{1+a\cdot(x^n_{TNF-\alpha}+x^n_{EGF}+x^n_{VEGF}+x^n_{MAPK})}\times\frac{1}{1+a\cdot(x^n_{Myc})}-x_{Integrin}$$

$$\frac{dx_{NGAL}}{dt} = \frac{a\cdot(x^n_{Runx1}+x^n_{IFN-\gamma}+x^n_{TNF-\alpha})}{1+a\cdot(x^n_{Runx1}+x^n_{IFN-\gamma}+x^n_{TNF-\alpha})}-x_{NGAL}$$

$$\frac{dx_{NOG}}{dt} = \frac{a\cdot(x^n_{Sox9}+x^n_{SHH}+x^n_{Wnt})}{1+a\cdot(x^n_{Sox9}+x^n_{SHH}+x^n_{Wnt})}\times\frac{1}{1+a\cdot(x^n_{FGFR2})}-x_{NOG}$$

$$\frac{dx_{Notch}}{dt} = \frac{a\cdot(x^n_{Cdx2}+x^n_{NF-\kappa B}+x^n_{Stat3}+x^n_{FGFR2})}{1+a\cdot(x^n_{Cdx2}+x^n_{NF-\kappa B}+x^n_{Stat3}+x^n_{FGFR2})}\times\frac{1}{1+a\cdot(x^n_{C/EBP\alpha}+x^n_{Hoxa5}+x^n_{BMP}+x^n_{NR2F2})}-x_{Notch}$$

$$\frac{dx_{OPN}}{dt} = \frac{a\cdot(x^n_{Runx1}+x^n_{Runx2}+x^n_{TGF-\beta})}{1+a\cdot(x^n_{Runx1}+x^n_{Runx2}+x^n_{TGF-\beta})}\times\frac{1}{1+a\cdot(x^n_{PGC-1})}-x_{OPN}$$

$$\frac{dx_{SHH}}{dt} = \frac{a\cdot(x^n_{Sox2}+x^n_{TGF-\beta})}{1+a\cdot(x^n_{Sox2}+x^n_{TGF-\beta})}\times\frac{1}{1+a\cdot(x^n_{Cdx2}+x^n_{Hes1}+x^n_{BMP}+x^n_{p53})}-x_{SHH}$$

$$\frac{dx_{Wnt}}{dt} = \frac{a\cdot(x^n_{PGC-1}+x^n_{Sox4}+x^n_{TGF-\beta})}{1+a\cdot(x^n_{PGC-1}+x^n_{Sox4}+x^n_{TGF-\beta})} \times \frac{1}{1+a\cdot(x^n_{Cdx2}+x^n_{Sox2}+x^n_{Sox7}+x^n_{Sox9})} - x_{Wnt}$$

$$\frac{dx_{NR2F2}}{dt} = \frac{a\cdot(x^n_{SHH}+x^n_{Wnt})}{1+a\cdot(x^n_{SHH}+x^n_{Wnt})} - x_{NR2F2}$$

$$\frac{dx_{RARs}}{dt} = \frac{a\cdot(x^n_{PPAR\gamma}+x^n_{AP2})}{1+a\cdot(x^n_{PPAR\gamma}+x^n_{AP2})} \times \frac{1}{1+a\cdot(x^n_{MAPK}+x^n_{NR2F2})} - x_{RARs}$$

C. Attractor, Saddle Points, and Other Unstable Points Obtained from the Endogenous Network Dynamics

From the dynamics of the APL network, we obtained 18 attractors, 32 saddle points, as well as 25 other unstable fixed points shown in Supplementary Table II, VI, and VII separately. The attractors are calculated using equations listed in Sect. II B with parameter $n = 3$ and $a = 10$ by two methods: a) Newtonian iteration method for solving fixed point and b) Euler method for calculating trajectories of differential equations. They generate identical result. The 18 attractors are first obtained while calculating with 60,000 random initial positions. We further run the program with 100,000 and 1,000,000 initial positions, acquiring identical 18 attractors. The 57 unstable points including 32 saddle points are first obtained while calculating with 100,000 random initial positions using Newtonian iteration method. We further run the program with 1,000,000 initial positions, acquiring an identical result.

D. Random Parameter Tests

We performed 4 groups of random parameter tests. In the first two tests (Supplementary Figure 1): 1) we use the equations listed in Sect. II B with uniformly distributed real random numbers $n \in [3, 13]$ and $a \in [n^2+1, 10n^2]$. 50,000 tests show that all 18 attractors have 100% recurrence rate. 2) we use a more general form of equations than those listed in Sect. II B. We allow different coefficients a and b for activation and inhibition separately. We further enable a, b, and n to vary in different equations as a_i, b_i, and n_i, where $i = 1, \ldots, 81$. Under the setting, we generate 10,000 sets (each set has 243 random numbers) of random parameters with $a_i \in [10, 1000]$, $b_i \in [10, 1000]$, and $n_i \in [3, 13]$, put into the equations, and calculate attractors using Euler method. The recurrence rate of each attractor calculated in

Supplementary Table II is above 90%; the functional states discussed in the main text are all about 100%.

In the following two tests (Supplementary Figure 2), we use the equations that enable different n_{ij} for each of the 416 interactions $i, j = 1, \ldots, 81$, where agent j to agent i has an interaction. With uniformly distributed real random numbers $n_{ij} \in [3, 13]$ and $a_{ij} = 5n_{ij}^2$, 50,000 tests show that all 18 attractors have 100% recurrence rate. In the last test, we also allow a_{ij} and n_{ij} are independent. Under this setting, we generate 10,000 sets (each set has $416 \times 2 = 832$ random numbers) of random parameters with $a_{ij} \in [10, 1000]$, and $n_{ij} \in [3, 13]$, put into the equations, and calculate attractors using Euler method. The recurrence rate of each attractor calculated in Supplementary Table II is above 90%; the functional states discussed in the main text are all about 100%.

E. Stability under Different Equation Forms

We included here two different forms of equations to test the structural stability [1] of the dynamical system under study.

1. *Alternative equation (a)*

A formal representation would be:

$$\frac{dx_i}{dt} = \frac{a \cdot (\sum_{u \in act} x_u)^n}{1 + a \cdot (\sum_{u \in act} x_u)^n} \cdot \frac{1}{1 + a \cdot (\sum_{v \in inh} x_v)^n} - x_i \tag{2}$$

where $i = 1, \ldots, 81$, act denotes the set of all nodes that activate node i and inh the set of nodes that inhibit node i. As an example, if there are multiple activations from x_2, x_3 and inhibitions from x_4, x_5 to x_1, we have

$$\frac{dx_1}{dt} = \frac{a \cdot (x_2 + x_3)^n}{1 + a \cdot (x_2 + x_3)^n} \cdot \frac{1}{1 + a \cdot (x_4 + x_5)^n} - x_1 \ . \tag{3}$$

By using *Euler method*, 16 attractors are find with values listed in Supplementary Table IV (a), including all functional attractors discussed in the main text. S6 and S13 are not found.

2. Alternative equation (b)

This form can be expressed as

$$\frac{dx_i}{dt} = \frac{1}{|act|}\left(\sum_{u\in act}\frac{a\cdot x_u^n}{1+a\cdot x_u^n}\right)\cdot\frac{1}{1+b\cdot(\sum_{v\in inh}x_v^n)} - x_i \tag{4}$$

where $i = 1, \ldots, 81$, act denotes the set of all nodes that activate node i and inh the set of nodes that inhibit node i, and $|act|$ means the number of nodes in the set act. For instance, if there are multiple activations from x_2, x_3 and inhibitions from x_4, x_5 to x_1, we have

$$\frac{dx_1}{dt} = \frac{1}{2}\left(\frac{a\cdot x_2^n}{1+a\cdot x_2^n}+\frac{a\cdot x_3^n}{1+a\cdot x_3^n}\right)\cdot\frac{1}{1+b\cdot(x_3^n+x_4^n)} - x_1 \tag{5}$$

Using *Euler method*, 9 attractors including major functional states S1, S4, and S12 discussed in main text are obtained.

F. Comparison with Boolean Dynamics

Boolean dynamics (Boolean network) as a simple but effective tool have been used for the modeling of cellular regulatory networks [2–4] and endogenous molecular-cellular network for cancers [5–7]. As a robustness test of the attractors obtained from differential equations, here we also used Boolean networks with threshold functions to analyze this network. In the model, each node i has two states, $x_i = 1$ and $x_i = 0$, representing an activated state and an inhibited state. The time-evolution of the network is defined as

$$x_i(t+1) = \begin{cases} 1 & \sum_j a_{ij}x_j(t) > 0 \\ 0 & \sum_j a_{ij}x_j(t) <= 0 \end{cases}$$

where $a_{ij} = 1$ representing that node j activates node i, and $a_{ij} = -100$ denoting node j inhibits node i. The inhibition is dominant in our model corresponds to a usual biological situation. We find the attractors obtained from differential equations are also supported by Boolean dynamics as shown in Supplementary Table IV. Note that we treat a periodic attractor (S15) generated by Boolean dynamics as a point attractor with averaging along its trajectory to be comparable with the results in Supplementary Table II.

G. Induced Transitions

In Supplementary Table VIII, we enumerate all possible combinations of selecting 1, 2, or 3 nodes with up-regulation (1) or down-regulation (0), and then follow the trajectory to see which attractor it will reach. The total number of runs are $2C_{81}^{1} = 162$, $2^2C_{81}^{2} = 12960$, and $2^3C_{81}^{3} = 682560$. Due to the stability of an attractor, most perturbations lead back to the original attractor, we list here the rare combinations go to other attractors. More data can be seen from Supplementary File 3.

---

[1] M. W. Hirsch and S. Smale, *Differential equations, dynamical systems, and linear algebra* (Academic Press, San Diego, 1974).

[2] S. Kauffman, C. Peterson, B. Samuelsson, and C. Troein, Proc. Natl Acad. Sci. USA **100**, 14796 (2003).

[3] F. Li, T. Long, Y. Lu, Q. Ouyang, and C. Tang, Proc. Natl Acad. Sci. USA **101**, 4781 (2004).

[4] S. Bornholdt, J. R. Soc. Interface **5**, S85 (2008).

[5] G.-W. Wang, X.-M. Zhu, J.-R. Gu, and P. Ao, Interface Focus **4**, 20130064 (2014).

[6] X. Zhu, R. Yuan, L. Hood, and P. Ao, Prog. Biophys. Mol. Biol. **117**, 30 (2015).

[7] S. Li, X. Zhu, B. Liu, G. Wang, and P. Ao, Oncotarget **6**, 13607 (2015).

III. SUPPLEMENTARY TABLES AND FIGURES

Supplementary Table II. Attractor Obtained from the Endogenous Network Dynamics.

This table has the original data for Fig. 2. It is calculated using equations listed in Sect. II B with parameter $n = 3$ and $a = 10$ by two methods: a) *Newtonian iteration method* for solving fixed point and b) *Euler method* for calculating trajectories of differential equations. They generate identical result. The 18 attractors are first obtained while calculating with 60,000 random initial positions. We further run the program with 100,000 and 1,000,000 initial positions, acquiring identical 18 attractors.

	S1	S2	S3	S4	S5	S6	S7	S8	S9	S10	S11	S12	S13	S14	S15	S16	S17	S18
Recurrence Times: (Tot. 1,000,000)	81849	5266	31019	219726	432715	51293	50577	1604	6299	18422	691	96085	473	1820	212	1103	36	810
Cdk2	0.8981	0.8960	0.8988	0.8857	0.8971	0.8873	0.0160	0.0160	0.8988	0.8981	0.8960	0.0033	0.0000	0.0013	0.0000	0.0160	0.0160	0.8873
Cdk6	0.8977	0.8957	0.9556	0.9544	0.8965	0.8868	0.8605	0.8605	0.9556	0.8977	0.8957	0.0032	0.0000	0.0006	0.0000	0.8605	0.8605	0.8868
Cyclin D	0.9273	0.9334	0.9274	0.9598	0.9615	0.9616	0.9370	0.8962	0.8824	0.8803	0.8861	0.0019	0.0000	0.0000	0.0000	0.9370	0.8962	0.9616
Cyclin E	0.9071	0.9127	0.9113	0.9589	0.9598	0.9598	0.9347	0.8908	0.8588	0.8547	0.8600	0.0000	0.0000	0.0000	0.0000	0.9347	0.8908	0.9598
E2F	0.8581	0.8581	0.8584	0.9437	0.9453	0.9436	0.8659	0.0145	0.0201	0.0203	0.0202	0.0000	0.0000	0.0000	0.0000	0.8659	0.0145	0.9436
Myc	0.1286	0.1285	0.1283	0.9416	0.9598	0.9408	0.1158	0.1136	0.1272	0.1275	0.1275	0.0037	0.0010	0.0017	0.0000	0.1158	0.1136	0.9408
p21	0.0871	0.0872	0.0861	0.0016	0.0002	0.0012	0.0475	0.0475	0.0861	0.0871	0.0872	0.9050	0.9537	0.9551	0.9364	0.0475	0.0475	0.0012
p27	0.0871	0.0016	0.0864	0.0463	0.0452	0.0012	0.0475	0.0475	0.0864	0.0871	0.0016	0.9275	0.9341	0.9531	0.8916	0.0475	0.0475	0.0012
pRb	0.9677	0.9679	0.9693	0.9708	0.9698	0.9694	0.9579	0.9538	0.9669	0.9650	0.9652	0.0000	0.0000	0.0000	0.0000	0.9579	0.9538	0.9694
Ras	0.9429	0.9424	0.9429	0.9442	0.9448	0.9433	0.9458	0.9458	0.9429	0.9429	0.9424	0.8803	0.0016	0.0021	0.0016	0.9458	0.9458	0.9433
Bad	0.0000	0.0000	0.0001	0.0001	0.0000	0.0000	0.0000	0.0000	0.0001	0.0000	0.0000	0.0022	0.0921	0.0920	0.0968	0.0000	0.0000	0.0000
Bax	0.0001	0.0001	0.0002	0.0002	0.0001	0.0001	0.0005	0.0005	0.0002	0.0001	0.0001	0.1159	0.9479	0.9484	0.9479	0.0005	0.0005	0.0001
Bcl-2	0.9437	0.9415	0.9412	0.8763	0.8917	0.8699	0.1144	0.1144	0.9412	0.9437	0.9415	0.0337	0.0000	0.0022	0.0000	0.0707	0.0707	0.1468
Bcl-xL	0.9362	0.9359	0.8895	0.8984	0.9380	0.9372	0.8978	0.8978	0.8895	0.9362	0.9359	0.0454	0.0000	0.0208	0.0000	0.1474	0.1474	0.1552
Bim	0.0532	0.0538	0.0526	0.0535	0.0525	0.0549	0.0960	0.0960	0.0526	0.0532	0.0538	0.9563	1.0000	0.9903	1.0000	0.0960	0.0960	0.0549
Caspase 3	0.0000	0.0000	0.0000	0.0000	0.0000	0.0000	0.0000	0.0000	0.0000	0.0000	0.0000	0.0000	0.0021	0.0021	0.0024	0.7986	0.7985	0.7957
Cytochrome C	0.0000	0.0000	0.0000	0.0000	0.0000	0.0000	0.0000	0.0000	0.0000	0.0000	0.0000	0.0018	0.0925	0.0922	0.0972	0.8063	0.8063	0.7805
Caspase 8	0.0000	0.0000	0.0000	0.0001	0.0001	0.0000	0.0000	0.0000	0.0000	0.0000	0.0000	0.0002	0.0000	0.0004	0.0000	0.0000	0.0000	0.0000
Fas	0.0000	0.0000	0.0000	0.0000	0.0000	0.0000	0.0000	0.0000	0.0000	0.0000	0.0000	0.0000	0.0000	0.0005	0.0000	0.0000	0.0000	0.0000
XIAP	0.9632	0.9629	0.9635	0.9465	0.9476	0.9451	0.9456	0.9457	0.9635	0.9632	0.9629	0.8583	0.8670	0.8635	0.8670	0.1552	0.1552	0.1565
C/EBPα	0.0294	0.0295	0.0411	0.0941	0.0017	0.0018	0.0567	0.0567	0.0411	0.0294	0.0295	0.0347	0.0320	0.0330	0.0150	0.0567	0.0567	0.0018
C/EBPβ	0.0944	0.0943	0.0933	0.0864	0.0869	0.0858	0.0009	0.0009	0.0933	0.0944	0.0943	0.1183	0.1151	0.1168	0.1151	0.0009	0.0009	0.0858
Cdx2	0.1206	0.1213	0.1061	0.1042	0.1183	0.1198	0.0606	0.0606	0.1061	0.1206	0.1213	0.8942	0.8173	0.8310	0.0079	0.0606	0.0606	0.1198
Gata1/2	0.0356	0.0358	0.0277	0.0269	0.0342	0.0348	0.0281	0.0281	0.0277	0.0356	0.0358	0.8453	0.8452	0.8519	0.0000	0.0281	0.0281	0.0348
Gata4/6	0.0372	0.0373	0.0358	0.0374	0.0388	0.0390	0.0301	0.0301	0.0358	0.0372	0.0373	0.8626	0.0000	0.0000	0.0001	0.0301	0.0301	0.0390
Hes1	0.0035	0.0053	0.0185	0.0301	0.0052	0.0238	0.9313	0.9313	0.0185	0.0035	0.0053	0.0060	0.0345	0.0203	0.0190	0.9313	0.9313	0.0238
Hey2	0.0026	0.0026	0.0158	0.0099	0.0021	0.0023	0.8632	0.8632	0.0158	0.0026	0.0026	0.0024	0.0161	0.0172	0.0000	0.8632	0.8632	0.0023
Hoxa10	0.8579	0.8568	0.8570	0.9245	0.9160	0.9127	0.8595	0.8595	0.8570	0.8579	0.8568	0.1093	0.0000	0.0000	0.0001	0.8595	0.8595	0.9127
Hoxa3	0.1124	0.1131	0.0993	0.0968	0.1096	0.1113	0.1089	0.1089	0.0993	0.1124	0.1131	0.8562	0.8670	0.8623	0.8670	0.1089	0.1089	0.1113
Hoxa5	0.8487	0.8480	0.0972	0.0980	0.8669	0.8646	0.0943	0.0943	0.0972	0.8487	0.8480	0.8400	0.8514	0.8466	0.8670	0.0943	0.0943	0.8646
Hoxa9	0.0028	0.0029	0.8666	0.8641	0.0021	0.0023	0.8513	0.8513	0.8666	0.0028	0.0029	0.0028	0.0164	0.0172	0.0000	0.8513	0.8513	0.0023
PGC-1	0.8666	0.0084	0.8900	0.8896	0.8663	0.0064	0.8758	0.8758	0.8900	0.8666	0.0084	0.8485	0.0016	0.0897	0.0016	0.8758	0.8758	0.0064
PPARγ	0.0011	0.0011	0.0012	0.0143	0.0065	0.0061	0.0002	0.0002	0.0012	0.0011	0.0011	0.0024	0.0020	0.0022	0.0020	0.0002	0.0002	0.0061
PRDM14	0.8743	0.8735	0.8895	0.8921	0.8776	0.8756	0.8789	0.8789	0.8895	0.8743	0.8735	0.0012	0.0000	0.0007	0.0000	0.8789	0.8789	0.8756
Pu.1	0.0022	0.0023	0.8868	0.8886	0.0027	0.0029	0.1147	0.1147	0.8868	0.0022	0.0023	0.0015	0.0026	0.0027	0.0000	0.1147	0.1147	0.0029
Runx1	0.1197	0.1204	0.9237	0.9307	0.1275	0.1303	0.9344	0.9344	0.9237	0.1197	0.1204	0.1274	0.1224	0.1245	0.0011	0.9344	0.9344	0.1303
Runx2	0.0599	0.0601	0.1225	0.9426	0.1274	0.1299	0.1306	0.1306	0.1225	0.0599	0.0601	0.8595	0.0352	0.0213	0.0187	0.1306	0.1306	0.1299
Sox2	0.9250	0.9247	0.9301	0.8925	0.8781	0.8761	0.8785	0.8785	0.9301	0.9250	0.9247	0.1114	0.0896	0.0891	0.0941	0.8785	0.8785	0.8761
Sox4	0.8729	0.8722	0.0367	0.0480	0.8646	0.8619	0.8778	0.8778	0.0367	0.8729	0.8722	0.8761	0.0189	0.0032	0.0190	0.8778	0.8778	0.8619
Sox7	0.1163	0.1166	0.1118	0.1164	0.1212	0.1217	0.1154	0.1154	0.1118	0.1163	0.1166	0.8908	0.0001	0.0001	0.0000	0.1154	0.1154	0.1217
Sox9	0.1016	0.1017	0.0967	0.0968	0.0986	0.0987	0.0951	0.0951	0.0967	0.1016	0.1017	0.1331	0.1154	0.1180	0.1154	0.0951	0.0951	0.0987
IFN-γ	0.8671	0.0173	0.9361	0.9352	0.8664	0.0239	0.8913	0.8913	0.9361	0.8671	0.0173	0.8590	0.0185	0.8665	0.0001	0.8913	0.8913	0.0239
IL-1	0.0526	0.0005	0.0525	0.0838	0.0840	0.0008	0.0041	0.0041	0.0525	0.0526	0.0005	0.1172	0.0021	0.1166	0.0020	0.0041	0.0041	0.0008
IL-10	0.0000	0.0000	0.0000	0.0001	0.0001	0.0000	0.0000	0.0000	0.0000	0.0000	0.0000	0.0002	0.0000	0.0005	0.0000	0.0000	0.0000	0.0000
IL-3	0.0171	0.0174	0.8874	0.8897	0.0203	0.0216	0.8908	0.8908	0.8874	0.0171	0.0174	0.0207	0.0183	0.0193	0.0000	0.8908	0.8908	0.0216
IL-6	0.0973	0.0025	0.0987	0.1102	0.1116	0.0026	0.1147	0.1147	0.0987	0.0973	0.0025	0.1323	0.1152	0.1271	0.1152	0.1147	0.1147	0.0026
IL-8	0.1178	0.1131	0.1038	0.1012	0.1148	0.1114	0.1140	0.1140	0.1038	0.1178	0.1131	0.8574	0.0190	0.8656	0.0190	0.1140	0.1140	0.1114
iκB	0.0006	0.0002	0.0007	0.0019	0.0010	0.0012	0.0501	0.0501	0.0007	0.0006	0.0002	0.0149	0.0190	0.0238	0.0190	0.0501	0.0501	0.0012
NF-κB	0.0971	0.0009	0.0984	0.1098	0.1113	0.0008	0.1126	0.1126	0.0984	0.0971	0.0009	0.1059	0.0023	0.1286	0.0149	0.1126	0.1126	0.0008
Stat1	0.8663	0.0001	0.8772	0.8592	0.8640	0.0001	0.1060	0.1060	0.8772	0.8663	0.0001	0.8455	0.0001	0.8639	0.0000	0.1060	0.1060	0.0001
Stat3	0.8859	0.8837	0.9304	0.9387	0.8950	0.8895	0.8987	0.8987	0.9304	0.8859	0.8837	0.0487	0.0007	0.0409	0.0010	0.8987	0.8987	0.8895
Stat5	0.8818	0.8797	0.1076	0.0549	0.8594	0.8521	0.1025	0.1025	0.1076	0.8818	0.8797	0.1216	0.0001	0.0002	0.0000	0.1025	0.1025	0.8521
TGF-β	0.0445	0.0648	0.0651	0.1280	0.0678	0.1302	0.8984	0.8984	0.0651	0.0445	0.0648	0.0712	0.1245	0.0681	0.1245	0.8984	0.8984	0.1302
TNF-α	0.0105	0.0000	0.0109	0.0187	0.0193	0.0000	0.0141	0.0141	0.0109	0.0105	0.0000	0.0272	0.0000	0.0358	0.0000	0.0141	0.0141	0.0000
BMP	0.0644	0.0645	0.0677	0.9244	0.8947	0.8778	0.1144	0.1144	0.0677	0.0644	0.0645	0.9343	0.0177	0.0189	0.0000	0.1144	0.1144	0.8778
EGF	0.8926	0.8926	0.8935	0.8939	0.8932	0.8931	0.8934	0.8934	0.8935	0.8926	0.8926	0.0001	0.0000	0.0000	0.0000	0.8934	0.8934	0.8931
EGFR	0.9205	0.9202	0.9295	0.9593	0.9574	0.9563	0.9567	0.9567	0.9295	0.9205	0.9202	0.1211	0.0020	0.0022	0.0000	0.9567	0.9567	0.9563
FGF2	0.8633	0.8628	0.8629	0.9376	0.8849	0.8838	0.9293	0.9293	0.8629	0.8633	0.8628	0.8660	0.0001	0.0000	0.0001	0.9293	0.9293	0.8838
FGF7	0.0015	0.0000	0.0015	0.8772	0.0060	0.0007	0.8717	0.8717	0.0015	0.0015	0.0000	0.8661	0.0001	0.0156	0.0001	0.8717	0.8717	0.0007
FGFR2	0.1207	0.1210	0.1153	0.1196	0.1206	0.1213	0.1226	0.1226	0.1153	0.1207	0.1210	0.9509	0.0004	0.0001	0.0001	0.1226	0.1226	0.1213
G-CSFR	0.0093	0.0003	0.8747	0.8756	0.0136	0.0000	0.0302	0.0302	0.8747	0.0093	0.0003	0.0121	0.0003	0.0212	0.0001	0.0302	0.0302	0.0000
VEGF	0.9607	0.9544	0.9634	0.9510	0.9475	0.9302	0.9591	0.9591	0.9634	0.9607	0.9544	0.1319	0.0025	0.0015	0.0025	0.9591	0.9591	0.9302
Akt	0.9632	0.9629	0.9676	0.9765	0.9764	0.9763	0.9798	0.9798	0.9676	0.9632	0.9629	0.1306	0.0018	0.0043	0.0000	0.9798	0.9798	0.9763
AP2	0.8676	0.8676	0.8684	0.0065	0.0067	0.0065	0.0002	0.0002	0.8684	0.8676	0.8676	0.8574	0.8673	0.8632	0.8673	0.0002	0.0002	0.0065
Bach1	0.8926	0.8926	0.8936	0.8942	0.8932	0.8933	0.9399	0.9399	0.8936	0.8926	0.8926	0.0037	0.0190	0.0032	0.0190	0.9399	0.9399	0.8933
c-Jun	0.9434	0.9434	0.9608	0.9553	0.9477	0.9476	0.9616	0.9616	0.9608	0.9434	0.9434	0.0574	0.0180	0.0194	0.0000	0.9616	0.9616	0.9476
HIF	0.9402	0.9401	0.9432	0.9446	0.9422	0.9418	0.9429	0.9429	0.9432	0.9402	0.9401	0.0225	0.0000	0.0001	0.0000	0.9429	0.9429	0.9418
MAPK	0.9608	0.9536	0.9642	0.9424	0.9566	0.9245	0.1176	0.1176	0.9642	0.9609	0.9536	0.1329	0.0000	0.0993	0.0000	0.1176	0.1176	0.9245
p53	0.0393	0.0371	0.0549	0.0551	0.0401	0.0394	0.0365	0.0365	0.0549	0.0393	0.0371	0.1225	0.9363	0.9536	0.9363	0.0365	0.0365	0.0394
PKA	0.9431	0.9431	0.9432	0.9462	0.9470	0.9470	0.9617	0.9617	0.9432	0.9431	0.9431	0.0411	0.0021	0.0003	0.0021	0.9617	0.9617	0.9470
PTEN	0.1089	0.1097	0.0957	0.0002	0.0001	0.0001	0.0001	0.0001	0.0957	0.1089	0.1097	0.8523	0.9364	0.9178	0.9364	0.0001	0.0001	0.0001
HSPG2	0.0030	0.0049	0.0207	0.8936	0.0232	0.0422	0.8791	0.8791	0.0207	0.0030	0.0049	0.8640	0.0194	0.0032	0.0190	0.8791	0.8791	0.0422
Integrin	0.9413	0.9408	0.9417	0.1027	0.0976	0.1028	0.9267	0.9275	0.9422	0.9418	0.9412	0.0445	0.0000	0.0101	0.0000	0.9267	0.9275	0.1028
NGAL	0.8673	0.0172	0.9415	0.9420	0.8671	0.0218	0.9384	0.9384	0.9415	0.8673	0.0172	0.8641	0.0181	0.8671	0.0000	0.9384	0.9384	0.0218
NOG	0.8534	0.8532	0.8572	0.0296	0.0364	0.0355	0.0356	0.0356	0.8572	0.8534	0.8532	0.0026	0.0151	0.0162	0.0152	0.0356	0.0356	0.0355
Notch	0.0640	0.0641	0.1171	0.1001	0.0598	0.0616	0.8576	0.8576	0.1171	0.0640	0.0641	0.0623	0.1178	0.1205	0.0000	0.8576	0.8576	0.0616
OPN	0.0026	0.0219	0.1103	0.1172	0.0057	0.0621	0.1217	0.1217	0.1103	0.0026	0.0219	0.1216	0.0367	0.0219	0.0190	0.1217	0.1217	0.0621
SHH	0.8697	0.8695	0.8748	0.0984	0.1065	0.1119	0.1026	0.1026	0.8748	0.8697	0.8695	0.0010	0.0018	0.0007	0.0029	0.1026	0.1026	0.1119
Wnt	0.1037	0.0971	0.0964	0.1075	0.1188	0.1114	0.1222	0.1222	0.0964	0.1037	0.0971	0.0608	0.0029	0.0015	0.0186	0.1222	0.1222	0.1114
NR2F2	0.8682	0.8681	0.8702	0.0215	0.0281	0.0271	0.0282	0.0282	0.8702	0.8682	0.8681	0.0022	0.0000	0.0000	0.0001	0.0282	0.0282	0.0271
RARs	0.0528	0.0535	0.0524	0.0000	0.0000	0.0000	0.0000	0.0000	0.0524	0.0528	0.0535	0.8433	0.8671	0.8571	0.8671	0.0000	0.0000	0.0000

Supplementary Table III. Attractor Obtained with Parameter Scanning $n = \{3, 4, \ldots, 20\}$ and $a = 5n^2$.

	S1	S2	S3	S4	S5	S6	S7	S8	S9	S10	S11	S12	S13	S14	S15	S16	S17	S18
Cdk2	0.9900	0.9900	0.9900	0.9899	0.9899	0.9898	0.0000	0.0000	0.9900	0.9900	0.9900	0.0000	0.0000	0.0000	0.0000	0.0000	0.0000	0.9898
Cdk6	0.9900	0.9900	0.9966	0.9965	0.9899	0.9898	0.9896	0.9896	0.9966	0.9900	0.9900	0.0000	0.0000	0.0000	0.0000	0.9896	0.9896	0.9898
Cyclin D	0.9948	0.9948	0.9948	0.9966	0.9966	0.9966	0.9949	0.9899	0.9898	0.9898	0.9898	0.0000	0.0000	0.0000	0.0000	0.9949	0.9899	0.9966
Cyclin E	0.9948	0.9948	0.9948	0.9966	0.9966	0.9966	0.9948	0.9898	0.9896	0.9896	0.9896	0.0000	0.0000	0.0000	0.0000	0.9948	0.9898	0.9966
E2F	0.9896	0.9896	0.9896	0.9949	0.9949	0.9949	0.9896	0.0000	0.0000	0.0000	0.0000	0.0000	0.0000	0.0000	0.0000	0.9896	0.0000	0.9949
Myc	0.0104	0.0104	0.0104	0.9966	0.9966	0.9966	0.0101	0.0101	0.0104	0.0104	0.0104	0.0000	0.0000	0.0000	0.0000	0.0101	0.0101	0.9966
p21	0.0099	0.0099	0.0099	0.0000	0.0000	0.0000	0.0050	0.0050	0.0099	0.0099	0.0099	0.9948	0.9965	0.9965	0.9948	0.0050	0.0050	0.0000
p27	0.0099	0.0000	0.0099	0.0050	0.0050	0.0000	0.0050	0.0050	0.0099	0.0099	0.0000	0.9965	0.9948	0.9965	0.9898	0.0050	0.0050	0.0000
pRb	0.9974	0.9974	0.9974	0.9974	0.9974	0.9974	0.9966	0.9965	0.9974	0.9974	0.9974	0.0000	0.0000	0.0000	0.0000	0.9966	0.9965	0.9974
Ras	0.9949	0.9949	0.9949	0.9949	0.9949	0.9949	0.9949	0.9949	0.9949	0.9949	0.9949	0.9899	0.0000	0.0000	0.0000	0.9949	0.9949	0.9949
Bad	0.0000	0.0000	0.0000	0.0000	0.0000	0.0000	0.0000	0.0000	0.0000	0.0000	0.0000	0.0000	0.0100	0.0100	0.0101	0.0000	0.0000	0.0000
Bax	0.0000	0.0000	0.0000	0.0000	0.0000	0.0000	0.0000	0.0000	0.0000	0.0000	0.0000	0.0103	0.9950	0.9950	0.9950	0.0000	0.0000	0.0000
Bcl-2	0.9950	0.9949	0.9950	0.9899	0.9899	0.9898	0.0101	0.0101	0.9950	0.9950	0.9949	0.0000	0.0000	0.0000	0.0000	0.0051	0.0051	0.0103
Bcl-xL	0.9948	0.9948	0.9898	0.9899	0.9949	0.9949	0.9899	0.9899	0.9898	0.9948	0.9948	0.0000	0.0000	0.0000	0.0000	0.0103	0.0103	0.0104
Bim	0.0050	0.0050	0.0050	0.0050	0.0050	0.0051	0.0100	0.0100	0.0050	0.0050	0.0050	1.0000	1.0000	1.0000	1.0000	0.0100	0.0100	0.0051
Caspase 3	0.0000	0.0000	0.0000	0.0000	0.0000	0.0000	0.0000	0.0000	0.0000	0.0000	0.0000	0.0000	0.0000	0.0000	0.0000	0.9896	0.9896	0.9896
Cytochrome C	0.0000	0.0000	0.0000	0.0000	0.0000	0.0000	0.0000	0.0000	0.0000	0.0000	0.0000	0.0000	0.0100	0.0100	0.0101	0.9896	0.9896	0.9896
Caspase 8	0.0000	0.0000	0.0000	0.0000	0.0000	0.0000	0.0000	0.0000	0.0000	0.0000	0.0000	0.0000	0.0000	0.0000	0.0000	0.0000	0.0000	0.0000
Fas	0.0000	0.0000	0.0000	0.0000	0.0000	0.0000	0.0000	0.0000	0.0000	0.0000	0.0000	0.0000	0.0000	0.0000	0.0000	0.0000	0.0000	0.0000
XIAP	0.9966	0.9966	0.9966	0.9950	0.9950	0.9949	0.9949	0.9949	0.9966	0.9966	0.9966	0.9896	0.9896	0.9896	0.9896	0.0104	0.0104	0.0104
C/EBPα	0.0026	0.0026	0.0034	0.0100	0.0000	0.0000	0.0051	0.0051	0.0034	0.0026	0.0026	0.0000	0.0000	0.0000	0.0000	0.0051	0.0051	0.0000
C/EBPβ	0.0100	0.0100	0.0100	0.0099	0.0099	0.0099	0.0000	0.0000	0.0100	0.0100	0.0100	0.0103	0.0103	0.0103	0.0103	0.0000	0.0000	0.0099
Cdx2	0.0104	0.0104	0.0101	0.0101	0.0104	0.0104	0.0051	0.0051	0.0101	0.0104	0.0104	0.9948	0.9896	0.9896	0.0000	0.0051	0.0051	0.0104
Gata1/2	0.0034	0.0034	0.0025	0.0025	0.0034	0.0034	0.0025	0.0025	0.0025	0.0034	0.0034	0.9898	0.9896	0.9896	0.0000	0.0025	0.0025	0.0034
Gata4/6	0.0034	0.0034	0.0034	0.0034	0.0034	0.0034	0.0026	0.0026	0.0034	0.0034	0.0034	0.9896	0.0000	0.0000	0.0000	0.0026	0.0026	0.0034
Hes1	0.0000	0.0000	0.0000	0.0000	0.0000	0.0000	0.9948	0.9948	0.0000	0.0000	0.0000	0.0000	0.0000	0.0000	0.0000	0.9948	0.9948	0.0000
Hey2	0.0000	0.0000	0.0000	0.0000	0.0000	0.0000	0.9896	0.9896	0.0000	0.0000	0.0000	0.0000	0.0000	0.0000	0.0000	0.9896	0.9896	0.0000
Hoxa10	0.9896	0.9896	0.9896	0.9948	0.9948	0.9948	0.9896	0.9896	0.9896	0.9896	0.9896	0.0101	0.0000	0.0000	0.0000	0.9896	0.9896	0.9948
Hoxa3	0.0103	0.0103	0.0101	0.0101	0.0103	0.0103	0.0103	0.0103	0.0101	0.0103	0.0103	0.9896	0.9896	0.9896	0.9896	0.0103	0.0103	0.0103
Hoxa5	0.9896	0.9896	0.0100	0.0101	0.9898	0.9898	0.0100	0.0100	0.0100	0.9896	0.9896	0.9896	0.9896	0.9896	0.9896	0.0100	0.0100	0.9898
Hoxa9	0.0000	0.0000	0.9896	0.9896	0.0000	0.0000	0.9896	0.9896	0.9896	0.0000	0.0000	0.0000	0.0000	0.0000	0.0000	0.9896	0.9896	0.0000
PGC-1	0.9896	0.0000	0.9898	0.9898	0.9896	0.0000	0.9896	0.9896	0.9898	0.9896	0.0000	0.9896	0.0000	0.0100	0.0000	0.9896	0.9896	0.0000
PPARγ	0.0000	0.0000	0.0000	0.0000	0.0000	0.0000	0.0000	0.0000	0.0000	0.0000	0.0000	0.0000	0.0000	0.0000	0.0000	0.0000	0.0000	0.0000
PRDM14	0.9896	0.9896	0.9898	0.9898	0.9896	0.9896	0.9896	0.9896	0.9898	0.9896	0.9896	0.0000	0.0000	0.0000	0.0000	0.9896	0.9896	0.9896
Pu.1	0.0000	0.0000	0.9898	0.9898	0.0000	0.0000	0.0103	0.0103	0.9898	0.0000	0.0000	0.0000	0.0000	0.0000	0.0000	0.0103	0.0103	0.0000
Runx1	0.0104	0.0104	0.9949	0.9949	0.0104	0.0104	0.9965	0.9965	0.9949	0.0104	0.0104	0.0104	0.0104	0.0104	0.0000	0.9965	0.9965	0.0104
Runx2	0.0052	0.0052	0.0104	0.9966	0.0104	0.0104	0.0104	0.0104	0.0104	0.0052	0.0052	0.9898	0.0000	0.0000	0.0000	0.0104	0.0104	0.0104
Sox2	0.9948	0.9948	0.9948	0.9898	0.9896	0.9896	0.9896	0.9896	0.9948	0.9948	0.9948	0.0101	0.0100	0.0100	0.0101	0.9896	0.9896	0.9896
Sox4	0.9896	0.9896	0.0000	0.0000	0.9896	0.9896	0.9898	0.9898	0.0000	0.9896	0.9896	0.9896	0.0000	0.0000	0.0000	0.9898	0.9898	0.9896
Sox7	0.0103	0.0103	0.0103	0.0104	0.0104	0.0104	0.0103	0.0103	0.0103	0.0103	0.0103	0.9898	0.0000	0.0000	0.0000	0.0103	0.0103	0.0104
Sox9	0.0101	0.0101	0.0100	0.0100	0.0101	0.0101	0.0100	0.0100	0.0100	0.0101	0.0101	0.0104	0.0103	0.0103	0.0103	0.0100	0.0100	0.0101
IFN-γ	0.9896	0.0000	0.9948	0.9948	0.9896	0.0000	0.9899	0.9899	0.9948	0.9896	0.0000	0.9896	0.0000	0.9896	0.0000	0.9899	0.9899	0.0000
IL-1	0.0051	0.0000	0.0051	0.0099	0.0099	0.0000	0.0000	0.0000	0.0051	0.0051	0.0000	0.0103	0.0000	0.0103	0.0000	0.0000	0.0000	0.0000
IL-10	0.0000	0.0000	0.0000	0.0000	0.0000	0.0000	0.0000	0.0000	0.0000	0.0000	0.0000	0.0000	0.0000	0.0000	0.0000	0.0000	0.0000	0.0000
IL-3	0.0000	0.0000	0.9898	0.9898	0.0000	0.0000	0.9899	0.9899	0.9898	0.0000	0.0000	0.0000	0.0000	0.0000	0.0000	0.9899	0.9899	0.0000
IL-6	0.0101	0.0000	0.0101	0.0103	0.0103	0.0000	0.0103	0.0103	0.0101	0.0101	0.0000	0.0104	0.0103	0.0104	0.0103	0.0103	0.0103	0.0000
IL-8	0.0104	0.0103	0.0101	0.0101	0.0104	0.0103	0.0104	0.0104	0.0101	0.0104	0.0103	0.9896	0.0000	0.9896	0.0000	0.0104	0.0104	0.0103
iκB	0.0000	0.0000	0.0000	0.0000	0.0000	0.0000	0.0051	0.0051	0.0000	0.0000	0.0000	0.0000	0.0000	0.0000	0.0000	0.0051	0.0051	0.0000
NF-κB	0.0101	0.0000	0.0101	0.0103	0.0103	0.0000	0.0103	0.0103	0.0101	0.0101	0.0000	0.0101	0.0000	0.0103	0.0000	0.0103	0.0103	0.0000
Stat1	0.9896	0.0000	0.9898	0.9898	0.9896	0.0000	0.0101	0.0101	0.9898	0.9896	0.0000	0.9896	0.0000	0.9896	0.0000	0.0101	0.0101	0.0000
Stat3	0.9900	0.9900	0.9949	0.9949	0.9899	0.9898	0.9900	0.9900	0.9949	0.9900	0.9900	0.0035	0.0000	0.0034	0.0000	0.9900	0.9900	0.9898
Stat5	0.9900	0.9900	0.0101	0.0051	0.9899	0.9898	0.0100	0.0100	0.0101	0.9900	0.9900	0.0103	0.0000	0.0000	0.0000	0.0100	0.0100	0.9898
TGF-β	0.0035	0.0052	0.0052	0.0104	0.0052	0.0104	0.9948	0.9948	0.0052	0.0035	0.0052	0.0052	0.0104	0.0052	0.0104	0.9948	0.9948	0.0104
TNF-α	0.0000	0.0000	0.0000	0.0000	0.0000	0.0000	0.0000	0.0000	0.0000	0.0000	0.0000	0.0000	0.0000	0.0000	0.0000	0.0000	0.0000	0.0000
BMP	0.0052	0.0052	0.0052	0.9949	0.9899	0.9899	0.0101	0.0101	0.0052	0.0052	0.0052	0.9948	0.0000	0.0000	0.0000	0.0101	0.0101	0.9899
EGF	0.9898	0.9898	0.9898	0.9898	0.9898	0.9898	0.9898	0.9898	0.9898	0.9898	0.9898	0.0000	0.0000	0.0000	0.0000	0.9898	0.9898	0.9898
EGFR	0.9948	0.9948	0.9948	0.9966	0.9965	0.9965	0.9965	0.9965	0.9948	0.9948	0.9948	0.0103	0.0000	0.0000	0.0000	0.9965	0.9965	0.9965
FGF2	0.9896	0.9896	0.9896	0.9948	0.9898	0.9898	0.9948	0.9948	0.9896	0.9896	0.9896	0.9896	0.0000	0.0000	0.0000	0.9948	0.9948	0.9898
FGF7	0.0000	0.0000	0.0000	0.9896	0.0000	0.0000	0.9896	0.9896	0.0000	0.0000	0.0000	0.9896	0.0000	0.0000	0.0000	0.9896	0.9896	0.0000
FGFR2	0.0104	0.0104	0.0104	0.0104	0.0104	0.0104	0.0104	0.0104	0.0104	0.0104	0.0104	0.9965	0.0000	0.0000	0.0000	0.0104	0.0104	0.0104
G-CSFR	0.0000	0.0000	0.9896	0.9896	0.0000	0.0000	0.0000	0.0000	0.9896	0.0000	0.0000	0.0000	0.0000	0.0000	0.0000	0.0000	0.0000	0.0000
VEGF	0.9979	0.9974	0.9979	0.9966	0.9965	0.9948	0.9974	0.9974	0.9979	0.9979	0.9974	0.0104	0.0000	0.0000	0.0000	0.9974	0.9974	0.9948
Akt	0.9979	0.9979	0.9979	0.9979	0.9979	0.9979	0.9983	0.9983	0.9979	0.9979	0.9979	0.0104	0.0000	0.0000	0.0000	0.9983	0.9983	0.9979
AP2	0.9896	0.9896	0.9896	0.0000	0.0000	0.0000	0.0000	0.0000	0.9896	0.9896	0.9896	0.9896	0.9896	0.9896	0.9896	0.0000	0.0000	0.0000
Bach1	0.9898	0.9898	0.9898	0.9898	0.9898	0.9898	0.9949	0.9949	0.9898	0.9898	0.9898	0.0000	0.0000	0.0000	0.0000	0.9949	0.9949	0.9898
c-Jun	0.9949	0.9949	0.9966	0.9966	0.9950	0.9950	0.9966	0.9966	0.9966	0.9949	0.9949	0.0000	0.0000	0.0000	0.0000	0.9966	0.9966	0.9950
HIF	0.9949	0.9949	0.9949	0.9949	0.9949	0.9949	0.9949	0.9949	0.9949	0.9949	0.9949	0.0000	0.0000	0.0000	0.0000	0.9949	0.9949	0.9949
MAPK	0.9979	0.9974	0.9979	0.9966	0.9966	0.9949	0.0101	0.0101	0.9979	0.9979	0.9974	0.0104	0.0000	0.0101	0.0000	0.0101	0.0101	0.9949
p53	0.0034	0.0034	0.0051	0.0051	0.0034	0.0034	0.0034	0.0034	0.0051	0.0034	0.0034	0.0104	0.9948	0.9965	0.9948	0.0034	0.0034	0.0034
PKA	0.9949	0.9949	0.9949	0.9950	0.9950	0.9950	0.9966	0.9966	0.9949	0.9949	0.9949	0.0000	0.0000	0.0000	0.0000	0.9966	0.9966	0.9950
PTEN	0.0103	0.0103	0.0101	0.0000	0.0000	0.0000	0.0000	0.0000	0.0101	0.0103	0.0103	0.9896	0.9948	0.9948	0.9948	0.0000	0.0000	0.0000
HSPG2	0.0000	0.0000	0.0000	0.9899	0.0000	0.0000	0.9898	0.9898	0.0000	0.0000	0.0000	0.9896	0.0000	0.0000	0.0000	0.9898	0.9898	0.0000
Integrin	0.9966	0.9966	0.9966	0.0100	0.0100	0.0100	0.9949	0.9949	0.9966	0.9966	0.9966	0.0000	0.0000	0.0000	0.0000	0.9949	0.9949	0.0100
NGAL	0.9896	0.0000	0.9949	0.9949	0.9896	0.0000	0.9949	0.9949	0.9949	0.9896	0.0000	0.9896	0.0000	0.9896	0.0000	0.9949	0.9949	0.0000
NOG	0.9896	0.9896	0.9896	0.0000	0.0000	0.0000	0.0000	0.0000	0.9896	0.9896	0.9896	0.0000	0.0000	0.0000	0.0000	0.0000	0.0000	0.0000
Notch	0.0052	0.0052	0.0103	0.0101	0.0052	0.0052	0.9896	0.9896	0.0103	0.0052	0.0052	0.0051	0.0103	0.0103	0.0000	0.9896	0.9896	0.0052
OPN	0.0000	0.0000	0.0103	0.0104	0.0000	0.0000	0.0104	0.0104	0.0103	0.0000	0.0000	0.0103	0.0000	0.0000	0.0000	0.0104	0.0104	0.0000
SHH	0.9898	0.9898	0.9898	0.0100	0.0103	0.0103	0.0101	0.0101	0.9898	0.9898	0.9898	0.0000	0.0000	0.0000	0.0000	0.0101	0.0101	0.0103
Wnt	0.0101	0.0101	0.0101	0.0103	0.0104	0.0103	0.0104	0.0104	0.0101	0.0101	0.0101	0.0051	0.0000	0.0000	0.0000	0.0104	0.0104	0.0103
NR2F2	0.9896	0.9896	0.9896	0.0000	0.0000	0.0000	0.0000	0.0000	0.9896	0.9896	0.9896	0.0000	0.0000	0.0000	0.0000	0.0000	0.0000	0.0000
RARs	0.0051	0.0051	0.0051	0.0000	0.0000	0.0000	0.0000	0.0000	0.0051	0.0051	0.0051	0.9896	0.9896	0.9896	0.9896	0.0000	0.0000	0.0000

Results with different n are almost identical to the presented one here with $n = 5$ and $a = 100$ as well that in Supplementary Table II. Attractors for 14 additional parameter settings are included in Supplementary File 2.

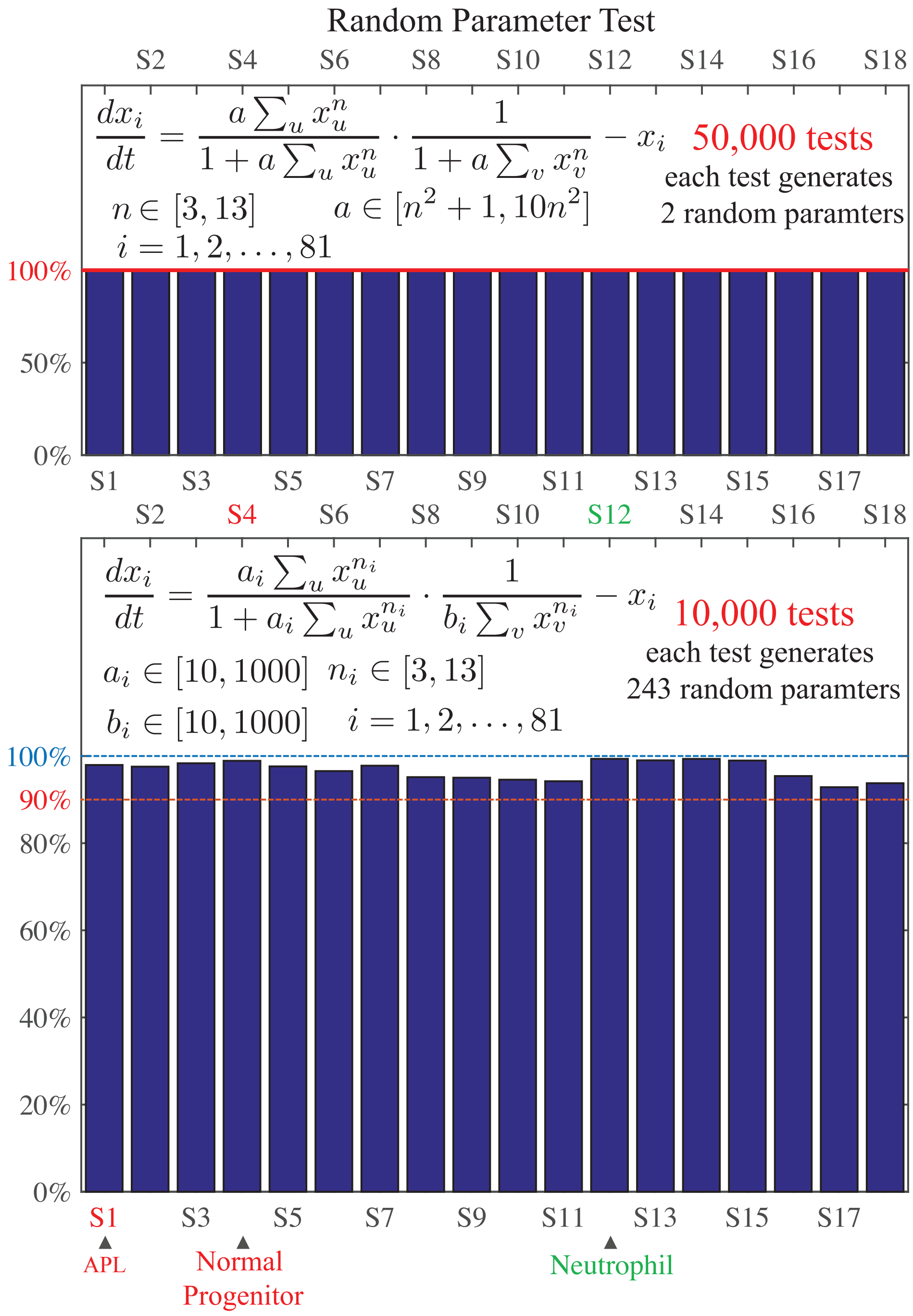

Supplementary Figure 1. Recurrence Rate of Attractors under Random Parameters. In the first test, we use the equations listed in Sect. II B with uniformly distributed real random numbers $n \in [3, 13]$ and $a \in [n^2+1, 10n^2]$. 50,000 tests show that all 18 attractors have 100% recurrence rate. In the second test, we use a more general form of equations than those listed in Sect. II B. We allow different coefficients a and b for activation and inhibition separately. We further enable a, b, and n to vary in different equations as a_i, b_i, and n_i, where $i = 1, \ldots, 81$. Under the setting, we generate 10,000 sets (each set has 243 random numbers) of random parameters with $a_i \in [10, 1000]$, $b_i \in [10, 1000]$, and $n_i \in [3, 13]$, put into the equations, and calculate attractors using Euler method. The recurrence rate of each attractor calculated in Supplementary Table II are shown here: the rates for all attractors are above 90%; the functional states discussed in the main text are all about 100%. Note that the $\sum_u$ means summation over all nodes activating node i and the $\sum_v$ means summation over all nodes inhibiting node i.

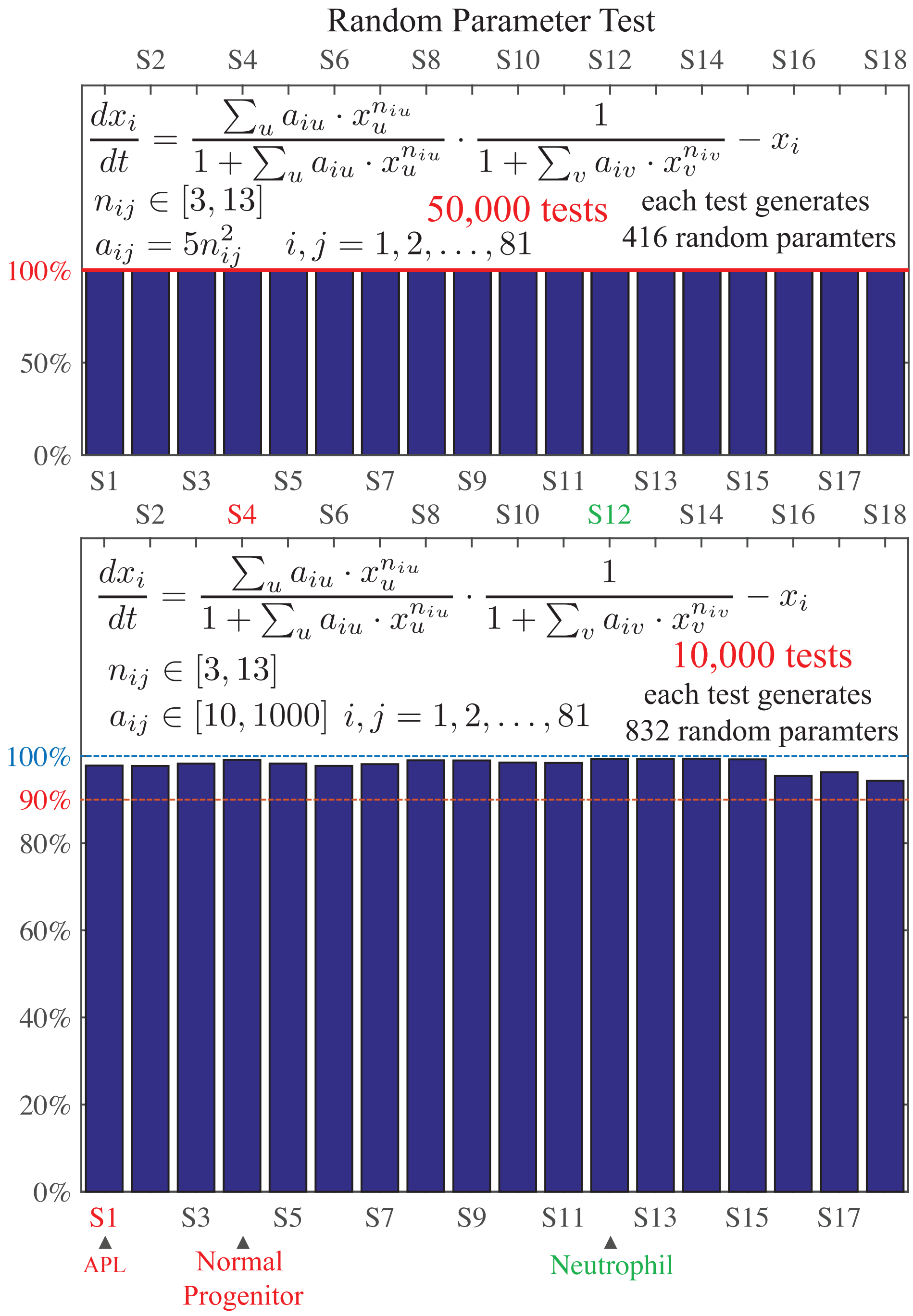

Supplementary Figure 2. Recurrence Rate of Attractors under Random Parameters. In the first test, we use the equations that enable different n_{ij} for each of the 416 interactions $i, j = 1, \ldots, 81$, where agent j to agent i has an interaction. With uniformly distributed real random numbers $n_{ij} \in [3, 13]$ and $a_{ij} = 5n_{ij}^2$, 50,000 tests show that all 18 attractors have 100% recurrence rate. In the second test, we also allow a_{ij} and n_{ij} are independent. Under the setting, we generate 10,000 sets (each set has $416 \times 2 = 832$ random numbers) of random parameters with $a_{ij} \in [10, 1000]$, and $n_{ij} \in [3, 13]$, put into the equations, and calculate attractors using Euler method. The recurrence rate of each attractor calculated in Supplementary Table II are shown here: the rates for all attractors are above 90%; the functional states discussed in the main text are all about 100%. Note that the $\sum_u$ means summation over all nodes activating node i and the $\sum_v$ means summation over all nodes inhibiting node i.

Supplementary Table IV. Recurrence of Attractors Using Different Forms of Equations. Alternative Equation (a): 16 attractors are find with values listed below, including all functional attractors discussed in the main text. S6 and S13 are not found.

	S1	S2	S3	S4	S5	S7	S8	S9	S10	S11	S12	S14	S15	S16	S17	S18
Cdk2	0.9521	0.9518	0.9520	0.9512	0.9522	0.0039	0.0039	0.9520	0.9521	0.9518	0.0002	0.0001	0.0000	0.0039	0.0039	0.9522
Cdk6	0.9521	0.9518	0.9979	0.9980	0.9522	0.9594	0.9594	0.9979	0.9521	0.9518	0.0002	0.0000	0.0000	0.9594	0.9594	0.9522
Cyclin D	0.9924	0.9938	0.9919	0.9981	0.9981	0.9939	0.9597	0.9567	0.9573	0.9584	0.0001	0.0000	0.0000	0.9939	0.9597	0.9981
Cyclin E	0.9583	0.9792	0.9612	0.9980	0.9980	0.9938	0.9592	0.9226	0.9200	0.9400	0.0000	0.0000	0.0000	0.9938	0.9592	0.9980
E2F	0.9521	0.9519	0.9519	0.9937	0.9937	0.9539	0.0048	0.0035	0.0039	0.0037	0.0000	0.0000	0.0000	0.9539	0.0048	0.9937
Myc	0.0541	0.0533	0.0528	0.9948	0.9983	0.0576	0.0574	0.0527	0.0540	0.0532	0.0003	0.0001	0.0000	0.0576	0.0574	0.9983
p21	0.0390	0.0392	0.0389	0.0001	0.0000	0.0054	0.0054	0.0389	0.0390	0.0392	0.9892	0.9981	0.9938	0.0054	0.0054	0.0000
p27	0.0392	0.0002	0.0390	0.0058	0.0059	0.0055	0.0055	0.0390	0.0392	0.0002	0.9943	0.9979	0.9584	0.0055	0.0055	0.0059
pRb	0.9991	0.9991	0.9992	0.9992	0.9992	0.9981	0.9979	0.9991	0.9991	0.9991	0.0000	0.0000	0.0000	0.9981	0.9979	0.9992
Ras	0.9946	0.9942	0.9946	0.9946	0.9946	0.9946	0.9946	0.9946	0.9946	0.9942	0.9662	0.0001	0.0001	0.9946	0.9946	0.9946
Bad	0.0000	0.0000	0.0000	0.0000	0.0000	0.0000	0.0000	0.0000	0.0000	0.0000	0.0001	0.0353	0.0459	0.0000	0.0000	0.0000
Bax	0.0000	0.0000	0.0000	0.0000	0.0000	0.0000	0.0000	0.0000	0.0000	0.0000	0.0400	0.9938	0.9937	0.0000	0.0000	0.0000
Bcl-2	0.9941	0.9937	0.9941	0.9598	0.9628	0.0574	0.0574	0.9941	0.9941	0.9937	0.0358	0.0001	0.0000	0.0077	0.0077	0.0567
Bcl-xL	0.9937	0.9933	0.9618	0.9586	0.9937	0.9628	0.9628	0.9618	0.9937	0.9933	0.0528	0.0021	0.0000	0.0583	0.0583	0.0601
Bim	0.0063	0.0063	0.0063	0.0063	0.0062	0.0405	0.0405	0.0063	0.0063	0.0063	0.9750	0.9979	1.0000	0.0405	0.0405	0.0062
Caspase 3	0.0000	0.0000	0.0000	0.0000	0.0000	0.0000	0.0000	0.0000	0.0000	0.0000	0.0000	0.0009	0.0009	0.9186	0.9186	0.9190
Cytochrome C	0.0000	0.0000	0.0000	0.0000	0.0000	0.0000	0.0000	0.0000	0.0000	0.0000	0.0000	0.0455	0.0464	0.9326	0.9326	0.9104
Caspase 8	0.0000	0.0000	0.0000	0.0000	0.0001	0.0000	0.0000	0.0000	0.0000	0.0000	0.0000	0.0001	0.0000	0.0000	0.0000	0.0001
Fas	0.0000	0.0000	0.0000	0.0000	0.0000	0.0000	0.0000	0.0000	0.0000	0.0000	0.0000	0.0001	0.0000	0.0000	0.0000	0.0000
XIAP	0.9982	0.9981	0.9982	0.9945	0.9946	0.9946	0.9946	0.9982	0.9982	0.9981	0.9659	0.9577	0.9444	0.0603	0.0603	0.0602
C/EBPα	0.0008	0.0008	0.0019	0.0321	0.0002	0.0057	0.0057	0.0019	0.0008	0.0008	0.0143	0.0248	0.0024	0.0057	0.0057	0.0002
C/EBPβ	0.0411	0.0405	0.0410	0.0352	0.0390	0.0000	0.0000	0.0410	0.0411	0.0405	0.0434	0.0523	0.0450	0.0000	0.0000	0.0390
Cdx2	0.0413	0.0407	0.0380	0.0347	0.0411	0.0067	0.0067	0.0380	0.0413	0.0407	0.9810	0.9399	0.0018	0.0067	0.0067	0.0411
Gata1/2	0.0019	0.0019	0.0008	0.0008	0.0018	0.0008	0.0008	0.0008	0.0019	0.0019	0.8471	0.9512	0.0000	0.0008	0.0008	0.0018
Gata4/6	0.0019	0.0019	0.0019	0.0019	0.0020	0.0008	0.0008	0.0019	0.0019	0.0019	0.9090	0.0000	0.0000	0.0008	0.0008	0.0020
Hes1	0.0000	0.0001	0.0027	0.0127	0.0000	0.9924	0.9924	0.0027	0.0000	0.0001	0.0000	0.0036	0.0034	0.9924	0.9924	0.0000
Hey2	0.0000	0.0000	0.0018	0.0011	0.0000	0.9423	0.9423	0.0018	0.0000	0.0000	0.0000	0.0024	0.0000	0.9423	0.9423	0.0000
Hoxa10	0.9506	0.9503	0.9558	0.9927	0.9918	0.9621	0.9621	0.9558	0.9506	0.9503	0.0482	0.0000	0.0000	0.9621	0.9621	0.9918
Hoxa3	0.0503	0.0511	0.0465	0.0462	0.0498	0.0491	0.0491	0.0465	0.0503	0.0511	0.9565	0.9452	0.9448	0.0491	0.0491	0.0498
Hoxa5	0.9441	0.9441	0.0479	0.0465	0.9491	0.0474	0.0474	0.0479	0.9441	0.9441	0.9491	0.9406	0.9441	0.0474	0.0474	0.9491
Hoxa9	0.0000	0.0000	0.9502	0.9534	0.0000	0.9489	0.9489	0.9502	0.0000	0.0000	0.0002	0.0083	0.0000	0.9489	0.9489	0.0000
PGC-1	0.9521	0.0016	0.9568	0.9561	0.9519	0.9476	0.9476	0.9568	0.9521	0.0016	0.9513	0.0457	0.0001	0.9476	0.9476	0.9519
PPARγ	0.0001	0.0001	0.0001	0.0060	0.0012	0.0000	0.0000	0.0001	0.0001	0.0001	0.0002	0.0005	0.0001	0.0000	0.0000	0.0012
PRDM14	0.9474	0.9466	0.9514	0.9517	0.9479	0.9487	0.9487	0.9514	0.9474	0.9466	0.0000	0.0000	0.0000	0.9487	0.9487	0.9479
Pu.1	0.0001	0.0001	0.9475	0.9517	0.0001	0.0506	0.0506	0.9475	0.0001	0.0001	0.0000	0.0006	0.0000	0.0506	0.0506	0.0001
Runx1	0.0464	0.0464	0.9793	0.9912	0.0483	0.9824	0.9824	0.9793	0.0464	0.0464	0.0469	0.0555	0.0001	0.9824	0.9824	0.0483
Runx2	0.0063	0.0064	0.0378	0.9845	0.0475	0.0491	0.0491	0.0378	0.0063	0.0064	0.9463	0.0055	0.0034	0.0491	0.0491	0.0475
Sox2	0.9927	0.9926	0.9929	0.9626	0.9600	0.9607	0.9607	0.9929	0.9927	0.9926	0.0488	0.0453	0.0458	0.9607	0.9607	0.9600
Sox4	0.9580	0.9583	0.0482	0.0575	0.9576	0.9607	0.9607	0.0482	0.9580	0.9583	0.9550	0.0000	0.0042	0.9607	0.9607	0.9576
Sox7	0.0529	0.0530	0.0523	0.0545	0.0551	0.0530	0.0530	0.0523	0.0529	0.0530	0.9616	0.0000	0.0000	0.0530	0.0530	0.0551
Sox9	0.0415	0.0474	0.0410	0.0375	0.0375	0.0412	0.0412	0.0410	0.0415	0.0474	0.0349	0.0395	0.0530	0.0412	0.0412	0.0375
IFN-γ	0.9567	0.0026	0.9940	0.9940	0.9576	0.9668	0.9668	0.9940	0.9567	0.0026	0.9622	0.9592	0.0000	0.9668	0.9668	0.9576
IL-1	0.0064	0.0000	0.0064	0.0416	0.0453	0.0030	0.0030	0.0064	0.0064	0.0000	0.0434	0.0487	0.0001	0.0030	0.0030	0.0453
IL-10	0.0000	0.0000	0.0000	0.0000	0.0001	0.0000	0.0000	0.0000	0.0000	0.0000	0.0000	0.0001	0.0000	0.0000	0.0000	0.0001
IL-3	0.0021	0.0021	0.9497	0.9554	0.0023	0.9507	0.9507	0.9497	0.0021	0.0021	0.0046	0.0103	0.0000	0.9507	0.9507	0.0023
IL-6	0.0457	0.0008	0.0459	0.0510	0.0512	0.0511	0.0511	0.0459	0.0457	0.0008	0.0486	0.0489	0.0465	0.0511	0.0511	0.0512
IL-8	0.0523	0.0509	0.0483	0.0480	0.0518	0.0510	0.0510	0.0483	0.0523	0.0509	0.9614	0.9535	0.0037	0.0510	0.0510	0.0518
iκB	0.0000	0.0000	0.0000	0.0001	0.0000	0.0063	0.0063	0.0000	0.0000	0.0000	0.0022	0.0032	0.0037	0.0063	0.0063	0.0000
NF-κB	0.0412	0.0001	0.0418	0.0454	0.0454	0.0487	0.0487	0.0418	0.0412	0.0001	0.0417	0.0473	0.0018	0.0487	0.0487	0.0454
Stat1	0.9460	0.0000	0.9479	0.9317	0.9461	0.0467	0.0467	0.9479	0.9460	0.0000	0.9430	0.9463	0.0000	0.0467	0.0467	0.9461
Stat3	0.9656	0.9606	0.9928	0.9951	0.9688	0.9740	0.9740	0.9928	0.9656	0.9606	0.0024	0.0020	0.0000	0.9740	0.9740	0.9688
Stat5	0.9565	0.9562	0.0453	0.0064	0.9427	0.0433	0.0433	0.0453	0.9565	0.9562	0.0466	0.0001	0.0000	0.0433	0.0433	0.9427
TGF-β	0.0020	0.0071	0.0063	0.0484	0.0067	0.9326	0.9326	0.0063	0.0020	0.0071	0.0064	0.0068	0.0552	0.9326	0.9326	0.0067
TNF-α	0.0022	0.0000	0.0022	0.0130	0.0147	0.0028	0.0028	0.0022	0.0022	0.0000	0.0122	0.0174	0.0000	0.0028	0.0028	0.0147
BMP	0.0069	0.0069	0.0071	0.9815	0.9618	0.0504	0.0504	0.0071	0.0069	0.0069	0.9938	0.0034	0.0000	0.0504	0.0504	0.9618
EGF	0.9515	0.9515	0.9515	0.9515	0.9515	0.9515	0.9515	0.9515	0.9515	0.9515	0.0000	0.0000	0.0000	0.9515	0.9515	0.9515
EGFR	0.9922	0.9913	0.9927	0.9981	0.9981	0.9981	0.9981	0.9927	0.9922	0.9913	0.0524	0.0002	0.0000	0.9981	0.9981	0.9981
FGF2	0.9450	0.9450	0.9461	0.9933	0.9517	0.9929	0.9929	0.9461	0.9450	0.9450	0.9515	0.0000	0.0000	0.9929	0.9929	0.9517
FGF7	0.0000	0.0000	0.0000	0.9521	0.0023	0.9454	0.9454	0.0000	0.0000	0.0000	0.9508	0.0023	0.0000	0.9454	0.9454	0.0023
FGFR2	0.0551	0.0553	0.0545	0.0548	0.0551	0.0552	0.0552	0.0545	0.0551	0.0553	0.9979	0.0000	0.0000	0.0552	0.0552	0.0551
G-CSFR	0.0015	0.0000	0.9512	0.9561	0.0019	0.0227	0.0227	0.9512	0.0015	0.0000	0.0035	0.0076	0.0000	0.0227	0.0227	0.0019
VEGF	0.9974	0.9960	0.9974	0.9972	0.9971	0.9978	0.9978	0.9974	0.9974	0.9960	0.0505	0.0002	0.0002	0.9978	0.9978	0.9971
Akt	0.9979	0.9970	0.9983	0.9996	0.9996	0.9998	0.9998	0.9983	0.9979	0.9970	0.0548	0.0011	0.0000	0.9998	0.9998	0.9996
AP2	0.9520	0.9519	0.9520	0.0017	0.0024	0.0000	0.0000	0.9520	0.9520	0.9519	0.9503	0.9518	0.9510	0.0000	0.0000	0.0024
Bach1	0.9518	0.9524	0.9524	0.9577	0.9524	0.9931	0.9931	0.9524	0.9518	0.9524	0.0000	0.0000	0.0034	0.9931	0.9931	0.9524
c-Jun	0.9941	0.9941	0.9981	0.9975	0.9943	0.9981	0.9981	0.9981	0.9941	0.9941	0.0842	0.0081	0.0000	0.9981	0.9981	0.9943
HIF	0.9934	0.9934	0.9937	0.9937	0.9935	0.9935	0.9935	0.9937	0.9934	0.9934	0.0037	0.0000	0.0000	0.9935	0.9935	0.9935
MAPK	0.9976	0.9954	0.9975	0.9961	0.9983	0.0580	0.0580	0.9975	0.9976	0.9954	0.0539	0.0457	0.0000	0.0580	0.0580	0.9983
p53	0.0020	0.0019	0.0062	0.0061	0.0020	0.0019	0.0019	0.0062	0.0020	0.0019	0.0462	0.9979	0.9932	0.0019	0.0019	0.0020
PKA	0.9937	0.9937	0.9938	0.9942	0.9938	0.9980	0.9980	0.9938	0.9937	0.9937	0.0285	0.0000	0.0002	0.9980	0.9980	0.9938
PTEN	0.0442	0.0505	0.0409	0.0000	0.0000	0.0000	0.0000	0.0409	0.0442	0.0505	0.9503	0.9909	0.9932	0.0000	0.0000	0.0000
HSPG2	0.0000	0.0000	0.0017	0.9566	0.0032	0.9498	0.9498	0.0017	0.0000	0.0000	0.9453	0.0000	0.0040	0.9498	0.9498	0.0032
Integrin	0.9949	0.9950	0.9951	0.0482	0.0478	0.9901	0.9901	0.9951	0.9949	0.9950	0.0307	0.0050	0.0000	0.9901	0.9901	0.0478
NGAL	0.9531	0.0023	0.9936	0.9938	0.9551	0.9933	0.9933	0.9936	0.9531	0.0023	0.9552	0.9565	0.0000	0.9933	0.9933	0.9551
NOG	0.9523	0.9529	0.9523	0.0290	0.0321	0.0351	0.0351	0.9523	0.9523	0.9529	0.0001	0.0012	0.0039	0.0351	0.0351	0.0321
Notch	0.0070	0.0069	0.0452	0.0380	0.0068	0.9346	0.9346	0.0452	0.0070	0.0069	0.0066	0.0496	0.0000	0.9346	0.9346	0.0068
OPN	0.0002	0.0043	0.0516	0.0538	0.0012	0.0551	0.0551	0.0516	0.0002	0.0043	0.0523	0.0062	0.0040	0.0551	0.0551	0.0012
SHH	0.9492	0.9500	0.9493	0.0412	0.0445	0.0409	0.0409	0.9493	0.9492	0.9500	0.0000	0.0000	0.0010	0.0409	0.0409	0.0445
Wnt	0.0334	0.0314	0.0324	0.0358	0.0366	0.0400	0.0400	0.0324	0.0334	0.0314	0.0059	0.0001	0.0041	0.0400	0.0400	0.0366
NR2F2	0.9499	0.9498	0.9498	0.0090	0.0105	0.0105	0.0105	0.9498	0.9499	0.9498	0.0000	0.0000	0.0000	0.0105	0.0105	0.0105
RARs	0.0064	0.0064	0.0064	0.0000	0.0000	0.0000	0.0000	0.0064	0.0064	0.0064	0.9420	0.9435	0.9451	0.0000	0.0000	0.0000

Supplementary Table IV. Recurrence of Attractors Using Different Forms of Equations. Alternative Equation (b). 9 attractors including major functional states S1, S4, and S12 discussed in main text are obtained.

	Attractors Calculated by Alternative Equation (b)									S1	S3	S4	S9	S10	S12	S13	S14	S15
Cdk2	0.9868	0.9915	0.9627	0.9915	0.9868	0.0001	0.0000	0.0000	0.0000	0.8981	0.8988	0.8857	0.8988	0.8981	0.0033	0.0000	0.0013	0.0000
Cdk6	0.3290	0.9734	0.9631	0.9734	0.3290	0.0000	0.0000	0.0000	0.0000	0.8977	0.9556	0.9544	0.9556	0.8977	0.0032	0.0000	0.0006	0.0000
Cyclin D	0.6604	0.6634	0.9923	0.3310	0.3280	0.0000	0.0000	0.0000	0.0000	0.9273	0.9274	0.9598	0.8824	0.8803	0.0019	0.0000	0.0000	0.0000
Cyclin E	0.5816	0.6617	0.9887	0.3303	0.2903	0.0000	0.0000	0.0000	0.0000	0.9071	0.9113	0.9589	0.8588	0.8547	0.0000	0.0000	0.0000	0.0000
E2F	0.5028	0.5031	0.9910	0.0047	0.0045	0.0000	0.0000	0.0000	0.0000	0.8581	0.8584	0.9437	0.0201	0.0203	0.0000	0.0000	0.0000	0.0000
Myc	0.0351	0.0354	0.4981	0.0266	0.0262	0.0000	0.0000	0.0000	0.0000	0.1286	0.1283	0.9416	0.1272	0.1275	0.0037	0.0010	0.0017	0.0000
p21	0.0020	0.0013	0.0000	0.0013	0.0020	0.3267	0.4618	0.4783	0.3155	0.0871	0.0861	0.0016	0.0861	0.0871	0.9050	0.9537	0.9551	0.9364
p27	0.0035	0.0021	0.0014	0.0021	0.0035	0.7225	0.4674	0.7159	0.2480	0.0871	0.0864	0.0463	0.0864	0.0871	0.9275	0.9341	0.9531	0.8916
pRb	0.9820	0.9955	0.9979	0.9727	0.9539	0.0000	0.0000	0.0000	0.0000	0.9677	0.9693	0.9708	0.9669	0.9650	0.0000	0.0000	0.0000	0.0000
Ras	0.4923	0.4945	0.5000	0.4945	0.4921	0.2311	0.0000	0.0000	0.0000	0.9429	0.9429	0.9442	0.9429	0.9429	0.8803	0.0016	0.0021	0.0016
Bad	0.0000	0.0000	0.0000	0.0000	0.0000	0.0019	0.0094	0.0094	0.0283	0.0000	0.0001	0.0001	0.0001	0.0000	0.0022	0.0921	0.0920	0.0968
Bax	0.0000	0.0000	0.0000	0.0000	0.0000	0.0611	0.9724	0.9902	0.9724	0.0001	0.0002	0.0002	0.0002	0.0001	0.1159	0.9479	0.9484	0.9479
Bcl-2	0.6627	0.6630	0.3290	0.6631	0.6628	0.0001	0.0000	0.0007	0.0000	0.9437	0.9412	0.8763	0.9412	0.9437	0.0337	0.0000	0.0022	0.0000
Bcl-xL	0.6153	0.3300	0.3360	0.3299	0.6151	0.0049	0.0000	0.0408	0.0000	0.9362	0.8895	0.8984	0.8895	0.9362	0.0454	0.0000	0.0208	0.0000
Bim	0.0064	0.0043	0.0068	0.0043	0.0064	0.9970	1.0000	1.0000	1.0000	0.0532	0.0526	0.0535	0.0526	0.0532	0.9563	1.0000	0.9903	1.0000
Caspase 3	0.0000	0.0000	0.0000	0.0000	0.0000	0.0000	0.0000	0.0115	0.0006	0.0000	0.0000	0.0000	0.0000	0.0000	0.0000	0.0021	0.0021	0.0024
Cytochrome C	0.0000	0.0000	0.0000	0.0000	0.0000	0.0019	0.0066	0.0060	0.0201	0.0000	0.0000	0.0000	0.0000	0.0000	0.0018	0.0925	0.0922	0.0972
Caspase 8	0.0000	0.0000	0.0001	0.0000	0.0000	0.0000	0.0000	0.0785	0.0000	0.0000	0.0000	0.0001	0.0000	0.0000	0.0002	0.0000	0.0004	0.0000
Fas	0.0000	0.0000	0.0000	0.0000	0.0000	0.0000	0.0000	0.0638	0.0000	0.0000	0.0000	0.0000	0.0000	0.0000	0.0000	0.0000	0.0005	0.0000
XIAP	0.5939	0.5956	0.3936	0.5956	0.5939	0.1969	0.1963	0.2210	0.1963	0.9632	0.9635	0.9465	0.9635	0.9632	0.8583	0.8670	0.8635	0.8670
C/EBPα	0.0008	0.0011	0.0007	0.0011	0.0008	0.0023	0.0017	0.0017	0.0008	0.0294	0.0411	0.0941	0.0411	0.0294	0.0347	0.0320	0.0330	0.0150
C/EBPβ	0.0080	0.0054	0.0026	0.0054	0.0080	0.0171	0.0170	0.0170	0.0170	0.0944	0.0933	0.0864	0.0933	0.0944	0.1183	0.1151	0.1168	0.1151
Cdx2	0.1225	0.0329	0.0413	0.0329	0.1225	0.3865	0.1750	0.1755	0.0003	0.1206	0.1061	0.1042	0.1061	0.1206	0.8942	0.8173	0.8310	0.0079
Gata1/2	0.0020	0.0007	0.0009	0.0007	0.0020	0.3221	0.2428	0.2428	0.0000	0.0356	0.0277	0.0269	0.0277	0.0356	0.8453	0.8452	0.8519	0.0000
Gata4/6	0.0009	0.0005	0.0005	0.0005	0.0009	0.3249	0.0000	0.0000	0.0000	0.0372	0.0358	0.0374	0.0358	0.0372	0.8626	0.0000	0.0000	0.0001
Hes1	0.0001	0.0000	0.0000	0.0000	0.0001	0.0000	0.0000	0.0000	0.0000	0.0035	0.0185	0.0301	0.0185	0.0035	0.0060	0.0345	0.0203	0.0190
Hey2	0.0001	0.0000	0.0000	0.0000	0.0001	0.0000	0.0000	0.0000	0.0000	0.0026	0.0158	0.0099	0.0158	0.0026	0.0024	0.0161	0.0172	0.0000
Hoxa10	0.1135	0.2339	0.4727	0.2339	0.1135	0.0083	0.0000	0.0000	0.0000	0.8579	0.8570	0.9245	0.8570	0.8579	0.1093	0.0000	0.0000	0.0001
Hoxa3	0.0684	0.0184	0.0184	0.0184	0.0684	0.3272	0.3272	0.3279	0.3272	0.1124	0.0993	0.0968	0.0993	0.1124	0.8562	0.8670	0.8623	0.8670
Hoxa5	0.1887	0.0136	0.0172	0.0136	0.1887	0.4886	0.4895	0.4895	0.4907	0.8487	0.0972	0.0980	0.0972	0.8487	0.8400	0.8514	0.8466	0.8670
Hoxa9	0.0002	0.3276	0.3233	0.3276	0.0002	0.0000	0.0000	0.0000	0.0000	0.0028	0.8666	0.8641	0.8666	0.0028	0.0028	0.0164	0.0172	0.0000
PGC-1	0.4567	0.4918	0.4918	0.4918	0.4567	0.4129	0.0001	0.0081	0.0001	0.8666	0.8900	0.8896	0.8900	0.8666	0.8485	0.0016	0.0897	0.0016
PPARγ	0.0000	0.0000	0.0000	0.0000	0.0000	0.0000	0.0000	0.0000	0.0000	0.0011	0.0012	0.0143	0.0012	0.0011	0.0024	0.0020	0.0022	0.0020
PRDM14	0.7923	0.9446	0.9460	0.9446	0.7923	0.0000	0.0000	0.0000	0.0000	0.8743	0.8895	0.8921	0.8895	0.8743	0.0012	0.0000	0.0007	0.0000
Pu.1	0.0087	0.4843	0.4824	0.4843	0.0087	0.0001	0.0002	0.0002	0.0000	0.0022	0.8868	0.8886	0.8868	0.0022	0.0015	0.0026	0.0027	0.0000
Runx1	0.0621	0.3952	0.3804	0.3952	0.0621	0.0207	0.0174	0.0173	0.0000	0.1197	0.9237	0.9307	0.9237	0.1197	0.1274	0.1224	0.1245	0.0011
Runx2	0.0025	0.0076	0.7311	0.0076	0.0025	0.2429	0.0007	0.0006	0.0000	0.0599	0.1225	0.9426	0.1225	0.0599	0.8595	0.0352	0.0213	0.0187
Sox2	0.4348	0.4728	0.2440	0.4728	0.4348	0.0263	0.0047	0.0042	0.0142	0.9250	0.9301	0.8925	0.9301	0.9250	0.1114	0.0896	0.0891	0.0941
Sox4	0.2179	0.0015	0.0075	0.0015	0.2179	0.2494	0.0000	0.0001	0.0000	0.8729	0.0367	0.0480	0.0367	0.8729	0.8761	0.0189	0.0032	0.0190
Sox7	0.0020	0.0012	0.0023	0.0012	0.0020	0.4914	0.0000	0.0011	0.0000	0.1163	0.1118	0.1164	0.1118	0.1163	0.8908	0.0001	0.0001	0.0000
Sox9	0.0067	0.0024	0.0017	0.0024	0.0067	0.0060	0.0029	0.0029	0.0029	0.1016	0.0967	0.0968	0.0967	0.1016	0.1331	0.1154	0.1180	0.1154
IFN-γ	0.2761	0.4917	0.4941	0.4917	0.2761	0.2505	0.0007	0.2806	0.0000	0.8671	0.9361	0.9352	0.9361	0.8671	0.8590	0.0185	0.8665	0.0001
IL-1	0.0026	0.0018	0.0021	0.0018	0.0026	0.0134	0.0000	0.0151	0.0000	0.0526	0.0525	0.0838	0.0525	0.0526	0.1172	0.0021	0.1166	0.0020
IL-10	0.0000	0.0000	0.0001	0.0000	0.0000	0.0000	0.0000	0.0743	0.0000	0.0000	0.0000	0.0001	0.0000	0.0000	0.0002	0.0000	0.0005	0.0000
IL-3	0.0534	0.4843	0.4825	0.4843	0.0534	0.0022	0.0013	0.0013	0.0000	0.0171	0.8874	0.8897	0.8874	0.0171	0.0207	0.0183	0.0193	0.0000
IL-6	0.0086	0.0062	0.0402	0.0062	0.0086	0.0117	0.0059	0.0117	0.0059	0.0973	0.0987	0.1102	0.0987	0.0973	0.1323	0.1152	0.1271	0.1152
IL-8	0.1030	0.0276	0.0271	0.0276	0.1030	0.2493	0.0000	0.2805	0.0000	0.1178	0.1038	0.1012	0.1038	0.1178	0.8574	0.0190	0.8656	0.0190
iκB	0.0000	0.0000	0.0000	0.0000	0.0000	0.0001	0.0000	0.0510	0.0000	0.0006	0.0007	0.0019	0.0007	0.0006	0.0149	0.0190	0.0238	0.0190
NF-κB	0.0053	0.0046	0.0296	0.0046	0.0053	0.0075	0.0002	0.0653	0.0006	0.0971	0.0984	0.1098	0.0984	0.0971	0.1059	0.0023	0.1286	0.0149
Stat1	0.9132	0.9834	0.9831	0.9834	0.9132	0.8869	0.0000	0.9170	0.0000	0.8663	0.8772	0.8592	0.8772	0.8663	0.8455	0.0001	0.8639	0.0000
Stat3	0.1968	0.3243	0.3272	0.3243	0.1968	0.0035	0.0000	0.0010	0.0000	0.8859	0.9304	0.9387	0.9304	0.8859	0.0487	0.0007	0.0409	0.0010
Stat5	0.2376	0.0229	0.0032	0.0229	0.2376	0.0304	0.0000	0.0082	0.0000	0.8818	0.1076	0.0549	0.1076	0.8818	0.1216	0.0001	0.0002	0.0000
TGF-β	0.0004	0.0005	0.0006	0.0005	0.0004	0.0007	0.0050	0.0006	0.0050	0.0445	0.0651	0.1280	0.0651	0.0445	0.0712	0.1245	0.0681	0.1245
TNF-α	0.0000	0.0000	0.0064	0.0000	0.0000	0.0007	0.0000	0.0515	0.0000	0.0105	0.0109	0.0187	0.0109	0.0105	0.0272	0.0000	0.0358	0.0000
BMP	0.0046	0.0063	0.4889	0.0063	0.0046	0.4861	0.0007	0.0006	0.0000	0.0644	0.0677	0.9244	0.0677	0.0644	0.9343	0.0177	0.0189	0.0000
EGF	0.9972	0.9978	0.9978	0.9978	0.9972	0.0000	0.0000	0.0000	0.0000	0.8926	0.8935	0.8939	0.8935	0.8926	0.0001	0.0000	0.0000	0.0000
EGFR	0.4306	0.4958	0.7368	0.4927	0.4279	0.0108	0.0000	0.0000	0.0000	0.9205	0.9295	0.9593	0.9295	0.9205	0.1211	0.0020	0.0022	0.0000
FGF2	0.2113	0.4324	0.9827	0.4324	0.2113	0.4886	0.0000	0.0000	0.0000	0.8633	0.8629	0.9376	0.8629	0.8633	0.8660	0.0001	0.0000	0.0001
FGF7	0.0000	0.0000	0.4920	0.0000	0.0000	0.4890	0.0000	0.0009	0.0000	0.0015	0.0015	0.8772	0.0015	0.0015	0.8661	0.0001	0.0156	0.0001
FGFR2	0.0012	0.0011	0.0023	0.0011	0.0012	0.7110	0.0000	0.0000	0.0000	0.1207	0.1153	0.1196	0.1153	0.1207	0.9509	0.0004	0.0001	0.0001
G-CSFR	0.0001	0.3276	0.3318	0.3276	0.0001	0.0001	0.0000	0.0408	0.0000	0.0093	0.8747	0.8756	0.8747	0.0093	0.0121	0.0003	0.0212	0.0001
VEGF	0.6625	0.6988	0.4185	0.6988	0.6625	0.0051	0.0000	0.0000	0.0000	0.9607	0.9634	0.9510	0.9634	0.9607	0.1319	0.0025	0.0015	0.0025
Akt	0.5411	0.6176	0.6223	0.6175	0.5410	0.0113	0.0000	0.0002	0.0000	0.9632	0.9676	0.9765	0.9676	0.9632	0.1306	0.0018	0.0043	0.0000
AP2	0.3261	0.3278	0.0006	0.3278	0.3261	0.3279	0.3280	0.3280	0.3280	0.8676	0.8684	0.0065	0.8684	0.8676	0.8574	0.8673	0.8632	0.8673
Bach1	0.4986	0.4989	0.4989	0.4989	0.4986	0.0000	0.0000	0.0000	0.0000	0.8926	0.8936	0.8942	0.8936	0.8926	0.0037	0.0190	0.0032	0.0190
c-Jun	0.5175	0.7360	0.7380	0.7359	0.5174	0.0014	0.0007	0.0166	0.0000	0.9434	0.9608	0.9553	0.9608	0.9434	0.0574	0.0180	0.0194	0.0000
HIF	0.8899	0.9681	0.9689	0.9681	0.8899	0.0003	0.0000	0.0000	0.0000	0.9402	0.9432	0.9446	0.9432	0.9402	0.0225	0.0000	0.0001	0.0000
MAPK	0.5311	0.6145	0.3723	0.6145	0.5311	0.0167	0.0000	0.0014	0.0000	0.9608	0.9642	0.9424	0.9642	0.9609	0.1329	0.0000	0.0993	0.0000
p53	0.0023	0.0018	0.0027	0.0018	0.0023	0.0537	0.3289	0.4812	0.3289	0.0393	0.0549	0.0551	0.0549	0.0393	0.1225	0.9363	0.9536	0.9363
PKA	0.6544	0.6585	0.6623	0.6584	0.6542	0.0004	0.0000	0.0000	0.0000	0.9431	0.9432	0.9462	0.9432	0.9431	0.0411	0.0021	0.0003	0.0021
PTEN	0.0655	0.0175	0.0000	0.0175	0.0655	0.3393	0.6310	0.5643	0.6310	0.1089	0.0957	0.0002	0.0957	0.1089	0.8523	0.9364	0.9178	0.9364
HSPG2	0.0000	0.0001	0.4975	0.0001	0.0000	0.4387	0.0000	0.0000	0.0000	0.0030	0.0207	0.8936	0.0207	0.0030	0.8640	0.0194	0.0032	0.0190
Integrin	0.7287	0.7297	0.0117	0.7389	0.7379	0.0006	0.0000	0.0160	0.0000	0.9413	0.9417	0.1027	0.9422	0.9418	0.0445	0.0000	0.0101	0.0000
NGAL	0.3400	0.6507	0.6496	0.6507	0.3400	0.2972	0.0009	0.3278	0.0000	0.8673	0.9415	0.9420	0.9415	0.8673	0.8641	0.0181	0.8671	0.0000
NOG	0.2977	0.3275	0.0100	0.3275	0.2977	0.0000	0.0000	0.0000	0.0000	0.8534	0.8572	0.0296	0.8572	0.8534	0.0026	0.0151	0.0162	0.0152
Notch	0.0065	0.0040	0.0042	0.0040	0.0065	0.0042	0.0031	0.0036	0.0000	0.0640	0.1171	0.1001	0.1171	0.0640	0.0623	0.1178	0.1205	0.0000
OPN	0.0007	0.0053	0.0108	0.0053	0.0007	0.0081	0.0009	0.0009	0.0000	0.0026	0.1103	0.1172	0.1103	0.0026	0.1216	0.0367	0.0219	0.0190
SHH	0.2543	0.4821	0.0074	0.4821	0.2543	0.0001	0.0000	0.0000	0.0000	0.8697	0.8748	0.0984	0.8748	0.8697	0.0010	0.0018	0.0007	0.0029
Wnt	0.0141	0.0061	0.0395	0.0061	0.0141	0.0069	0.0000	0.0000	0.0000	0.1037	0.0964	0.1075	0.0964	0.1037	0.0608	0.0029	0.0015	0.0186
NR2F2	0.4465	0.4913	0.0151	0.4913	0.4465	0.0001	0.0000	0.0000	0.0000	0.8682	0.8702	0.0215	0.8702	0.8682	0.0022	0.0000	0.0000	0.0001
RARs	0.0039	0.0027	0.0000	0.0027	0.0039	0.4721	0.4732	0.4732	0.4732	0.0528	0.0524	0.0000	0.0524	0.0528	0.8433	0.8671	0.8571	0.8671

Supplementary Table V. Attractors: Boolean Dynamics *vs.* Differential Equations.

Inside each slot is comparison of corresponding attractors obtained separately from Boolean dynamics (left) and differential equations (right). The comparison result demonstrates consistency between the two distinct methods as a verification on the robustness of our calculated attractors.

	S1		S2		S3		S4		S5		S6		S7		S8		S9	
Cdk2	1	0.898	1	0.896	1	0.899	1	0.886	1	0.897	1	0.887	0	0.016	0	0.016	1	0.899
Cdk6	1	0.898	1	0.896	1	0.956	1	0.954	1	0.897	1	0.887	1	0.861	1	0.861	1	0.956
Cyclin D	1	0.927	1	0.933	1	0.927	1	0.96	1	0.962	1	0.962	1	0.937	1	0.896	1	0.882
Cyclin E	1	0.907	1	0.913	1	0.911	1	0.959	1	0.96	1	0.96	1	0.935	1	0.891	1	0.859
E2F	1	0.858	1	0.858	1	0.858	1	0.944	1	0.945	1	0.944	1	0.866	0	0.014	0	0.02
Myc	0	0.129	0	0.129	0	0.128	1	0.942	1	0.96	1	0.941	0	0.116	0	0.114	0	0.127
p21	0	0.087	0	0.087	0	0.086	0	0.002	0	2E-04	0	0.001	0	0.048	0	0.048	0	0.086
p27	0	0.087	0	0.002	0	0.086	0	0.046	0	0.045	0	0.001	0	0.048	0	0.048	0	0.086
pRb	1	0.968	1	0.968	1	0.969	1	0.971	1	0.97	1	0.969	1	0.958	1	0.954	1	0.967
Ras	1	0.943	1	0.942	1	0.943	1	0.944	1	0.945	1	0.943	1	0.946	1	0.946	1	0.943
Bad	0	3E-05	0	3E-05	0	9E-05	0	9E-05	0	4E-05	0	3E-05	0	5E-05	0	5E-05	0	9E-05
Bax	0	8E-05	0	8E-05	0	2E-04	0	2E-04	0	9E-05	0	1E-04	0	5E-04	0	5E-04	0	2E-04
Bcl-2	1	0.944	1	0.941	1	0.941	1	0.876	1	0.892	1	0.87	0	0.114	0	0.114	1	0.941
Bcl-xL	1	0.936	1	0.936	1	0.89	1	0.898	1	0.938	1	0.937	1	0.898	1	0.898	1	0.89
Bim	0	0.053	0	0.054	0	0.053	0	0.054	0	0.052	0	0.055	0	0.096	0	0.096	0	0.053
Caspase 3	0	1E-06	0	2E-14	0	1E-06	0	7E-06	0	8E-06	0	4E-14	0	3E-06	0	3E-06	0	1E-06
Cytochrome C	0	4E-13	0	3E-13	0	3E-12	0	5E-12	0	4E-13	0	6E-13	0	2E-10	0	2E-10	0	3E-12
Caspase 8	0	1E-05	0	9E-24	0	1E-05	0	6E-05	0	7E-05	0	2E-23	0	3E-05	0	3E-05	0	1E-05
Fas	0	1E-06	0	9E-25	0	1E-06	0	7E-06	0	8E-06	0	2E-24	0	3E-06	0	3E-06	0	1E-06
XIAP	1	0.963	1	0.963	1	0.963	1	0.947	1	0.948	1	0.945	1	0.946	1	0.946	1	0.964
C/EBPα	0	0.029	0	0.029	0	0.041	0	0.094	0	0.002	0	0.002	0	0.057	0	0.057	0	0.041
C/EBPβ	0	0.094	0	0.094	0	0.093	0	0.086	0	0.087	0	0.086	0	9E-04	0	9E-04	0	0.093
Cdx2	0	0.121	0	0.121	0	0.106	0	0.104	0	0.118	0	0.12	0	0.061	0	0.061	0	0.106
Gata1/2	0	0.036	0	0.036	0	0.028	0	0.027	0	0.034	0	0.035	0	0.028	0	0.028	0	0.028
Gata4/6	0	0.037	0	0.037	0	0.036	0	0.037	0	0.039	0	0.039	0	0.03	0	0.03	0	0.036
Hes1	0	0.003	0	0.005	0	0.018	0	0.03	0	0.005	0	0.024	1	0.931	1	0.931	0	0.018
Hey2	0	0.003	0	0.003	0	0.016	0	0.01	0	0.002	0	0.002	1	0.863	1	0.863	0	0.016
Hoxa10	1	0.858	1	0.857	1	0.857	1	0.924	1	0.916	1	0.913	1	0.859	1	0.859	1	0.857
Hoxa3	0	0.112	0	0.113	0	0.099	0	0.097	0	0.11	0	0.111	0	0.109	0	0.109	0	0.099
Hoxa5	1	0.849	1	0.848	0	0.097	0	0.098	1	0.867	1	0.865	0	0.094	0	0.094	0	0.097
Hoxa9	0	0.003	0	0.003	1	0.867	1	0.864	0	0.002	0	0.002	1	0.851	1	0.851	1	0.867
PGC-1	1	0.867	0	0.008	1	0.89	1	0.89	1	0.866	0	0.006	1	0.876	1	0.876	1	0.89
PPARγ	0	0.001	0	0.001	0	0.001	0	0.014	0	0.007	0	0.006	0	2E-04	0	2E-04	0	0.001
PRDM14	1	0.874	1	0.873	1	0.89	1	0.892	1	0.878	1	0.876	1	0.879	1	0.879	1	0.89
Pu.1	0	0.002	0	0.002	1	0.887	1	0.889	0	0.003	0	0.003	0	0.115	0	0.115	1	0.887
Runx1	0	0.12	0	0.12	1	0.924	1	0.931	0	0.128	0	0.13	1	0.934	1	0.934	1	0.924
Runx2	0	0.06	0	0.06	0	0.122	1	0.943	0	0.127	0	0.13	0	0.131	0	0.131	0	0.122
Sox2	1	0.925	1	0.925	1	0.93	1	0.892	1	0.878	1	0.876	1	0.878	1	0.878	1	0.93
Sox4	1	0.873	1	0.872	0	0.037	0	0.048	1	0.865	1	0.862	1	0.878	1	0.878	0	0.037
Sox7	0	0.116	0	0.117	0	0.112	0	0.116	0	0.121	0	0.122	0	0.115	0	0.115	0	0.112
Sox9	0	0.102	0	0.102	0	0.097	0	0.097	0	0.099	0	0.099	0	0.095	0	0.095	0	0.097
IFN-γ	1	0.867	0	0.017	1	0.936	1	0.935	1	0.866	0	0.024	1	0.891	1	0.891	1	0.936
IL-1	0	0.053	0	5E-04	0	0.052	0	0.084	0	0.084	0	8E-04	0	0.004	0	0.004	0	0.052
IL-10	0	1E-05	0	9E-24	0	1E-05	0	7E-05	0	7E-05	0	2E-23	0	3E-05	0	3E-05	0	1E-05
IL-3	0	0.017	0	0.017	1	0.887	1	0.89	0	0.02	0	0.022	1	0.891	1	0.891	1	0.887
IL-6	0	0.097	0	0.003	0	0.099	0	0.11	0	0.112	0	0.003	0	0.115	0	0.115	0	0.099
IL-8	0	0.118	0	0.113	0	0.104	0	0.101	0	0.115	0	0.111	0	0.114	0	0.114	0	0.104
iκB	0	6E-04	0	2E-04	0	7E-04	0	0.002	0	1E-03	0	0.001	0	0.05	0	0.05	0	7E-04
NF-κB	0	0.097	0	9E-04	0	0.098	0	0.11	0	0.111	0	8E-04	0	0.113	0	0.113	0	0.098
Stat1	1	0.866	0	5E-05	1	0.877	1	0.859	1	0.864	0	1E-04	0	0.106	0	0.106	1	0.877
Stat3	1	0.886	1	0.884	1	0.93	1	0.939	1	0.895	1	0.89	1	0.899	1	0.899	1	0.93
Stat5	1	0.882	1	0.88	0	0.108	0	0.055	1	0.859	1	0.852	0	0.102	0	0.102	0	0.108
TGF-β	0	0.044	0	0.065	0	0.065	0	0.128	0	0.068	0	0.13	1	0.898	1	0.898	0	0.065
TNF-α	0	0.011	0	1E-08	0	0.011	0	0.019	0	0.019	0	1E-08	0	0.014	0	0.014	0	0.011
BMP	0	0.064	0	0.064	0	0.068	1	0.924	1	0.895	1	0.878	0	0.114	0	0.114	0	0.068
EGF	1	0.893	1	0.893	1	0.894	1	0.894	1	0.893	1	0.893	1	0.893	1	0.893	1	0.894
EGFR	1	0.921	1	0.92	1	0.929	1	0.959	1	0.957	1	0.956	1	0.957	1	0.957	1	0.929
FGF2	1	0.863	1	0.863	1	0.863	1	0.938	1	0.885	1	0.884	1	0.929	1	0.929	1	0.863
FGF7	0	0.001	0	1E-06	0	0.002	1	0.877	0	0.006	0	7E-04	1	0.872	1	0.872	0	0.002
FGFR2	0	0.121	0	0.121	0	0.115	0	0.12	0	0.121	0	0.121	0	0.123	0	0.123	0	0.115
G-CSFR	0	0.009	0	3E-04	1	0.875	1	0.876	0	0.014	0	3E-07	0	0.03	0	0.03	1	0.875
VEGF	1	0.961	1	0.954	1	0.963	1	0.951	1	0.948	1	0.93	1	0.959	1	0.959	1	0.963
Akt	1	0.963	1	0.963	1	0.968	1	0.977	1	0.976	1	0.976	1	0.98	1	0.98	1	0.968
AP2	1	0.868	1	0.868	1	0.868	0	0.007	0	0.007	0	0.006	0	2E-04	0	2E-04	1	0.868
Bach1	1	0.893	1	0.893	1	0.894	1	0.894	1	0.893	1	0.893	1	0.94	1	0.94	1	0.894
c-Jun	1	0.943	1	0.943	1	0.961	1	0.955	1	0.948	1	0.948	1	0.962	1	0.962	1	0.961
HIF	1	0.94	1	0.94	1	0.943	1	0.945	1	0.942	1	0.942	1	0.943	1	0.943	1	0.943
MAPK	1	0.961	1	0.954	1	0.964	1	0.942	1	0.957	1	0.925	0	0.118	0	0.118	1	0.964
p53	0	0.039	0	0.037	0	0.055	0	0.055	0	0.04	0	0.039	0	0.037	0	0.037	0	0.055
PKA	1	0.943	1	0.943	1	0.943	1	0.946	1	0.947	1	0.947	1	0.962	1	0.962	1	0.943
PTEN	0	0.109	0	0.11	0	0.096	0	2E-04	0	8E-05	0	8E-05	0	6E-05	0	6E-05	0	0.096
HSPG2	0	0.003	0	0.005	0	0.021	1	0.894	0	0.023	0	0.042	1	0.879	1	0.879	0	0.021
Integrin	1	0.941	1	0.941	1	0.942	0	0.103	0	0.098	0	0.103	1	0.927	1	0.927	1	0.942
NGAL	1	0.867	0	0.017	1	0.941	1	0.942	1	0.867	0	0.022	1	0.938	1	0.938	1	0.941
NOG	1	0.853	1	0.853	1	0.857	0	0.03	0	0.036	0	0.035	0	0.036	0	0.036	1	0.857
Notch	0	0.064	0	0.064	0	0.117	0	0.1	0	0.06	0	0.062	1	0.858	1	0.858	0	0.117
OPN	0	0.003	0	0.022	0	0.11	0	0.117	0	0.006	0	0.062	0	0.122	0	0.122	0	0.11
SHH	1	0.87	1	0.869	1	0.875	0	0.098	0	0.107	0	0.112	0	0.103	0	0.103	1	0.875
Wnt	0	0.104	0	0.097	0	0.096	0	0.108	0	0.119	0	0.111	0	0.122	0	0.122	0	0.096
NR2F2	1	0.868	1	0.868	1	0.87	0	0.021	0	0.028	0	0.027	0	0.028	0	0.028	1	0.87
RARs	0	0.053	0	0.053	0	0.052	0	3E-06	0	6E-07	0	6E-07	0	2E-10	0	2E-10	0	0.052

(*Continued on next page*)

Supplementary Table V. Attractors: Boolean Dynamics *vs.* Differential Equations.

	S10		S11		S12		S13		S14		S15		S16		S17		S18	
Cdk2	1	0.898	1	0.896	0	0.003	0	5E-16	0	0.001	0	5E-16	0	0.016	0	0.016	1	0.887
Cdk6	1	0.898	1	0.896	0	0.003	0	3E-06	0	6E-04	0	9E-14	1	0.861	1	0.861	1	0.887
Cyclin D	1	0.88	1	0.886	0	0.002	0	5E-09	0	8E-09	0	3E-16	1	0.937	1	0.896	1	0.962
Cyclin E	1	0.855	1	0.86	0	5E-08	0	3E-06	0	1E-08	0	3E-06	1	0.935	1	0.891	1	0.96
E2F	0	0.02	0	0.02	0	6E-08	0	1E-09	0	5E-09	0	7E-17	1	0.866	0	0.014	1	0.944
Myc	0	0.128	0	0.127	0	0.004	0	0.001	0	0.002	0	4E-06	0	0.116	0	0.114	1	0.941
p21	0	0.087	0	0.087	1	0.905	1	0.954	1	0.955	1	0.936	0	0.048	0	0.048	0	0.001
p27	0	0.087	0	0.002	1	0.927	1	0.934	1	0.953	1	0.892	0	0.048	0	0.048	0	0.001
pRb	1	0.965	1	0.965	0	8E-07	0	4E-16	0	3E-08	0	2E-16	1	0.958	1	0.954	1	0.969
Ras	1	0.943	1	0.942	1	0.88	0	0.002	0	0.002	0	0.002	1	0.946	1	0.946	1	0.943
Bad	0	3E-05	0	3E-05	0	0.002	0	0.092	0	0.092	0	0.097	0	5E-05	0	5E-05	0	3E-05
Bax	0	8E-05	0	8E-05	0	0.116	1	0.948	1	0.948	1	0.948	0	5E-04	0	5E-04	0	1E-04
Bcl-2	1	0.944	1	0.942	0	0.034	0	3E-08	0	0.002	0	4E-06	0	0.071	0	0.071	0	0.147
Bcl-xL	1	0.936	1	0.936	0	0.045	0	2E-07	0	0.021	0	3E-05	0	0.147	0	0.147	0	0.155
Bim	0	0.053	0	0.054	1	0.956	1	1	1	0.99	1	1	0	0.096	0	0.096	0	0.055
Caspase 3	0	1E-06	0	2E-14	0	3E-05	0	0.002	0	0.002	0	0.002	1	0.799	1	0.799	1	0.796
Cytochrome C	0	4E-13	0	3E-13	0	0.002	0	0.093	0	0.092	0	0.097	1	0.806	1	0.806	1	0.781
Caspase 8	0	1E-05	0	9E-24	0	2E-04	0	1E-19	0	4E-04	0	4E-13	0	3E-05	0	3E-05	0	2E-23
Fas	0	1E-06	0	9E-25	0	3E-05	0	1E-19	0	5E-04	0	4E-13	0	3E-06	0	3E-06	0	2E-24
XIAP	1	0.963	1	0.963	1	0.858	1	0.867	1	0.864	1	0.867	0	0.155	0	0.155	0	0.157
C/EBPα	0	0.029	0	0.029	0	0.035	0	0.032	0	0.033	0	0.015	0	0.057	0	0.057	0	0.002
C/EBPβ	0	0.094	0	0.094	0	0.118	0	0.115	0	0.117	0	0.115	0	9E-04	0	9E-04	0	0.086
Cdx2	0	0.121	0	0.121	1	0.894	1	0.817	1	0.831	0.5	0.008	0	0.061	0	0.061	0	0.12
Gata1/2	0	0.036	0	0.036	1	0.845	1	0.845	1	0.852	0.5	5E-06	0	0.028	0	0.028	0	0.035
Gata4/6	0	0.037	0	0.037	1	0.863	0	3E-07	0	4E-08	0	6E-05	0	0.03	0	0.03	0	0.039
Hes1	0	0.003	0	0.005	0	0.006	0	0.034	0	0.02	0	0.019	1	0.931	1	0.931	0	0.024
Hey2	0	0.003	0	0.003	0	0.002	0	0.016	0	0.017	0	1E-15	1	0.863	1	0.863	0	0.002
Hoxa10	1	0.858	1	0.857	0	0.109	0	2E-05	0	2E-05	0	6E-05	1	0.859	1	0.859	1	0.913
Hoxa3	0	0.112	0	0.113	1	0.856	1	0.867	1	0.862	1	0.867	0	0.109	0	0.109	0	0.111
Hoxa5	1	0.849	1	0.848	1	0.84	1	0.851	1	0.847	1	0.867	0	0.094	0	0.094	1	0.865
Hoxa9	0	0.003	0	0.003	0	0.003	0	0.016	0	0.017	0	3E-05	1	0.851	1	0.851	0	0.002
PGC-1	1	0.867	0	0.008	1	0.848	0	0.002	0	0.09	0	0.002	1	0.876	1	0.876	0	0.006
PPARγ	0	0.001	0	0.001	0	0.002	0	0.002	0	0.002	0	0.002	0	2E-04	0	2E-04	0	0.006
PRDM14	1	0.874	1	0.873	0	0.001	0	4E-09	0	7E-04	0	9E-09	1	0.879	1	0.879	1	0.876
Pu.1	0	0.002	0	0.002	0	0.002	0	0.003	0	0.003	0	3E-05	0	0.115	0	0.115	0	0.003
Runx1	0	0.12	0	0.12	0	0.127	0	0.122	0	0.124	0	0.001	1	0.934	1	0.934	0	0.13
Runx2	0	0.06	0	0.06	1	0.859	0	0.035	0	0.021	0	0.019	0	0.131	0	0.131	0	0.13
Sox2	1	0.925	1	0.925	0	0.111	0	0.09	0	0.089	0	0.094	1	0.878	1	0.878	1	0.876
Sox4	1	0.873	1	0.872	1	0.876	0	0.019	0	0.003	0	0.019	1	0.878	1	0.878	1	0.862
Sox7	0	0.116	0	0.117	1	0.891	0	1E-04	0	1E-04	0	5E-23	0	0.115	0	0.115	0	0.122
Sox9	0	0.102	0	0.102	0	0.133	0	0.115	0	0.118	0	0.115	0	0.095	0	0.095	0	0.099
IFN-γ	1	0.867	0	0.017	1	0.859	0	0.018	1	0.866	0.5	1E-04	1	0.891	1	0.891	0	0.024
IL-1	0	0.053	0	5E-04	0	0.117	0	0.002	0	0.117	0	0.002	0	0.004	0	0.004	0	8E-04
IL-10	0	1E-05	0	9E-24	0	2E-04	0	1E-19	0	5E-04	0	4E-13	0	3E-05	0	3E-05	0	2E-23
IL-3	0	0.017	0	0.017	0	0.021	0	0.018	0	0.019	0	3E-05	1	0.891	1	0.891	0	0.022
IL-6	0	0.097	0	0.003	0	0.132	0	0.115	0	0.127	0	0.115	0	0.115	0	0.115	0	0.003
IL-8	0	0.118	0	0.113	1	0.857	0	0.019	1	0.866	0.5	0.019	0	0.114	0	0.114	0	0.111
iκB	0	6E-04	0	2E-04	0	0.015	0	0.019	0	0.024	0	0.019	0	0.05	0	0.05	0	0.001
NF-κB	0	0.097	0	9E-04	0	0.106	0	0.002	0	0.129	0	0.015	0	0.113	0	0.113	0	8E-04
Stat1	1	0.866	0	5E-05	1	0.845	0	6E-05	1	0.864	0.5	1E-11	0	0.106	0	0.106	0	1E-04
Stat3	1	0.886	1	0.884	0	0.049	0	7E-04	0	0.041	0	1E-03	1	0.899	1	0.899	1	0.89
Stat5	1	0.882	1	0.88	0	0.122	0	6E-05	0	2E-04	0	2E-07	0	0.102	0	0.102	1	0.852
TGF-β	0	0.044	0	0.065	0	0.071	0	0.125	0	0.068	0	0.125	1	0.898	1	0.898	0	0.13
TNF-α	0	0.011	0	1E-08	0	0.027	0	2E-07	0	0.036	0	3E-05	0	0.014	0	0.014	0	1E-08
BMP	0	0.064	0	0.064	1	0.934	0	0.018	0	0.019	0	1E-08	0	0.114	0	0.114	1	0.878
EGF	1	0.893	1	0.893	0	1E-04	0	2E-24	0	4E-12	0	1E-26	1	0.893	1	0.893	1	0.893
EGFR	1	0.921	1	0.92	0	0.121	0	0.002	0	0.002	0	7E-06	1	0.957	1	0.957	1	0.956
FGF2	1	0.863	1	0.863	1	0.866	0	7E-05	0	3E-07	0	7E-05	1	0.929	1	0.929	1	0.884
FGF7	0	0.001	0	1E-06	1	0.866	0	7E-05	0	0.016	0	7E-05	1	0.872	1	0.872	0	7E-04
FGFR2	0	0.121	0	0.121	1	0.951	0	4E-04	0	1E-04	0	7E-05	0	0.123	0	0.123	0	0.121
G-CSFR	0	0.009	0	3E-04	0	0.012	0	3E-04	0	0.021	0	7E-05	0	0.03	0	0.03	0	3E-07
VEGF	1	0.961	1	0.954	0	0.132	0	0.003	0	0.001	0	0.003	1	0.959	1	0.959	1	0.93
Akt	1	0.963	1	0.963	0	0.131	0	0.002	0	0.004	0	4E-06	1	0.98	1	0.98	1	0.976
AP2	1	0.868	1	0.868	1	0.857	1	0.867	1	0.863	1	0.867	0	2E-04	0	2E-04	0	0.006
Bach1	1	0.893	1	0.893	0	0.004	0	0.019	0	0.003	0	0.019	1	0.94	1	0.94	1	0.893
c-Jun	1	0.943	1	0.943	0	0.057	0	0.018	0	0.019	0	1E-08	1	0.962	1	0.962	1	0.948
HIF	1	0.94	1	0.94	0	0.022	0	6E-09	0	7E-05	0	1E-09	1	0.943	1	0.943	1	0.942
MAPK	1	0.961	1	0.954	0	0.133	0	7E-06	0	0.099	0	4E-06	0	0.118	0	0.118	1	0.925
p53	0	0.039	0	0.037	0	0.122	1	0.936	1	0.954	1	0.936	0	0.037	0	0.037	0	0.039
PKA	1	0.943	1	0.943	0	0.041	0	0.002	0	3E-04	0	0.002	1	0.962	1	0.962	1	0.947
PTEN	0	0.109	0	0.11	1	0.852	1	0.936	1	0.918	1	0.936	0	6E-05	0	6E-05	0	8E-05
HSPG2	0	0.003	0	0.005	1	0.864	0	0.019	0	0.003	0	0.019	1	0.879	1	0.879	0	0.042
Integrin	1	0.942	1	0.941	0	0.045	0	2E-07	0	0.01	0	2E-07	1	0.927	1	0.927	0	0.103
NGAL	1	0.867	0	0.017	1	0.864	0	0.018	1	0.867	0.5	1E-08	1	0.938	1	0.938	0	0.022
NOG	1	0.853	1	0.853	0	0.003	0	0.015	0	0.016	0	0.015	0	0.036	0	0.036	0	0.035
Notch	0	0.064	0	0.064	0	0.062	0	0.118	0	0.121	0	5E-06	1	0.858	1	0.858	0	0.062
OPN	0	0.003	0	0.022	0	0.122	0	0.037	0	0.022	0	0.019	0	0.122	0	0.122	0	0.062
SHH	1	0.87	1	0.869	0	0.001	0	0.002	0	7E-04	0	0.003	0	0.103	0	0.103	0	0.112
Wnt	0	0.104	0	0.097	0	0.061	0	0.003	0	0.002	0	0.019	0	0.122	0	0.122	0	0.111
NR2F2	1	0.868	1	0.868	0	0.002	0	3E-07	0	4E-08	0	6E-05	0	0.028	0	0.028	0	0.027
RARs	0	0.053	0	0.053	1	0.843	1	0.867	1	0.857	1	0.867	0	2E-10	0	2E-10	0	6E-07

Supplementary Table VI. Saddle Points from Differential Equations.

The 32 saddle points are first obtained while calculating with 100,000 random initial positions using *Newtonian iteration method.* We further run the program with 1,000,000 initial positions, acquiring an identical result.

	Saddle 1	Saddle 2	Saddle 3	Saddle 4	Saddle 5	Saddle 6	Saddle 7	Saddle 8	Saddle 9	Saddle 10	Saddle 11	Saddle 12	Saddle 13	Saddle 14	Saddle 15	Saddle 16
Connected Attractors & Times (Tot. 500 perturbations)	S1 & S2	S1 & S3	S1 & S5	S1 & S10	S2 & S6	S2 & S11	S2 & S18	S3 & S4	S3 & S9	S4 & S5	S4 & S7	S4 & S12	S4 & S14	S4 & S16	S5 & S6	S5 & S15
	228;272	229;271	267;233	273;227	242;258	257;243	261;239	239;261	234;266	254;246	246;254	262;238	252;248	245;255	245;255	230;270
Cdk2	0.8963	0.8970	0.8972	0.8981	0.8887	0.8960	0.8887	0.8902	0.8988	0.8953	0.5898	0.1645	0.1204	0.5898	0.8895	0.0069
Cdk6	0.8960	0.8971	0.8967	0.8977	0.8882	0.8957	0.8882	0.9550	0.9556	0.8945	0.8122	0.1643	0.0348	0.8122	0.8890	0.0008
Cyclin D	0.9328	0.9235	0.9536	0.8883	0.9552	0.8941	0.9552	0.9433	0.8899	0.9607	0.9423	0.0444	0.0002	0.9423	0.9618	0.0000
Cyclin E	0.9121	0.9070	0.9512	0.8639	0.9526	0.8692	0.9526	0.9403	0.8680	0.9595	0.9395	0.0058	0.0000	0.9395	0.9599	0.0000
E2F	0.8581	0.8580	0.9292	0.4065	0.9287	0.4065	0.9287	0.9061	0.4065	0.9448	0.8952	0.0017	0.0000	0.8952	0.9441	0.0000
Myc	0.1286	0.1286	0.7989	0.1277	0.7947	0.1276	0.7947	0.6069	0.1274	0.9535	0.5192	0.0782	0.0315	0.5192	0.9461	0.0005
p21	0.0872	0.0872	0.0111	0.0871	0.0114	0.0872	0.0114	0.0287	0.0861	0.0006	0.0334	0.5571	0.8586	0.0334	0.0009	0.9327
p27	0.0412	0.0873	0.0562	0.0871	0.0012	0.0016	0.0012	0.0668	0.0864	0.0455	0.0483	0.6875	0.8616	0.0483	0.0220	0.7946
pRb	0.9678	0.9675	0.9694	0.9654	0.9691	0.9656	0.9691	0.9701	0.9673	0.9697	0.9601	0.0824	0.0175	0.9601	0.9695	0.0000
Ras	0.9424	0.9400	0.9443	0.9429	0.9430	0.9424	0.9430	0.9433	0.9429	0.9440	0.9436	0.8891	0.0420	0.9436	0.9435	0.0019
Bad	0.0000	0.0001	0.0000	0.0000	0.0000	0.0000	0.0000	0.0001	0.0001	0.0001	0.0001	0.0237	0.1021	0.0001	0.0000	0.0939
Bax	0.0001	0.0001	0.0001	0.0001	0.0001	0.0001	0.0001	0.0002	0.0002	0.0001	0.0006	0.0756	0.7635	0.0006	0.0001	0.9436
Bcl-2	0.9419	0.9420	0.8930	0.9437	0.8752	0.9415	0.1474	0.8844	0.9412	0.8861	0.4944	0.5544	0.0907	0.1357	0.8753	0.0281
Bcl-xL	0.9359	0.8916	0.9378	0.9362	0.9371	0.9359	0.1555	0.8943	0.8895	0.9157	0.8945	0.2539	0.4171	0.1536	0.9374	0.2410
Bim	0.0537	0.0533	0.0524	0.0532	0.0546	0.0538	0.0546	0.0532	0.0526	0.0529	0.0849	0.6250	0.7451	0.0849	0.0544	0.9918
Caspase 3	0.0000	0.0000	0.0000	0.0000	0.0000	0.0000	0.7951	0.0000	0.0000	0.0000	0.0000	0.0132	0.0629	0.7843	0.0000	0.0174
Cytochrome C	0.0000	0.0000	0.0000	0.0000	0.0000	0.0000	0.7798	0.0000	0.0000	0.0000	0.0000	0.0010	0.1013	0.7803	0.0000	0.0966
Caspase 8	0.0000	0.0000	0.0001	0.0000	0.0000	0.0000	0.0000	0.0001	0.0000	0.0001	0.0000	0.0619	0.2152	0.0000	0.0000	0.0906
Fas	0.0000	0.0000	0.0000	0.0000	0.0000	0.0000	0.0000	0.0000	0.0000	0.0000	0.0000	0.0083	0.2483	0.0000	0.0000	0.1082
XIAP	0.9629	0.9631	0.9477	0.9632	0.9455	0.9629	0.1569	0.9476	0.9635	0.9471	0.9194	0.7465	0.7649	0.1578	0.9457	0.8699
C/EBPα	0.0295	0.0374	0.0167	0.0294	0.0167	0.0295	0.0167	0.0797	0.0411	0.0169	0.0888	0.1135	0.0876	0.0888	0.0018	0.0151
C/EBPβ	0.0943	0.0945	0.0872	0.0944	0.0863	0.0943	0.0863	0.0873	0.0933	0.0867	0.0533	0.1874	0.1983	0.0533	0.0861	0.1160
Cdx2	0.1213	0.1208	0.1174	0.1206	0.1188	0.1213	0.1188	0.1037	0.1061	0.1182	0.1020	0.6664	0.4983	0.1020	0.1196	0.0265
Gata1/2	0.0358	0.0443	0.0355	0.0356	0.0361	0.0358	0.0361	0.0276	0.0277	0.0351	0.0348	0.3437	0.4495	0.0348	0.0347	0.0008
Gata4/6	0.0373	0.0372	0.0387	0.0372	0.0389	0.0373	0.0389	0.0365	0.0358	0.0388	0.0375	0.4243	0.0694	0.0375	0.0390	0.0001
Hes1	0.0050	0.0157	0.0120	0.0035	0.0264	0.0053	0.0264	0.3517	0.0185	0.0134	0.5737	0.0204	0.0722	0.5737	0.0186	0.0166
Hey2	0.0026	0.0138	0.0094	0.0026	0.0094	0.0026	0.0094	0.3452	0.0158	0.0043	0.3429	0.0084	0.0661	0.3429	0.0023	0.0003
Hoxa10	0.8570	0.5167	0.8735	0.8579	0.8712	0.8568	0.8712	0.8711	0.8570	0.8883	0.7616	0.2217	0.0768	0.7616	0.9134	0.0139
Hoxa3	0.1130	0.1125	0.1094	0.1124	0.1110	0.1131	0.1110	0.0971	0.0993	0.1096	0.1060	0.6328	0.6553	0.1060	0.1111	0.8641
Hoxa5	0.8481	0.3060	0.8529	0.8487	0.8511	0.8480	0.8511	0.0972	0.0972	0.7073	0.0915	0.5373	0.6156	0.0915	0.8651	0.8641
Hoxa9	0.0029	0.0771	0.0093	0.0028	0.0095	0.0029	0.0095	0.8716	0.8666	0.0052	0.5831	0.0209	0.0476	0.5831	0.0023	0.0003
PGC-1	0.4113	0.8718	0.8664	0.8666	0.0065	0.0084	0.0065	0.8897	0.8900	0.8667	0.8784	0.8517	0.1517	0.8784	0.4179	0.0337
PPARγ	0.0011	0.0012	0.0048	0.0011	0.0047	0.0011	0.0047	0.0071	0.0012	0.0065	0.0046	0.0299	0.0245	0.0046	0.0062	0.0021
PRDM14	0.8736	0.8741	0.8777	0.8743	0.8759	0.8735	0.8759	0.8919	0.8895	0.8776	0.8818	0.0664	0.0362	0.8818	0.8759	0.0004
Pu.1	0.0023	0.1912	0.0025	0.0022	0.0027	0.0023	0.0027	0.8870	0.8868	0.0438	0.4511	0.0152	0.0291	0.4511	0.0029	0.0000
Runx1	0.1204	0.4474	0.1249	0.1197	0.1273	0.1204	0.1273	0.9259	0.9237	0.2874	0.9250	0.2290	0.1777	0.9250	0.1299	0.0029
Runx2	0.0601	0.0803	0.1128	0.0599	0.1149	0.0601	0.1149	0.4342	0.1225	0.2863	0.6082	0.7970	0.0482	0.6082	0.1296	0.0159
Sox2	0.9248	0.9249	0.8812	0.9250	0.8793	0.9247	0.8793	0.8982	0.9301	0.8781	0.8820	0.3348	0.1015	0.8820	0.8764	0.0950
Sox4	0.8723	0.5279	0.8673	0.8729	0.8650	0.8722	0.8650	0.0479	0.0367	0.6965	0.4592	0.8618	0.2335	0.4592	0.8623	0.0298
Sox7	0.1166	0.1164	0.1170	0.1163	0.1176	0.1166	0.1176	0.1118	0.1118	0.1211	0.1158	0.8843	0.2121	0.1158	0.1216	0.0129
Sox9	0.1017	0.1014	0.0960	0.1016	0.0962	0.1017	0.0962	0.0931	0.0967	0.0986	0.0939	0.2715	0.1979	0.0939	0.0987	0.1128
IFN-γ	0.4104	0.8819	0.8665	0.8671	0.0215	0.0173	0.0215	0.9353	0.9361	0.8682	0.9004	0.8602	0.8747	0.9004	0.4145	0.3388
IL-1	0.0247	0.0528	0.0825	0.0526	0.0007	0.0005	0.0007	0.0797	0.0525	0.0838	0.0513	0.2516	0.2238	0.0513	0.0397	0.0463
IL-10	0.0000	0.0000	0.0001	0.0000	0.0000	0.0000	0.0000	0.0001	0.0000	0.0001	0.0000	0.0667	0.2716	0.0000	0.0000	0.1163
IL-3	0.0174	0.4726	0.0192	0.0171	0.0203	0.0174	0.0203	0.8882	0.8874	0.1919	0.8879	0.1187	0.0592	0.8879	0.0214	0.0000
IL-6	0.0476	0.0981	0.1106	0.0973	0.0025	0.0025	0.0025	0.1083	0.0987	0.1119	0.1135	0.3244	0.3163	0.1135	0.0566	0.1184
IL-8	0.1139	0.1180	0.1146	0.1178	0.1110	0.1131	0.1110	0.1015	0.1038	0.1147	0.1080	0.8127	0.8463	0.1080	0.1120	0.3514
iκB	0.0002	0.0007	0.0009	0.0006	0.0010	0.0002	0.0010	0.0016	0.0007	0.0013	0.0261	0.0606	0.2246	0.0261	0.0010	0.2136
NF-κB	0.0461	0.0978	0.1103	0.0971	0.0008	0.0009	0.0008	0.1079	0.0984	0.1114	0.1117	0.2005	0.3719	0.1117	0.0540	0.3119
Stat1	0.4077	0.8689	0.8644	0.8663	0.0001	0.0001	0.0001	0.8658	0.8772	0.8588	0.4779	0.8404	0.8622	0.4779	0.4090	0.2754
Stat3	0.8840	0.8855	0.8954	0.8859	0.8904	0.8837	0.8904	0.9379	0.9304	0.8950	0.9071	0.1923	0.1554	0.9071	0.8903	0.0332
Stat5	0.8800	0.4780	0.8663	0.8818	0.8600	0.8797	0.8600	0.0981	0.1076	0.6085	0.0845	0.1452	0.2726	0.0845	0.8533	0.1121
TGF-β	0.0618	0.0588	0.0650	0.0445	0.1207	0.0648	0.1207	0.1152	0.0651	0.0973	0.4351	0.1073	0.0891	0.4351	0.1186	0.1184
TNF-α	0.0011	0.0107	0.0187	0.0105	0.0000	0.0000	0.0000	0.0173	0.0109	0.0193	0.0151	0.1928	0.3209	0.0151	0.0022	0.2298
BMP	0.0645	0.0640	0.5168	0.0644	0.5171	0.0645	0.5171	0.4211	0.0677	0.8897	0.5115	0.8939	0.0570	0.5115	0.8826	0.0000
EGF	0.8926	0.8924	0.8932	0.8926	0.8931	0.8926	0.8931	0.8939	0.8935	0.8931	0.8933	0.1551	0.0000	0.8933	0.8931	0.0000
EGFR	0.9202	0.8931	0.9493	0.9205	0.9490	0.9202	0.9490	0.9448	0.9295	0.9510	0.9457	0.2840	0.0545	0.9457	0.9566	0.0001
FGF2	0.8629	0.5798	0.8695	0.8633	0.8686	0.8628	0.8686	0.8830	0.8629	0.8763	0.8970	0.8559	0.0045	0.8970	0.8840	0.0001
FGF7	0.0002	0.0015	0.0056	0.0015	0.0003	0.0000	0.0003	0.4858	0.0015	0.0752	0.8112	0.8569	0.1008	0.8112	0.0011	0.0010
FGFR2	0.1209	0.1002	0.1198	0.1207	0.1204	0.1210	0.1204	0.1160	0.1153	0.1203	0.1210	0.9439	0.0156	0.1210	0.1212	0.0001
G-CSFR	0.0012	0.0739	0.0133	0.0093	0.0000	0.0003	0.0000	0.8749	0.8747	0.0145	0.4842	0.0869	0.3427	0.4842	0.0016	0.2328
VEGF	0.9552	0.9607	0.9497	0.9607	0.9337	0.9544	0.9337	0.9534	0.9634	0.9476	0.9514	0.3374	0.0261	0.9514	0.9328	0.0023
Akt	0.9630	0.9627	0.9762	0.9632	0.9761	0.9629	0.9761	0.9760	0.9676	0.9763	0.9766	0.3090	0.1620	0.9766	0.9764	0.0515
AP2	0.8676	0.8675	0.2726	0.8676	0.2682	0.8676	0.2682	0.3850	0.8684	0.0067	0.0101	0.5949	0.6589	0.0101	0.0065	0.8644
Bach1	0.8926	0.8924	0.8932	0.8926	0.8933	0.8926	0.8933	0.8941	0.8936	0.8932	0.9019	0.1639	0.0070	0.9019	0.8933	0.0163
c-Jun	0.9434	0.9438	0.9470	0.9434	0.9470	0.9434	0.9470	0.9577	0.9608	0.9477	0.9558	0.4113	0.2991	0.9558	0.9476	0.1093
HIF	0.9401	0.9395	0.9422	0.9402	0.9419	0.9401	0.9419	0.9446	0.9432	0.9419	0.9426	0.2638	0.0129	0.9426	0.9418	0.0002
MAPK	0.9546	0.9594	0.9572	0.9609	0.9291	0.9536	0.9291	0.9478	0.9642	0.9506	0.5270	0.3124	0.3106	0.5270	0.9316	0.0884
p53	0.0375	0.0505	0.0396	0.0393	0.0386	0.0371	0.0386	0.0545	0.0549	0.0460	0.0501	0.1173	0.7799	0.0501	0.0395	0.9109
PKA	0.9431	0.9401	0.9464	0.9431	0.9464	0.9431	0.9464	0.9451	0.9432	0.9462	0.9478	0.3437	0.0085	0.9478	0.9470	0.0021
PTEN	0.1096	0.1090	0.0206	0.1089	0.0201	0.1097	0.0201	0.0393	0.0957	0.0001	0.0001	0.5901	0.5694	0.0001	0.0001	0.7159
HSPG2	0.0045	0.0072	0.0168	0.0030	0.0317	0.0049	0.0317	0.4547	0.0207	0.1961	0.7545	0.8354	0.0081	0.7545	0.0370	0.0164
Integrin	0.9408	0.9412	0.1575	0.9417	0.1593	0.9412	0.1593	0.2969	0.9421	0.0993	0.3938	0.4417	0.3865	0.3938	0.1013	0.1136
NGAL	0.4147	0.8858	0.8671	0.8673	0.0203	0.0172	0.0203	0.9416	0.9415	0.8715	0.9383	0.8677	0.8762	0.9383	0.4233	0.3378
NOG	0.8532	0.8595	0.3309	0.8534	0.3285	0.8532	0.3285	0.3923	0.8572	0.0359	0.1005	0.0181	0.0738	0.1005	0.0354	0.0142
Notch	0.0641	0.1117	0.0981	0.0640	0.0984	0.0641	0.0984	0.3750	0.1171	0.0758	0.3737	0.0948	0.1920	0.3737	0.0611	0.0313
OPN	0.0127	0.0622	0.0047	0.0026	0.0507	0.0219	0.0507	0.1116	0.1103	0.0433	0.1178	0.1168	0.0584	0.1178	0.0329	0.0164
SHH	0.8695	0.8691	0.3640	0.8697	0.3633	0.8695	0.3633	0.4005	0.8748	0.1081	0.2088	0.0251	0.0025	0.2088	0.1103	0.0029
Wnt	0.0983	0.0994	0.1178	0.1037	0.1105	0.0971	0.1105	0.1058	0.0964	0.1162	0.1134	0.0809	0.0598	0.1134	0.1128	0.0166
NR2F2	0.8681	0.8680	0.3327	0.8682	0.3302	0.8681	0.3302	0.3955	0.8702	0.0276	0.0955	0.0054	0.0021	0.0955	0.0270	0.0000
RARs	0.0534	0.0530	0.0166	0.0528	0.0172	0.0535	0.0172	0.0359	0.0524	0.0000	0.0000	0.5196	0.5702	0.0000	0.0000	0.8600

(*Continued on next page*)

Supplementary Table VI. Saddle Points from Differential Equations.

Connected Attractors & Times (Tot. 500 perturbations)	Saddle 17	Saddle 18	Saddle 19	Saddle 20	Saddle 21	Saddle 22	Saddle 23	Saddle 24	Saddle 25	Saddle 26	Saddle 27	Saddle 28	Saddle 29	Saddle 30	Saddle 31	Saddle 32
	S5 & S18	S6 & S7	S6 & S18	S7 & S8	S7 & S16	S8 & S12	S8 & S17	S9 & S10	S10 & S11	S12 & S13	S12 & S14	S12 & S15	S13 & S14	S13 & S15	S16 & S17	S16 & S18
	258;242	253;247	268;232	257;243	233;267	260;240	239;261	236;264	252;248	269;231	256;244	241;259	255;245	269;231	261;239	248;252
Cdk2	0.8895	0.8261	0.8873	0.0160	0.0160	0.2011	0.0160	0.8970	0.8963	0.0001	0.0015	0.0004	0.0001	0.0000	0.0160	0.8261
Cdk6	0.8890	0.8255	0.8868	0.8605	0.8605	0.2012	0.8605	0.8971	0.8960	0.0000	0.0010	0.0001	0.0001	0.0000	0.8605	0.8255
Cyclin D	0.9618	0.9542	0.9616	0.9028	0.9370	0.1522	0.8962	0.8703	0.8855	0.0000	0.0000	0.0000	0.0000	0.0000	0.9028	0.9542
Cyclin E	0.9599	0.9525	0.9598	0.8980	0.9347	0.1258	0.8908	0.8545	0.8594	0.0000	0.0000	0.0000	0.0000	0.0000	0.8980	0.9525
E2F	0.9441	0.9281	0.9436	0.4063	0.8659	0.0054	0.0145	0.0203	0.0202	0.0000	0.0000	0.0000	0.0000	0.0000	0.4063	0.9281
Myc	0.9461	0.7895	0.9408	0.1139	0.1158	0.0953	0.1136	0.1275	0.1275	0.0014	0.0024	0.0005	0.0011	0.0001	0.1139	0.7895
p21	0.0009	0.0114	0.0012	0.0475	0.0475	0.3900	0.0475	0.0872	0.0872	0.9398	0.9408	0.9268	0.9540	0.9394	0.0475	0.0114
p27	0.0220	0.0179	0.0012	0.0475	0.0475	0.3967	0.0475	0.0873	0.0412	0.9308	0.9429	0.8883	0.9370	0.9001	0.0475	0.0179
pRb	0.9695	0.9662	0.9694	0.9545	0.9579	0.1790	0.9538	0.9646	0.9651	0.0000	0.0000	0.0000	0.0000	0.0000	0.9545	0.9662
Ras	0.9435	0.9420	0.9433	0.9458	0.9458	0.8840	0.9458	0.9400	0.9424	0.0647	0.0591	0.0430	0.0017	0.0016	0.9458	0.9420
Bad	0.0000	0.0001	0.0000	0.0000	0.0000	0.0041	0.0000	0.0001	0.0000	0.0814	0.0825	0.0961	0.0921	0.0959	0.0000	0.0001
Bax	0.0001	0.0002	0.0001	0.0005	0.0005	0.0872	0.0005	0.0001	0.0001	0.6744	0.6826	0.8587	0.9480	0.9479	0.0005	0.0003
Bcl-2	0.1504	0.7356	0.1726	0.1144	0.0816	0.3595	0.0816	0.9420	0.9419	0.0001	0.0040	0.0000	0.0003	0.0000	0.0707	0.1389
Bcl-xL	0.1588	0.9133	0.1826	0.8978	0.2079	0.3579	0.2079	0.8916	0.9359	0.0605	0.0693	0.1296	0.0027	0.0000	0.1474	0.1472
Bim	0.0544	0.0664	0.0549	0.0960	0.0960	0.5871	0.0960	0.0533	0.0537	0.9992	0.9887	0.9992	0.9991	1.0000	0.0960	0.0664
Caspase 3	0.7886	0.0000	0.7448	0.0000	0.6922	0.0001	0.6923	0.0000	0.0000	0.0014	0.0015	0.0023	0.0021	0.0023	0.7985	0.8043
Cytochrome C	0.7734	0.0000	0.7238	0.0000	0.7008	0.0026	0.7008	0.0000	0.0000	0.0811	0.0816	0.0962	0.0924	0.0963	0.8063	0.7923
Caspase 8	0.0000	0.0000	0.0000	0.0000	0.0000	0.0005	0.0000	0.0000	0.0000	0.0000	0.0002	0.0000	0.0000	0.0000	0.0000	0.0000
Fas	0.0000	0.0000	0.0000	0.0000	0.0000	0.0001	0.0000	0.0000	0.0000	0.0000	0.0002	0.0000	0.0000	0.0000	0.0000	0.0000
XIAP	0.1602	0.9338	0.1842	0.9457	0.2190	0.7317	0.2190	0.9631	0.9629	0.8668	0.8629	0.8669	0.8667	0.8670	0.1552	0.1505
C/EBPα	0.0018	0.0175	0.0018	0.0567	0.0567	0.0781	0.0567	0.0374	0.0295	0.0299	0.0312	0.0131	0.0321	0.0201	0.0567	0.0175
C/EBPβ	0.0861	0.0785	0.0858	0.0009	0.0009	0.1603	0.0009	0.0945	0.0943	0.1151	0.1169	0.1151	0.1153	0.1151	0.0009	0.0785
Cdx2	0.1196	0.1151	0.1198	0.0606	0.0606	0.4808	0.0606	0.1208	0.1213	0.8292	0.8377	0.2117	0.8213	0.4211	0.0606	0.1151
Gata1/2	0.0347	0.0369	0.0348	0.0281	0.0281	0.1903	0.0281	0.0443	0.0358	0.8491	0.8531	0.0726	0.8472	0.4280	0.0281	0.0369
Gata4/6	0.0390	0.0389	0.0390	0.0301	0.0301	0.2239	0.0301	0.0372	0.0373	0.2950	0.2985	0.0930	0.0000	0.0000	0.0301	0.0389
Hes1	0.0186	0.1819	0.0238	0.9313	0.9313	0.3190	0.9313	0.0157	0.0050	0.0303	0.0204	0.0184	0.0306	0.0208	0.9313	0.1819
Hey2	0.0023	0.0118	0.0023	0.8632	0.8632	0.0497	0.8632	0.0138	0.0026	0.0136	0.0143	0.0008	0.0163	0.0019	0.8632	0.0118
Hoxa10	0.9134	0.8486	0.9127	0.8595	0.8595	0.3463	0.8595	0.5167	0.8570	0.0559	0.0543	0.2732	0.0000	0.0000	0.8595	0.8486
Hoxa3	0.1111	0.1102	0.1113	0.1089	0.1089	0.6135	0.1089	0.1125	0.1130	0.8668	0.8618	0.8669	0.8666	0.8670	0.1089	0.1102
Hoxa5	0.8651	0.6885	0.8646	0.0943	0.0943	0.5524	0.0943	0.3060	0.8481	0.8515	0.8463	0.8703	0.8509	0.8625	0.0943	0.6885
Hoxa9	0.0023	0.0126	0.0023	0.8513	0.8513	0.0531	0.8513	0.0771	0.0029	0.0139	0.0144	0.0009	0.0166	0.0020	0.8513	0.0126
PGC-1	0.4179	0.3629	0.0064	0.8758	0.8758	0.4567	0.8758	0.8718	0.4113	0.0225	0.1889	0.0026	0.0454	0.0016	0.8758	0.3629
PPARγ	0.0062	0.0040	0.0061	0.0002	0.0002	0.0168	0.0002	0.0012	0.0011	0.0020	0.0022	0.0020	0.0021	0.0020	0.0002	0.0040
PRDM14	0.8759	0.8768	0.8756	0.8789	0.8789	0.1071	0.8789	0.8741	0.8736	0.0001	0.0007	0.0001	0.0001	0.0000	0.8789	0.8768
Pu.1	0.0029	0.0435	0.0029	0.1147	0.1147	0.0159	0.1147	0.1912	0.0023	0.0024	0.0025	0.0000	0.0026	0.0030	0.1147	0.0435
Runx1	0.1299	0.2917	0.1303	0.9344	0.9344	0.2273	0.9344	0.4474	0.1204	0.1217	0.1239	0.0128	0.1227	0.0807	0.9344	0.2917
Runx2	0.1296	0.2835	0.1299	0.1306	0.1306	0.5899	0.1306	0.0803	0.0601	0.3328	0.3324	0.2837	0.0314	0.0236	0.1306	0.2835
Sox2	0.8764	0.8772	0.8761	0.8785	0.8785	0.5677	0.8785	0.9249	0.9248	0.0938	0.0934	0.0976	0.0895	0.0933	0.8785	0.8772
Sox4	0.8623	0.7095	0.8619	0.8778	0.8778	0.8338	0.8778	0.5279	0.8723	0.3354	0.3303	0.2128	0.0148	0.0190	0.8778	0.7095
Sox7	0.1216	0.1196	0.1217	0.1154	0.1154	0.7673	0.1154	0.1164	0.1166	0.3484	0.3503	0.2137	0.0001	0.0000	0.1154	0.1196
Sox9	0.0987	0.0977	0.0987	0.0951	0.0951	0.2668	0.0951	0.1014	0.1017	0.1167	0.1199	0.1161	0.1156	0.1154	0.0951	0.0977
IFN-γ	0.4145	0.3838	0.0239	0.8913	0.8913	0.4333	0.8913	0.8819	0.4104	0.2056	0.7811	0.0716	0.4143	0.0054	0.8913	0.3838
IL-1	0.0397	0.0267	0.0008	0.0041	0.0041	0.1304	0.0041	0.0528	0.0247	0.0271	0.1052	0.0112	0.0550	0.0020	0.0041	0.0267
IL-10	0.0000	0.0000	0.0000	0.0000	0.0000	0.0005	0.0000	0.0000	0.0000	0.0000	0.0002	0.0000	0.0000	0.0000	0.0000	0.0000
IL-3	0.0214	0.1989	0.0216	0.8908	0.8908	0.1089	0.8908	0.4726	0.0174	0.0180	0.0189	0.0000	0.0184	0.0053	0.8908	0.1989
IL-6	0.0566	0.0619	0.0026	0.1147	0.1147	0.1928	0.1147	0.0981	0.0476	0.1154	0.1269	0.1153	0.1171	0.1152	0.1147	0.0619
IL-8	0.1120	0.1107	0.1114	0.1140	0.1140	0.5426	0.1140	0.1180	0.1139	0.0197	0.7708	0.0176	0.4130	0.0190	0.1140	0.1107
iκB	0.0010	0.0100	0.0012	0.0501	0.0501	0.1772	0.0501	0.0007	0.0002	0.0171	0.0230	0.0176	0.0173	0.0190	0.0501	0.0100
NF-κB	0.0540	0.0468	0.0008	0.1126	0.1126	0.1169	0.1126	0.0978	0.0461	0.0138	0.1202	0.0168	0.0644	0.0085	0.1126	0.0468
Stat1	0.4090	0.2545	0.0001	0.1060	0.1060	0.2337	0.1060	0.8689	0.4077	0.0637	0.6944	0.0033	0.4094	0.0000	0.1060	0.2545
Stat3	0.8903	0.8928	0.8895	0.8987	0.8987	0.2289	0.8987	0.8855	0.8840	0.0178	0.0407	0.0223	0.0193	0.0009	0.8987	0.8928
Stat5	0.8533	0.6041	0.8521	0.1025	0.1025	0.2799	0.1025	0.4780	0.8800	0.1857	0.1783	0.2460	0.0001	0.0000	0.1025	0.6041
TGF-β	0.1186	0.2761	0.1302	0.8984	0.8984	0.3465	0.8984	0.0588	0.0618	0.1203	0.0853	0.1213	0.1144	0.1245	0.8984	0.2761
TNF-α	0.0022	0.0012	0.0000	0.0141	0.0141	0.0368	0.0141	0.0107	0.0011	0.0002	0.0282	0.0001	0.0043	0.0000	0.0141	0.0012
BMP	0.8826	0.7371	0.8778	0.1144	0.1144	0.6291	0.1144	0.0640	0.0645	0.3767	0.3778	0.3007	0.0179	0.0051	0.1144	0.7371
EGF	0.8931	0.8931	0.8931	0.8934	0.8934	0.3016	0.8934	0.8924	0.8926	0.0000	0.0000	0.0000	0.0000	0.0000	0.8934	0.8931
EGFR	0.9566	0.9407	0.9563	0.9567	0.9567	0.3182	0.9567	0.8931	0.9202	0.0336	0.0340	0.0098	0.0020	0.0006	0.9567	0.9407
FGF2	0.8840	0.8647	0.8838	0.9293	0.9293	0.8008	0.9293	0.5798	0.8629	0.1788	0.1685	0.2193	0.0000	0.0001	0.9293	0.8647
FGF7	0.0011	0.2206	0.0007	0.8717	0.8717	0.7839	0.8717	0.0015	0.0002	0.1777	0.1754	0.0715	0.0017	0.0001	0.8717	0.2206
FGFR2	0.1212	0.1199	0.1213	0.1226	0.1226	0.9142	0.1226	0.1002	0.1209	0.3260	0.3201	0.3511	0.0003	0.0001	0.1226	0.1199
G-CSFR	0.0016	0.0019	0.0000	0.0302	0.0302	0.0204	0.0302	0.0739	0.0012	0.0003	0.0173	0.0001	0.0030	0.0001	0.0302	0.0019
VEGF	0.9328	0.9332	0.9302	0.9591	0.9591	0.2821	0.9591	0.9607	0.9552	0.0286	0.0349	0.0034	0.0021	0.0025	0.9591	0.9332
Akt	0.9764	0.9761	0.9763	0.9798	0.9798	0.3498	0.9798	0.9627	0.9630	0.0316	0.0329	0.0338	0.0021	0.0002	0.9798	0.9761
AP2	0.0065	0.0068	0.0065	0.0002	0.0002	0.5533	0.0002	0.8675	0.8676	0.8671	0.8627	0.8671	0.8669	0.8673	0.0002	0.0068
Bach1	0.8933	0.8954	0.8933	0.9399	0.9399	0.4589	0.9399	0.8924	0.8926	0.0171	0.0062	0.0175	0.0148	0.0190	0.9399	0.8954
c-Jun	0.9476	0.9470	0.9476	0.9616	0.9616	0.4625	0.9616	0.9438	0.9434	0.0184	0.0196	0.0004	0.0181	0.0052	0.9616	0.9470
HIF	0.9418	0.9418	0.9418	0.9429	0.9429	0.3508	0.9429	0.9395	0.9401	0.0001	0.0002	0.0001	0.0000	0.0000	0.9429	0.9418
MAPK	0.9316	0.7811	0.9245	0.1176	0.1176	0.3020	0.1176	0.9594	0.9546	0.0360	0.1034	0.0339	0.0454	0.0000	0.1176	0.7811
p53	0.0395	0.0436	0.0394	0.0365	0.0365	0.0967	0.0365	0.0505	0.0375	0.6776	0.6964	0.8528	0.9388	0.9363	0.0365	0.0436
PKA	0.9470	0.9461	0.9470	0.9617	0.9617	0.5336	0.9617	0.9401	0.9431	0.0043	0.0016	0.0025	0.0016	0.0021	0.9617	0.9461
PTEN	0.0001	0.0001	0.0001	0.0001	0.0001	0.5546	0.0001	0.1090	0.1096	0.9059	0.8913	0.9270	0.9341	0.9364	0.0001	0.0001
HSPG2	0.0370	0.3047	0.0422	0.8791	0.8791	0.7117	0.8791	0.0072	0.0045	0.2785	0.2719	0.1975	0.0151	0.0191	0.8791	0.3047
Integrin	0.1013	0.1609	0.1028	0.9274	0.9267	0.4328	0.9275	0.9417	0.9413	0.0007	0.0116	0.0004	0.0009	0.0000	0.9274	0.1609
NGAL	0.4233	0.4487	0.0218	0.9384	0.9384	0.4823	0.9384	0.8858	0.4147	0.0950	0.8272	0.0037	0.4219	0.0052	0.9384	0.4487
NOG	0.0354	0.0656	0.0355	0.0356	0.0356	0.0215	0.0356	0.8595	0.8532	0.0121	0.0133	0.0148	0.0152	0.0151	0.0356	0.0656
Notch	0.0611	0.1061	0.0616	0.8576	0.8576	0.1735	0.8576	0.1117	0.0641	0.1113	0.1133	0.0439	0.1183	0.0576	0.8576	0.1061
OPN	0.0329	0.2754	0.0621	0.1217	0.1217	0.3693	0.1217	0.0622	0.0127	0.2877	0.2641	0.1975	0.0326	0.0241	0.1217	0.2754
SHH	0.1103	0.1721	0.1119	0.1026	0.1026	0.1402	0.1026	0.8691	0.8695	0.0024	0.0013	0.0035	0.0015	0.0027	0.1026	0.1721
Wnt	0.1128	0.1039	0.1114	0.1222	0.1222	0.1015	0.1222	0.0994	0.0983	0.0396	0.0413	0.0842	0.0024	0.0107	0.1222	0.1039
NR2F2	0.0270	0.0585	0.0271	0.0282	0.0282	0.0366	0.0282	0.8680	0.8681	0.0006	0.0007	0.0059	0.0000	0.0000	0.0282	0.0585
RARs	0.0000	0.0000	0.0000	0.0000	0.0000	0.4928	0.0000	0.0530	0.0534	0.8666	0.8558	0.8667	0.8661	0.8671	0.0000	0.0000

Supplementary Table VII. Unstable Fixed Points from Differential Equations with Two or More Unstable Eigenvalues (positive real part). These 25 unstable points are first obtained while calculating with 100,000 random initial positions using *Newtonian iteration method*. We further run the program with 1,000,000 initial positions, acquiring an identical result.

Unstable Eigenvalues (positive real part)	2	2	2	2	2	2	2	2	2	2	2	2	2	2	2	2	2	2	2	3	3	3	3	3	4
Cdk2	0.8905	0.8905	0.8963	0.8970	0.8947	0.8887	0.8395	0.0128	0.0013	0.1915	0.1165	0.5898	0.8395	0.8895	0.8261	0.0160	0.1973	0.0004	0.0004	0.8905	0.0075	0.8395	0.1811	0.1726	0.1738
Cdk6	0.8900	0.8900	0.8960	0.8971	0.8943	0.8882	0.8388	0.0021	0.0002	0.1914	0.0747	0.8122	0.8388	0.8890	0.8255	0.8605	0.1965	0.0003	0.0001	0.8900	0.0017	0.8388	0.1800	0.1715	0.1725
Cyclin D	0.9550	0.9550	0.8935	0.8796	0.9524	0.9552	0.9553	0.0000	0.0000	0.0924	0.0057	0.9423	0.9553	0.9618	0.9542	0.9028	0.1272	0.0000	0.0000	0.9550	0.0000	0.9553	0.0833	0.1068	0.0844
Cyclin E	0.9525	0.9525	0.8686	0.8637	0.9510	0.9526	0.9537	0.0000	0.0000	0.0552	0.0002	0.9395	0.9537	0.9599	0.9525	0.8980	0.0920	0.0000	0.0000	0.9525	0.0000	0.9537	0.0346	0.0365	0.0218
E2F	0.9288	0.9288	0.4065	0.4065	0.9288	0.9287	0.9310	0.0000	0.0000	0.0028	0.0004	0.8952	0.9310	0.9441	0.9281	0.4063	0.0040	0.0000	0.0000	0.9288	0.0000	0.9310	0.0026	0.0036	0.0030
Myc	0.7956	0.7956	0.1276	0.1277	0.7958	0.7947	0.8155	0.0022	0.0003	0.0837	0.0636	0.5192	0.8155	0.9461	0.7895	0.1139	0.0898	0.0016	0.0015	0.7956	0.0028	0.8155	0.0841	0.0905	0.0881
p21	0.0113	0.0113	0.0872	0.0872	0.0114	0.0114	0.0094	0.9159	0.9386	0.4814	0.7930	0.0334	0.0094	0.0009	0.0114	0.0475	0.4304	0.9401	0.9171	0.0113	0.9109	0.0094	0.5021	0.4747	0.5047
p27	0.0268	0.0268	0.0412	0.0873	0.0563	0.0012	0.0226	0.7957	0.8791	0.5172	0.8515	0.0483	0.0226	0.0220	0.0179	0.0475	0.4404	0.9333	0.8870	0.0268	0.8361	0.0226	0.5429	0.4958	0.5539
pRb	0.9692	0.9692	0.9656	0.9651	0.9693	0.9691	0.9669	0.0000	0.0000	0.1304	0.0196	0.9601	0.9669	0.9695	0.9662	0.9545	0.1532	0.0000	0.0000	0.9692	0.0000	0.9669	0.1103	0.1027	0.0990
Ras	0.9432	0.9432	0.9424	0.9400	0.9429	0.9430	0.9422	0.0464	0.0018	0.8886	0.1682	0.9436	0.9422	0.9435	0.9420	0.9458	0.8788	0.0632	0.0593	0.9432	0.0648	0.9422	0.8688	0.7283	0.7546
Bad	0.0000	0.0000	0.0000	0.0001	0.0001	0.0000	0.0001	0.0937	0.0948	0.0063	0.0876	0.0001	0.0001	0.0000	0.0001	0.0000	0.0038	0.0816	0.0936	0.0000	0.0914	0.0001	0.0044	0.0033	0.0044
Bax	0.0001	0.0001	0.0001	0.0001	0.0001	0.0001	0.0002	0.8251	0.9471	0.0849	0.4239	0.0006	0.0003	0.0001	0.0003	0.0005	0.0867	0.6766	0.7620	0.0001	0.7417	0.0003	0.0828	0.0915	0.0853
Bcl-2	0.8794	0.1509	0.9419	0.9420	0.8869	0.1725	0.7591	0.0354	0.0112	0.4097	0.2273	0.1608	0.1411	0.1695	0.1760	0.0816	0.3381	0.0009	0.0000	0.1695	0.0301	0.1753	0.3857	0.2354	0.3757
Bcl-xL	0.9372	0.1589	0.9359	0.8916	0.9086	0.1821	0.9137	0.3297	0.1006	0.2843	0.3320	0.1869	0.1487	0.1791	0.1882	0.2079	0.4343	0.0618	0.1259	0.1786	0.2550	0.1861	0.4997	0.6285	0.6038
Bim	0.0541	0.0541	0.0537	0.0533	0.0529	0.0546	0.0643	0.9814	0.9977	0.6024	0.7389	0.0849	0.0643	0.0544	0.0664	0.0960	0.5972	0.9967	0.9990	0.0541	0.9880	0.0643	0.6128	0.6283	0.6250
Caspase 3	0.0000	0.7882	0.0000	0.0000	0.0000	0.7457	0.0000	0.0142	0.0036	0.0013	0.0518	0.7234	0.8012	0.7510	0.7276	0.6923	0.0001	0.0014	0.0021	0.7517	0.0062	0.7312	0.0007	0.0000	0.0007
Cytochrome C	0.0000	0.7728	0.0000	0.0000	0.0000	0.7247	0.0000	0.0939	0.0964	0.0020	0.0672	0.7145	0.7891	0.7313	0.7080	0.7008	0.0022	0.0812	0.0934	0.7320	0.0921	0.7121	0.0014	0.0016	0.0012
Caspase 8	0.0000	0.0000	0.0000	0.0000	0.0001	0.0000	0.0000	0.0742	0.0094	0.0060	0.1827	0.0000	0.0000	0.0000	0.0000	0.0000	0.0006	0.0000	0.0000	0.0000	0.0280	0.0000	0.0031	0.0000	0.0028
Fas	0.0000	0.0000	0.0000	0.0000	0.0000	0.0000	0.0000	0.0886	0.0105	0.0008	0.1948	0.0000	0.0000	0.0000	0.0000	0.0000	0.0001	0.0000	0.0000	0.0000	0.0325	0.0000	0.0004	0.0000	0.0005
XIAP	0.9460	0.1604	0.9629	0.9631	0.9472	0.1837	0.9358	0.8671	0.8681	0.7299	0.7468	0.1921	0.1523	0.1806	0.1925	0.2190	0.7152	0.8659	0.8668	0.1803	0.8667	0.1906	0.7107	0.6608	0.6940
C/EBPα	0.0167	0.0167	0.0295	0.0374	0.0369	0.0167	0.0173	0.0129	0.0201	0.1002	0.1197	0.0888	0.0173	0.0018	0.0175	0.0567	0.0657	0.0302	0.0143	0.0167	0.0144	0.0173	0.0601	0.0358	0.0428
C/EBPβ	0.0865	0.0865	0.0943	0.0945	0.0870	0.0863	0.0801	0.1168	0.1155	0.1746	0.2034	0.0533	0.0801	0.0861	0.0785	0.0009	0.1685	0.1155	0.1151	0.0865	0.1163	0.0801	0.1832	0.1781	0.1856
Cdx2	0.1186	0.1186	0.1213	0.1208	0.1173	0.1188	0.1161	0.1866	0.4196	0.5429	0.5378	0.1020	0.1161	0.1196	0.1151	0.0606	0.4987	0.8317	0.3525	0.1186	0.3520	0.1161	0.5572	0.5181	0.5555
Gata1/2	0.0361	0.0361	0.0358	0.0443	0.0397	0.0361	0.0366	0.0507	0.4257	0.2592	0.4642	0.0348	0.0366	0.0347	0.0369	0.0281	0.2196	0.8503	0.2661	0.0361	0.2629	0.0366	0.2908	0.2735	0.3002
Gata4/6	0.0389	0.0389	0.0373	0.0372	0.0387	0.0389	0.0390	0.0653	0.0000	0.3339	0.3192	0.0375	0.0390	0.0390	0.0389	0.0301	0.2753	0.2961	0.1873	0.0389	0.1869	0.0390	0.3787	0.3802	0.4119
Hes1	0.0225	0.0225	0.0050	0.0157	0.0753	0.0264	0.1547	0.0171	0.0174	0.1987	0.0759	0.5737	0.1547	0.0186	0.1819	0.9313	0.2651	0.0275	0.0191	0.0225	0.0176	0.1547	0.1439	0.1597	0.1114
Hey2	0.0094	0.0094	0.0026	0.0138	0.0673	0.0094	0.0100	0.0016	0.0024	0.0248	0.0693	0.3429	0.0100	0.0023	0.0118	0.8632	0.0353	0.0138	0.0021	0.0094	0.0028	0.0100	0.0181	0.0274	0.0213
Hoxa10	0.8715	0.8715	0.8570	0.5167	0.7427	0.8712	0.8560	0.2880	0.0000	0.3085	0.1401	0.7616	0.8560	0.9134	0.8486	0.8595	0.3463	0.0554	0.2459	0.8715	0.2528	0.8560	0.3071	0.3365	0.3035
Hoxa3	0.1108	0.1108	0.1130	0.1125	0.1094	0.1110	0.1105	0.8613	0.8659	0.6180	0.6425	0.1060	0.1105	0.1111	0.1102	0.1089	0.6082	0.8657	0.8669	0.1108	0.8630	0.1105	0.6068	0.5951	0.6013
Hoxa5	0.8514	0.8514	0.8481	0.3060	0.5649	0.8511	0.6927	0.8660	0.8616	0.5505	0.5865	0.0915	0.6927	0.8651	0.6885	0.0943	0.5655	0.8503	0.8682	0.8514	0.8651	0.6927	0.5636	0.5689	0.5630
Hoxa9	0.0095	0.0095	0.0029	0.0771	0.0720	0.0095	0.0108	0.0013	0.0022	0.0325	0.0620	0.5831	0.0108	0.0023	0.0126	0.8513	0.0372	0.0140	0.0021	0.0095	0.0024	0.0108	0.0194	0.0277	0.0213
PGC-1	0.4164	0.4164	0.4113	0.8718	0.8680	0.0065	0.4304	0.0490	0.0432	0.5946	0.4006	0.8784	0.4304	0.4179	0.3629	0.8758	0.4279	0.1155	0.0056	0.4164	0.0752	0.4304	0.5228	0.1976	0.4791
PPARγ	0.0047	0.0047	0.0011	0.0012	0.0051	0.0047	0.0044	0.0021	0.0020	0.0236	0.0311	0.0046	0.0044	0.0062	0.0040	0.0002	0.0191	0.0021	0.0020	0.0047	0.0021	0.0044	0.0249	0.0240	0.0267
PRDM14	0.8762	0.8762	0.8736	0.8741	0.8777	0.8759	0.8765	0.0009	0.0002	0.0872	0.0376	0.8818	0.8765	0.8759	0.8768	0.8789	0.0979	0.0002	0.0001	0.8762	0.0007	0.8765	0.0789	0.0661	0.0666
Pu.1	0.0027	0.0027	0.0023	0.1912	0.0843	0.0027	0.0433	0.0000	0.0030	0.0154	0.0310	0.4511	0.0433	0.0029	0.0435	0.1147	0.0122	0.0024	0.0010	0.0027	0.0009	0.0433	0.0092	0.0057	0.0064
Runx1	0.1269	0.1269	0.1204	0.4474	0.3484	0.1273	0.2904	0.0104	0.0805	0.2255	0.1987	0.9250	0.2904	0.1299	0.2917	0.9344	0.2104	0.1222	0.0509	0.1269	0.0510	0.2904	0.1955	0.1647	0.1727
Runx2	0.1146	0.1146	0.0601	0.0803	0.2735	0.1149	0.2837	0.2569	0.0198	0.7198	0.2730	0.6082	0.2837	0.1296	0.2835	0.1306	0.5184	0.3328	0.3072	0.1146	0.3029	0.2837	0.4717	0.3109	0.3486
Sox2	0.8796	0.8796	0.9248	0.9249	0.8810	0.8793	0.8769	0.1004	0.0935	0.4303	0.1233	0.8820	0.8769	0.8764	0.8772	0.8785	0.5011	0.0937	0.1004	0.8796	0.1021	0.8769	0.3942	0.3820	0.3535
Sox4	0.8653	0.8653	0.8723	0.5279	0.6323	0.8650	0.7075	0.2340	0.0151	0.8452	0.4335	0.4592	0.7075	0.8623	0.7095	0.8778	0.8472	0.3340	0.2829	0.8653	0.2933	0.7075	0.8672	0.8589	0.8652
Sox7	0.1175	0.1175	0.1166	0.1164	0.1170	0.1176	0.1200	0.1795	0.0000	0.8213	0.3982	0.1158	0.1200	0.1216	0.1196	0.1154	0.7868	0.3490	0.2791	0.1175	0.2769	0.1200	0.8335	0.7691	0.8011
Sox9	0.0962	0.0962	0.1017	0.1014	0.0961	0.0962	0.0978	0.1158	0.1144	0.2754	0.2199	0.0939	0.0978	0.0987	0.0977	0.0951	0.2787	0.1174	0.1164	0.0962	0.1165	0.0978	0.2934	0.3049	0.2983
IFN-γ	0.4136	0.4136	0.4104	0.8819	0.8716	0.0215	0.4220	0.3478	0.3963	0.5261	0.8638	0.9004	0.4220	0.4145	0.3838	0.8913	0.4145	0.4506	0.1170	0.4136	0.3860	0.4220	0.4728	0.2680	0.4438
IL-1	0.0389	0.0389	0.0247	0.0528	0.0823	0.0007	0.0319	0.0479	0.0533	0.1566	0.2342	0.0513	0.0319	0.0397	0.0267	0.0041	0.1279	0.0600	0.0170	0.0389	0.0526	0.0319	0.1483	0.0965	0.1489
IL-10	0.0000	0.0000	0.0000	0.0000	0.0001	0.0000	0.0000	0.0937	0.0105	0.0064	0.2234	0.0000	0.0000	0.0000	0.0000	0.0000	0.0006	0.0000	0.0000	0.0000	0.0328	0.0000	0.0032	0.0000	0.0029
IL-3	0.0201	0.0201	0.0174	0.4726	0.2975	0.0203	0.1968	0.0000	0.0053	0.1109	0.0873	0.8879	0.1968	0.0214	0.1989	0.8908	0.0876	0.0182	0.0013	0.0201	0.0014	0.1968	0.0714	0.0432	0.0497
IL-6	0.0557	0.0557	0.0476	0.0981	0.1111	0.0025	0.0721	0.1205	0.1173	0.2668	0.3330	0.1135	0.0721	0.0566	0.0619	0.1147	0.2198	0.1185	0.1154	0.0557	0.1194	0.0721	0.2768	0.2616	0.2947
IL-8	0.1116	0.1116	0.1139	0.1180	0.1146	0.1110	0.1112	0.3415	0.3998	0.5911	0.8341	0.1080	0.1112	0.1120	0.1107	0.1140	0.5137	0.3718	0.0172	0.1116	0.3449	0.1112	0.5562	0.4257	0.5485
iκB	0.0008	0.0008	0.0002	0.0007	0.0013	0.0010	0.0085	0.2019	0.1117	0.1451	0.1949	0.0261	0.0085	0.0010	0.0100	0.0501	0.1611	0.0176	0.0171	0.0008	0.1539	0.0085	0.1119	0.1018	0.0903
NF-κB	0.0533	0.0533	0.0461	0.0978	0.1106	0.0008	0.0555	0.3012	0.2237	0.1775	0.3299	0.1117	0.0555	0.0540	0.0468	0.1126	0.1239	0.0714	0.0210	0.0533	0.2596	0.0555	0.1596	0.0693	0.1560
Stat1	0.4088	0.4088	0.4077	0.8689	0.8596	0.0001	0.3360	0.2811	0.3778	0.4247	0.8378	0.4779	0.3360	0.4090	0.2545	0.1060	0.2316	0.3858	0.0137	0.4088	0.3229	0.3360	0.4039	0.1100	0.4031
Stat3	0.8912	0.8912	0.8840	0.8855	0.8953	0.8904	0.8919	0.0440	0.0282	0.2122	0.1576	0.9071	0.8919	0.8903	0.8928	0.8987	0.2214	0.0250	0.0245	0.8912	0.0417	0.8919	0.2046	0.1920	0.1926
Stat5	0.8610	0.8610	0.8800	0.4780	0.5520	0.8600	0.6053	0.2796	0.0101	0.1846	0.2093	0.0845	0.6053	0.8533	0.6041	0.1025	0.3545	0.1835	0.2432	0.8610	0.2556	0.6053	0.4095	0.5172	0.4928
TGF-β	0.1106	0.1106	0.0618	0.0588	0.0976	0.1207	0.2586	0.1163	0.1152	0.2813	0.0917	0.4351	0.2586	0.1186	0.2761	0.8984	0.3189	0.1127	0.1202	0.1106	0.1146	0.2586	0.2464	0.2529	0.2180
TNF-α	0.0021	0.0021	0.0011	0.0107	0.0188	0.0000	0.0020	0.2135	0.1019	0.0862	0.2949	0.0151	0.0020	0.0022	0.0012	0.0141	0.0384	0.0058	0.0001	0.0021	0.1498	0.0020	0.0683	0.0122	0.0663
BMP	0.5169	0.5169	0.0645	0.0640	0.5163	0.5171	0.7620	0.2761	0.0051	0.7355	0.3436	0.5115	0.7620	0.8826	0.7371	0.1144	0.6696	0.3770	0.3383	0.5169	0.3369	0.7620	0.7654	0.6475	0.7021
EGF	0.8931	0.8931	0.8926	0.8924	0.8931	0.8931	0.8931	0.0000	0.0000	0.2338	0.0005	0.8933	0.8931	0.8931	0.8931	0.8934	0.2672	0.0000	0.0000	0.8931	0.0000	0.8931	0.2050	0.1849	0.1749
EGFR	0.9491	0.9491	0.9202	0.8931	0.9379	0.9490	0.9423	0.0238	0.0008	0.3031	0.1680	0.9457	0.9423	0.9566	0.9407	0.9567	0.3119	0.0336	0.0214	0.9491	0.0357	0.9423	0.3006	0.3123	0.3071
FGF2	0.8688	0.8688	0.8629	0.5798	0.8059	0.8686	0.8668	0.2170	0.0000	0.8432	0.0745	0.8970	0.8668	0.8840	0.8647	0.9293	0.7462	0.1762	0.2178	0.8688	0.2174	0.8668	0.6565	0.4108	0.4087
FGF7	0.0008	0.0008	0.0002	0.0015	0.0568	0.0003	0.1904	0.0378	0.0015	0.8366	0.1537	0.8112	0.1904	0.0011	0.2206	0.8717	0.7179	0.1765	0.1149	0.0008	0.1053	0.1904	0.6233	0.2454	0.3078
FGFR2	0.1203	0.1203	0.1209	0.1002	0.1166	0.1204	0.1201	0.3382	0.0001	0.9345	0.2134	0.1210	0.1201	0.1212	0.1199	0.1226	0.8978	0.3243	0.3577	0.1203	0.3564	0.1201	0.8640	0.6018	0.6247
G-CSFR	0.0016	0.0016	0.0012	0.0739	0.0196	0.0000	0.0026	0.2146	0.1007	0.0619	0.2735	0.4842	0.0026	0.0016	0.0019	0.0302	0.0214	0.0039	0.0001	0.0016	0.1489	0.0026	0.0411	0.0038	0.0373
VEGF	0.9361	0.9361	0.9552	0.9607	0.9497	0.9337	0.9340	0.0028	0.0021	0.3020	0.1801	0.9514	0.9340	0.9328	0.9332	0.9591	0.2658	0.0301	0.0102	0.9361	0.0107	0.9340	0.2847	0.2205	0.2816
Akt	0.9761	0.9761	0.9630	0.9627	0.9760	0.9761	0.9761	0.0841	0.0148	0.3323	0.1667	0.9766	0.9761	0.9764	0.9761	0.9798	0.3404	0.0318	0.0364	0.9761	0.0684	0.9761	0.3238	0.3204	0.3164
AP2	0.2696	0.2696	0.8676	0.8675	0.2715	0.2682	0.0063	0.8619	0.8664	0.5741	0.6403	0.0101	0.0063	0.0065	0.0068	0.0002	0.5559	0.8661	0.8670	0.2696	0.8636	0.0063	0.5668	0.5328	0.5527
Bach1	0.8933	0.8933	0.8926	0.8924	0.8932	0.8933	0.8950	0.0155	0.0151	0.3455	0.0081	0.9019	0.8950	0.8933	0.8954	0.9399	0.4079	0.0141	0.0171	0.8933	0.0148	0.8950	0.2895	0.2799	0.2399
c-Jun	0.9470	0.9470	0.9434	0.9438	0.9468	0.9470	0.9471	0.0937	0.0156	0.4296	0.2949	0.9558	0.9471	0.9476	0.9470	0.9616	0.4406	0.0186	0.0019	0.9470	0.0372	0.9471	0.4072	0.4040	0.3975
HIF	0.9419	0.9419	0.9401	0.9395	0.9418	0.9419	0.9417	0.0011	0.0000	0.3125	0.0364	0.9426	0.9417	0.9418	0.9418	0.9429	0.3316	0.0001	0.0001	0.9419	0.0008	0.9417	0.2954	0.2831	0.2767
MAPK	0.9348	0.9348	0.9546	0.9594	0.9506	0.9291	0.8065	0.1090	0.0608	0.3084	0.3131	0.5270	0.8065	0.9316	0.7811	0.1176	0.3036	0.0666	0.0371	0.9348	0.0962	0.8065	0.3080	0.2972	0.3049
p53	0.0388	0.0388	0.0375	0.0505	0.0476	0.0386	0.0440	0.8045	0.9287	0.1054	0.4884	0.0501	0.0440	0.0395	0.0436	0.0365	0.0969	0.6820	0.7614	0.0388	0.7331	0.0440	0.1000	0.0959	0.1005
PKA	0.9464	0.9464	0.9431	0.9401	0.9451	0.9464	0.9461	0.0034	0.0017	0.4593	0.0425	0.9478	0.9461	0.9470	0.9461	0.9617	0.5009	0.0035	0.0033	0.9464	0.0037	0.9461	0.4279	0.4391	0.4110
PTEN	0.0203	0.0203	0.1096	0.1090	0.0205	0.0201	0.0001	0.7226	0.8412	0.5694	0.5659	0.0001	0.0001	0.0001	0.0001	0.0001	0.5616	0.9028	0.9160	0.0203	0.7759	0.0001	0.5742	0.5617	0.5674
HSPG2	0.0278	0.0278	0.0045	0.0072	0.1762	0.0317	0.2864	0.1563	0.0151	0.7981	0.1744	0.7545	0.2864	0.0370	0.3047	0.8791	0.6320	0.2769	0.2350	0.0278	0.2265	0.2864	0.5453	0.3162	0.3453
Integrin	0.1589	0.1589	0.9413	0.9417	0.1590	0.1593	0.1484	0.0993	0.0126	0.4104	0.3824	0.3938	0.1484	0.1013	0.1609	0.9274	0.3944	0.0032	0.0005	0.1589	0.0408	0.1484	0.3774	0.2999	0.3578
NGAL	0.4214	0.4214	0.4147	0.8858	0.8757	0.0203	0.4992	0.3413	0.3895	0.6120	0.8715	0.9383	0.4992	0.4233	0.4487	0.9384	0.4462	0.4827	0.0170	0.4214	0.3790	0.4992	0.5316	0.1917	0.4814
NOG	0.3292	0.3292	0.8532	0.8595	0.3307	0.3285	0.0570	0.0203	0.0147	0.0199	0.1285	0.1005	0.0570	0.0354	0.0656	0.0356	0.0236	0.0123	0.0210	0.3292	0.0243	0.0570	0.0280	0.0731	0.0633
Notch	0.0984	0.0984	0.0641	0.1117	0.1933	0.0984	0.1002	0.0546	0.0622	0.1365	0.1953	0.3737	0.1002	0.0611	0.1061	0.8576	0.1541	0.1118	0.0596	0.0984	0.0656	0.1002	0.1227	0.1412	0.1297
OPN	0.0271	0.0271	0.0127	0.0622	0.0516	0.0507	0.2184	0.1561	0.0202	0.2588	0.1367	0.1178	0.2184	0.0329	0.2754	0.1217	0.3612	0.2819	0.2358	0.0271	0.2264	0.2184	0.2307	0.3124	0.1746
SHH	0.3636	0.3636	0.8695	0.8691	0.3640	0.3633	0.1595	0.0039	0.0024	0.0757	0.0062	0.2088	0.1595	0.1103	0.1721	0.1026	0.1127	0.0021	0.0043	0.3636	0.0043	0.1595	0.0596	0.0812	0.0569
Wnt	0.1119	0.1119	0.0983	0.0994	0.1143	0.1105	0.1051	0.1103	0.0090	0.0977	0.1795	0.1134	0.1051	0.1128	0.1039	0.1222	0.1023	0.0398	0.1166	0.1119	0.1275	0.1051	0.0948	0.1116	0.1033
NR2F2	0.3310	0.3310	0.8681	0.8680	0.3320	0.3302	0.0496	0.0132	0.0000	0.0135	0.0546	0.0955	0.0496	0.0270	0.0585	0.0282	0.0244	0.0006	0.0156	0.3310	0.0203	0.0496	0.0105	0.0189	0.0127
RARs	0.0172	0.0172	0.0534	0.0530	0.0168	0.0172	0.0000	0.8538	0.8648	0.5059	0.5533	0.0000	0.0000	0.0000	0.0000	0.0000	0.4937	0.8640	0.8665	0.0172	0.8579	0.0000	0.4996	0.4768	0.4893
Connected Attractors & Times (Tot. 500 perturbations)	S1;106<br>S2;120<br>S5;143<br>S6;131	S1;130<br>S2;130<br>S5;127<br>S18;113	S1;104<br>S2;148<br>S10;118<br>S11;130	S1;127<br>S3;122<br>S9;122<br>S10;129	S1;260<br>S3;1<br>S4;236<br>S5;3	S2;265<br>S18;235	S4;226<br>S5;2<br>S6;248<br>S7;24	S4;19<br>S5;70<br>S12;255<br>S15;156	S4;8<br>S5;124<br>S13;167<br>S14;111<br>S15;90	S4;9<br>S7;2<br>S8;244<br>S12;245	S4;161<br>S12;145<br>S14;194	S4;254<br>S16;246	S4;238<br>S16;17<br>S18;245	S5;245<br>S18;255	S6;243<br>S16;257	S7;125<br>S8;129<br>S16;138<br>S17;108	S8;254<br>S12;246	S12;247<br>S13;3<br>S14;250	S12;255<br>S15;245	S1;118<br>S2;138<br>S5;111<br>S18;133	S4;10<br>S5;194<br>S12;235<br>S14;2<br>S15;59	S4;227<br>S5;1<br>S6;251<br>S16;21	S4;24<br>S6;3<br>S7;218<br>S12;255	S7;250<br>S12;250	S4;167<br>S6;4<br>S7;87<br>S12;242

Supplementary Table VIII. Selected Combinations of Inducing S1 and S4 to Others.

Starting Attractor	Node 1 (1 up-regulate; 0 down-regulate)		Node 2 (1 up-regulate; 0 down-regulate)		Node 3 (1 up-regulate; 0 down-regulate)		Arriving Attractor
S1	IFN-γ	0	Stat1	0			S2
S1	IFN-γ	0	TGF-β	1			S2
S1	Stat1	0	TGF-β	1			S2
S1	Runx1	1					S3
S1	Stat5	0					S3
S1	Runx1	1	Hes1	1	Notch	1	S4
S1	Runx1	1	Cdx2	1	SHH	0	S4
S1	Runx1	1	BMP	1	SHH	0	S4
S1	Runx2	1	Cdx2	1	SHH	0	S4
S1	Runx2	1	BMP	1	SHH	0	S4
S1	Stat5	0	Cdx2	1	SHH	0	S4
S1	Stat5	0	BMP	1	SHH	0	S4
S1	Stat5	0	p53	1	SHH	0	S4
S1	Stat5	0	Hes1	1	TGF-β	1	S4
S1	Stat5	0	Hes1	1	Notch	1	S4
S1	Stat3	0					S5
S1	PTEN	1					S5
S1	IFN-γ	0	Stat1	0	Stat3	0	S6
S1	IFN-γ	0	Stat1	0	PTEN	1	S6
S1	IFN-γ	0	Stat3	0	TGF-β	1	S6
S1	IFN-γ	0	TGF-β	1	PTEN	1	S6
S1	Stat1	0	Stat3	0	TGF-β	1	S6
S1	Stat1	0	TGF-β	1	PTEN	1	S6
S1	E2F	0	Runx1	1			S9
S1	E2F	0	Stat5	0			S9
S1	p21	1	Runx1	1			S9
S1	p21	1	Stat5	0			S9
S1	E2F	0					S10
S1	p21	1					S10
S4	Runx1	0	Stat5	1	SHH	1	S1
S4	SHH	1					S3
S4	Runx1	0	Runx2	0	Stat5	1	S5
S4	Stat1	0					S7
S4	TGF-β	1					S7
S4	E2F	0	Myc	0	Stat1	0	S8
S4	E2F	0	Myc	0	TGF-β	1	S8
S4	HIF	0	p53	1	PTEN	1	S8

We enumerate all possible combinations of selecting 1, 2, or 3 nodes with up-regulation (1) or down-regulation (0), and then follow the trajectory to see which attractor it will reach. The total number of runs are $2C_{81}^1 = 162$, $2^2C_{81}^2 = 12960$, and $2^3C_{81}^3 = 682560$. Due to the stability of an attractor, most perturbations lead back to the original attractor, we list here the rare combinations go to other attractors. More data can be seen from Supplementary File 3.
